# Challenges of Aufheben to Promote Druglikeness: Chemical Modification Strategies to Improve Aqueous Solubility and Permeability

**DOI:** 10.1021/acs.jmedchem.5c00287

**Published:** 2025-10-08

**Authors:** Minoru Ishikawa, Shusuke Tomoshige, Shinichi Sato

**Affiliations:** 1 Graduate School of Life Sciences, 13101 Tohoku University, 2-1-1 Katahira, Aoba-ku, Sendai 980-8577, Japan; 2 Frontier Research Institute for Interdisciplinary Sciences, 13101 Tohoku University, 6-3 Aramaki aza-Aoba, Aoba-ku, Sendai 980-8578, Japan

## Abstract

A key challenge in drug discovery is to endow candidate bioactive molecules with druglike properties. However, the optimization of critical, often-conflicting physicochemical properties through chemical modifications is particularly difficult, and the term Aufheben has been adopted to describe the simultaneous preservation and modification of apparent opposites to achieve improvement. This perspective examines strategies to reconcile conflicting parameters, such as lipophilicity/hydrophilicity, druglikeness/flatness, and druglikeness/molecular weight, in order to improve the aqueous solubility and membrane permeability of drug candidates. We review and categorize numerous molecular design strategies that have been investigated to address these challenges and highlight some recent successful examples.

## Graphical abstract

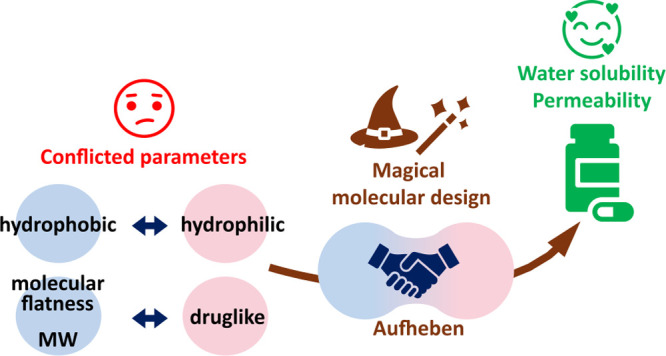

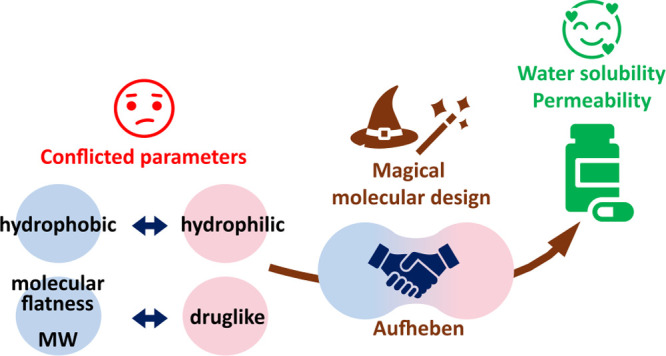

## 1 Significance

- Medicinal chemistry requires reconciliation of apparently conflicting physicochemical parameters to achieve druglike properties.
- We summarize molecular design strategies to simultaneously improve aqueous solubility and membrane permeability of drug candidates.
- Recent examples of successful strategies to enhance druglikeness of small molecules as well as beyond rule of five molecules, especially cyclic peptides and PROTACs, are provided.

## 2 Introduction

Druglikeness is a qualitative measure of a candidate compound’s suitability for development as a drug, especially its likely oral bioavailability, and is evaluated in terms of the molecular structure and physical properties, such as molecular weight (MW), lipophilicity, and numbers of hydrogen bond donors (HBDs) and acceptors (HBAs). For example, the original Rule of Five (Ro5) predicts that compounds with a MW ≤ 500, Log*P* (the common logarithm of partition coefficient) ≤ 5, HBDs ≤ 5, and HBAs ≤ 10 are likely to be orally bioavailable.^1^ These physicochemical parameters of Ro5 are recognized as being associated with acceptable values of both aqueous solubility in the intestine and intestinal membrane permeability. More recently, however, drugs with parameters beyond Ro5, designated as bRo5, have been successfully developed. Although various definitions for bRo5 molecules exist, we define bRo5 here as compounds with a MW ranging from 500 to 3000, thereby excluding biopharmaceuticals such as protein, antibody, and nucleic acid drugs. In such cases, it is particularly challenging to achieve an appropriate balance of the aqueous solubility and permeability. The identification of numerous important druglike parameters often leads medicinal chemists to contend with the conflicting relationships among them. Achieving a delicate balance among these interdependent properties is crucial for successful drug candidates, yet it presents a significant challenge in molecular design.

The importance of both aqueous solubility and permeability is reflected in the Biopharmaceutics Classification System (BCS),^2^ which classifies drugs with the aim of identifying compounds with minimal absorption-related risks. The BCS classifies compounds into four categories. Class 1 compounds exhibit high permeability and high solubility, ensuring efficient absorption after oral administration, whereas Class 4 compounds are characterized by both low solubility and low permeability. Class 2 exhibits low solubility–high permeability, while Class 3 shows high solubility–low permeability.

Achieving adequate permeability of orally administered drugs requires an optimal level of lipophilicity to facilitate absorption across the lipid membranes. In the early stage of drug development, the parallel artificial membrane permeability assay (PAMPA) and Caco-2 assay are commonly employed for *in vitro* permeability prediction. Apparent permeability (*P*_app_) values are classified as poor (<1.0 × 10^–6^ cm/s), moderate (1–10 × 10^–6^ cm/s), or good (>10 × 10^–6^ cm/s) according to the literature.^3^ However, high lipophilicity, together with low aqueous solubility, has become an increasingly common characteristic of hits, leads, development candidates, and even marketed drugs.^4^ This trend may be attributed to druggable binding pockets tending to have hydrophobic environments.

Aqueous solubility is also a critical physicochemical property for small-molecule drug candidates. The dose number (Do) is a parameter used to evaluate the practical adequacy of drug solubility.^5^ It is calculated as the dose divided by an uptake volume of 250 mL and the drug’s thermodynamic solubility: Do = (dose/uptake volume [250 mL]) /thermodynamic solubility. A drug candidate is considered sufficiently soluble when the Do is less than 1. Orally administered drugs must exhibit sufficient aqueous solubility to dissolve in the gastrointestinal tract, as their absorption via passive diffusion depends on the concentration gradient between the intestinal lumen and the bloodstream, a factor influenced by solubility. Furthermore, the efficacy evaluation and risk assessment of poorly soluble compounds present significant challenges. Overall, drug candidates must balance apparently contradictory physicochemical properties, namely, lipophilicity and hydrophilicity (aqueous solubility). Medicinal chemists often struggle with the conflicting relationship between water solubility and membrane permeability. Here, we adopt the term Aufheben, used by Hegel to describe the dialectical synthesis or combination of a thesis and its antithesis, to denote both preserving and modifying opposing physicochemical characteristics to achieve a simultaneous improvement of aqueous solubility and membrane permeability.

First, we consider the solubility in water. The Gibbs-free energy of dissolution (Δ*G*_*sol*_) is determined by the following equation: Δ*G*_sol_ = Δ*H*_sol_ – *T*Δ*S*_sol_. Dissolution is thermodynamically favorable when Δ*G*_sol_ < 0. The enthalpy of dissolution (Δ*H*_sol_) and entropy of dissolution (Δ*S*_sol_) are the key driving factors at a given temperature (*T*). The dissolution of a solid molecule in water generally proceeds through three conceptual steps, as illustrated in Figure 1. Releasing a molecule from its crystal lattice (step 1) requires a substantial amount of enthalpy (Δ*H*_1_) and an increase in entropy (Δ*S*_1_) due to the disruption of ordered packing. This step is largely governed by the crystal packing efficiency and the intermolecular forces within the solid. The melting point of a solute crystal is a key indicator of Δ*H*_1_ reflecting the energy required to overcome these lattice forces. Differential scanning calorimetry (DSC) is an experimental technique used to measure the melting point, enthalpy of fusion (Δ*H*_fus_), and entropy of fusion (Δ*S*_fus_), which directly reflect the stability of the crystalline state and the energy barrier to dissolution. Next, creating a void in water to accommodate the solute (step 2) involves an increase in both the enthalpy (Δ*H*_2_) and entropy (Δ*S*_2_) of the water, as water molecules must rearrange. The free solute occupies the cavity in water (step 3), leading to favorable interactions (e.g., hydrogen bonding, van der Waals forces) between the solute and water molecules. This step typically results in a decrease in both the enthalpy (Δ*H*_3_) and entropy (Δ*S*_3_) as the system becomes more ordered around the solute. Thus, the overall solubility of a solid solute in water is influenced by several factors: the crystallinity of the solute, its molecular size, and its ability to interact favorably with water.^6^ The total enthalpy of dissolution is Δ*H*_sol_ = Δ*H*_1_ + Δ*H*_2_ + Δ*H*_3_, and the total entropy of dissolution is Δ*S*_sol_ = Δ*S*_1_ + Δ*S*_2_ + Δ*S*_3_. The long-standing principle of solvation, often encapsulated in the phrase “like dissolves like”, can be thermodynamically explained by a significant decrease in Δ*H*_3_ and Δ*S*_3_ primarily due to strong hydrogen bonding between the solute and water molecules. Furthermore, Δ*H*_sol_ and Δ*S*_sol_ can be experimentally obtained by measuring the mole fraction solubility (*X*) at different temperatures and applying the Van’t Hoff equation: lnX = – Δ*H*_sol_/ RT + Δ*S*_sol_/*R*, where *R* represents the ideal gas constant.

**Figure 1:**
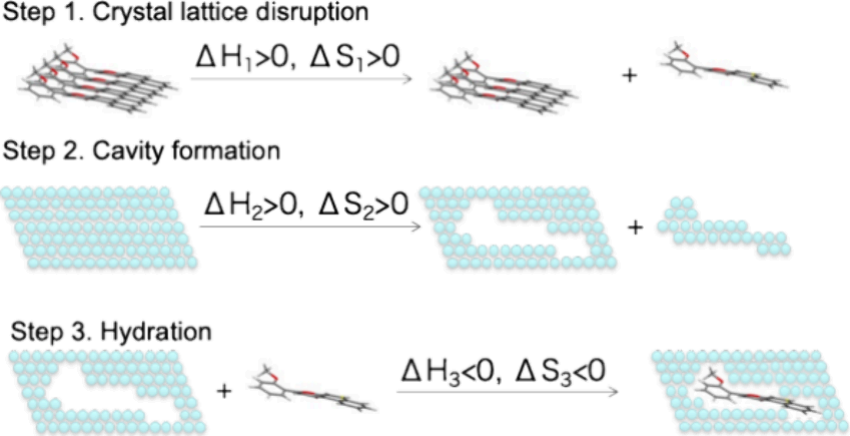
Schematic illustration of the three steps involved in drug dissolution.

A molecule with higher MW necessitates a larger void in water, thereby requiring more hydrogen bonds to be disrupted leading to a large increase of Δ*H*_2_ and Δ*S*_2_. To overcome this, large molecules can achieve both solubility and permeability through environment-responsive conformational changes. These molecules adopt an open conformation in aqueous environments, exposing their polar functional groups to enhance solubility. Conversely, in lipophilic environments, they transition to a closed conformation, masking their polar groups via intramolecular interactions, such as intramolecular HB, thereby facilitating permeability. This behavior is commonly termed molecular chameleonicity (Figure 2).

**Figure 2:**
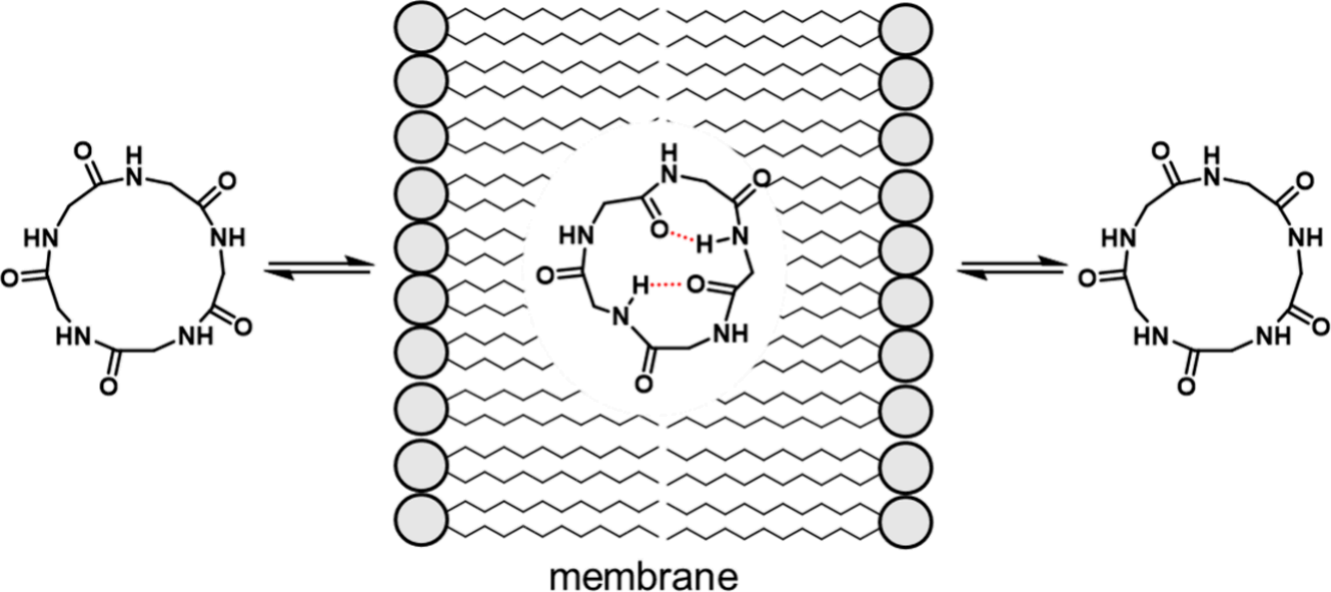
Molecular chameleonicity.

In modern drug discovery and development, solubility is evaluated in various ways, depending on the stage of development.^7^ *In silico* prediction is utilized during the early stage of drug discovery and can help prioritize the synthesis of hit compounds identified through high-throughput screening. However, accurately predicting aqueous solubility remains challenging, in part because of the variability in data sources used for prediction—aqueous solubility data are often not comparable, with marked differences in reported values and evaluation methods. Kinetic solubility is typically measured starting with a solution of a compound in DMSO, with precipitation detected after a short incubation period using turbidity or UV absorption measurement. This method is advantageous during the early stages of drug discovery due to its high throughput, suitability for automation, and minimal compound consumption. However, the kinetic solubility does not take account of the crystalline form of the compound. For example, compounds in an amorphous state can be up to 100 times more soluble than crystalline forms.^7^

In contrast, thermodynamic solubility (equilibrium solubility) refers to the solubility of the most stable crystalline form of a compound in equilibrium with the solvent. The most stable form is invariably the form with the highest melting point. The significance of the thermodynamic solubility assessment becomes greater during the late discovery stage and early development of candidate drugs. While formulation technologies can enhance the dissolution rate and induce a temporary or apparent increase in solubility, they cannot permanently change the compound’s inherent solubility. Given sufficient time, any undissolved solute will revert to its most stable crystal form under the given conditions, and the solubility will ultimately converge to the true thermodynamic solubility.^8,9^ In addition, the amorphous form tends to spontaneously recrystallize during storage, and it is challenging to predict the time required for nucleation and crystal growth.^10^ Thus, it is preferable to generate drug candidates (active pharmaceutical ingredients) with sufficient aqueous solubility through medicinal chemistry efforts.

As illustrated in Figure 1, the solubility of a solid solute in water depends on several factors: the crystallinity of the solute, its molecular size, and its hydration. Among these, the hydration step (step 3) is a promising target to improve aqueous solubility. Reducing hydrophobicity (Log*P*_ow_), distribution coefficient (Log*D*), or topological polar surface area (tPSA) by means of chemical modifications, such as the introduction of hydrophilic group(s), is a classical and widely adopted strategy for improving aqueous solubility. For instance, the replacement of a phenyl ring with heterocyclic rings has been extensively studied.^11,12^ Lipophilic substituent constants (π), as defined by Hansch et al., are valuable guides in molecular design for modifying the hydrophilicity of parent molecules. However, this “like dissolves like” strategy is not universally effective, as the introduced hydrophilic group(s) may interfere with the target protein–drug interaction. More critically, a reduction in lipophilicity often results in a trade-off between the solubility increase and permeability decrease. This inherent limitation of the like dissolves like approach poses a significant challenge.

The solubility of a solid solute in water is also influenced by the crystal packing of the solute (Figure 1, step 1) and compounds that exhibit poor solubility due to strong crystal packing interactions are often referred to as “brick dust”. Over the past decade or two, various strategies aimed at improving the aqueous solubility of traditional pharmaceutical compounds by disrupting intermolecular interactions have been reported. In parallel, bRo5 molecules, including cyclic peptides^13^ and PROTACs (proteolysis targeting chimeras),^14^ have emerged as promising therapeutic modalities. These molecules are generally less soluble and less permeable than traditional pharmaceutical compounds due to their higher MW (Figure 1, step 2). However, examples of bRo5 molecules with high solubility and permeability have been reported, and their structural and physicochemical characteristics are beginning to be elucidated.

Here, we review recent progress in endowing molecules with druglikeness, focusing on strategies to increase aqueous solubility and membrane permeability. Several excellent reviews on aqueous solubility^15–18^ and druglike physicochemical properties^11^ have been published recently, and this perspective differs from them in three main respects focusing on Aufheben. First, we highlight examples of strategies to achieve simultaneous improvements in seemingly contradictory pairs of physicochemical properties, such as increasing both lipophilicity and hydrophilicity (aqueous solubility) through chemical modifications that weaken intermolecular interactions. Increasing evidence suggests that such chemical modifications can enhance not only aqueous solubility but also permeability. Second, we present examples illustrating how to optimize the aqueous solubility of flat molecules, which are often considered to lack druglike properties. These first two differentiating points, which primarily focus on methodologies for more traditional pharmaceutical compounds, are detailed in Section 3. Third, we examine strategies to improve the membrane permeability and aqueous solubility of bRo5 molecules. This third aspect is elaborated upon in Section 4.

## 3 Improvement of Aqueous Solubility and Permeability of Small Molecules by Disrupting Intermolecular Interactions (Aufheben of Lipophilicity and Aqueous Solubility)

As the aqueous solubility of a compound is influenced by the molecular packing in the solid state (Figure 1, step 1), a possible strategy to improve the aqueous solubility involves disruption of the tight crystal packing of molecules. In other words, molecular modifications that weaken the intermolecular interactions in the most stable crystal form of a compound can enhance its thermodynamic solubility. The melting point of a compound is closely related to its crystal lattice and crystal packing energy,^10^ making it a useful parameter for assessing crystal packing, along with crystal density. In 1980, Yalkowsky proposed a general solubility equation, derived through semiempirical analysis, to describe the relationship between aqueous solubility and melting point: Log­[solubility (M)] = 0.5 – (Log*P*) – 0.01­[[melting point­(°C)] – 25].^19^ This equation was primarily based on data for rigid, polycyclic, and halogenated aromatic compounds. In 2009, Lovering et al. proposed that increasing molecular saturation, quantified by the fraction of sp^3^-hybridized carbons (Fsp^3^) (defined as the number of sp^3^ carbons divided by the total carbon count of a compound), is an approach to improving clinical success.^20^ They also demonstrated through database analysis that Fsp^3^ positively correlated with solubility and negatively correlated with the melting point. It is worth noting, however, that the precise relationships between aqueous solubility and the melting point of pharmaceutical compounds remained a lacuna in the literature at that time. Regarding the relationship between molecular structures and the aqueous solubility of pharmaceutical compounds, we previously proposed that disruption of molecular planarity—including not only increased saturation but also an increase in the dihedral angle of planar compounds and molecular bending—decreases melting points and increases thermodynamic aqueous solubility, as shown by matched molecular pair analyses.^21^ Furthermore, we demonstrated that the introduction of hydrophobic substituents also improves thermodynamic aqueous solubility, realizing part of the phenomenon of “Aufheben”. In the past decade or two, various chemical modification strategies have been developed to improve the aqueous solubility of complex pharmaceutical compounds by disrupting intermolecular interactions, and as will be discussed later, these approaches can increase aqueous solubility even in cases where hydrophobicity is simultaneously increased.

### 3.1 Case Studies On Modifying Flatland (Aufheben of Flatland and Druglikeness)

#### 3.1.1 Disruption of Molecular Planarity by *ortho*-Substitution of Biaryl Groups

As the medicinal chemistry toolbox has evolved, numerous bioactive molecules possessing biaryl groups have been reported, likely for two reasons: (1) they serve as versatile scaffolds, and (2) they can be easily synthesized by means of Suzuki coupling reactions.^22^ Notably, the AstraZeneca screening collection contained 2% biphenyl fragments in 1990, but this had increased to 12% of all registered compounds by 2014.^22^ However, biaryl molecules often exhibit low solubility due to their tight crystal packing and are often regarded as undruglike.

The effects of *ortho*-substitution on biaryl groups are exemplified by agonists of peroxisome proliferator-activated receptor (PPAR), a type of nuclear receptor (Table 1).^23^ An initial strategy to improve the aqueous solubility of PPAR agonists was the introduction of hydrophilic groups. Unfortunately, several such modifications diminished the PPAR agonistic activity (e.g., **2a** vs **2d**), as many nuclear receptors recognize the hydrophobicity of the ligands. In contrast, the introduction of a hydrophobic group(s) at the *ortho*-position of the biaryl group increased the PPAR agonistic activity (e.g., **1a** vs **1b**/**1c**, **2a** vs **2b**/**2c**, and **2d** vs **2e**). All of the analogs listed in Table 1 exhibited greater thermodynamic solubility than their parent compounds. Interestingly, an increase in dihedral angle (**2c**) resulted in greater solubility in phosphate buffer than a decrease in hydrophobicity (**2d**) in this series. Notably, **2e** demonstrated a 350-fold higher solubility than **2d** in phosphate buffer. Mechanistically, all compounds with a methyl group(s) introduced at the *ortho*-position of the biaryl moiety (**1b**, **2b** and **2e**) exhibited increased hydrophobicity, larger dihedral angle, and lower melting point compared to their parent compounds (**1a**, **2a**, and **2d**, respectively). The most soluble analogue **2e** showed the lowest melting point and the largest dihedral angle in this series. When comparing **2e** with **2a**, two modifications (replacement of the phenyl group with a pyridine ring and introduction of methyl groups) resulted in a more than 2700-fold increase in solubility in phosphate buffer. These findings suggest that employing a combination of strategies can be effective to improve the aqueous solubility. A scatter plot (Figure 3) illustrates the relationships among PPAR agonistic activity and CLogP, as well as the impact of *ortho*-substitution on solubility improvement.

**Table 1:**
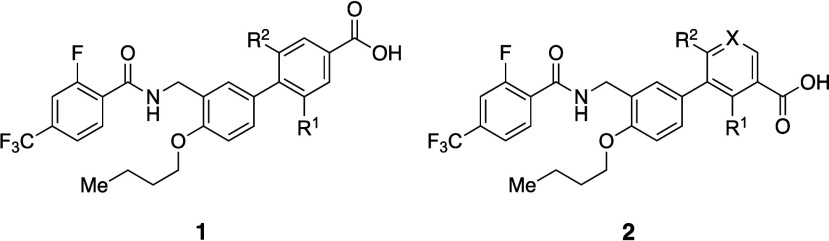
Improvement in Thermodynamic Aqueous Solubility and Biological Activity by *ortho*-Substitution of Biaryl Group

				thermodynamic aqueous solubility (pH 7.4) (mg mL^–1^)						
compd	*R* ^1^	*R* ^2^	*X*	50% EtOH^a^	phosphate buffer (pH 7.4)	CLogP	HLPC retention time (min)^b^	melting point (°C)	calculated dihedral angle (°)	PPARδ EC_50_ (nM)
**1a**	H	H		0.375	<0.001	6.3	7.98	259–262	43.5	170
**1b**	Me	H		0.985	<0.001	6.8	9.46	241–243	52.5	11
**1c**	F	H		3.22	<0.001	7.1	8.72	221–223	36.1	53
**2a**	H	H	CH	1.35	<0.001	6.4	7.79	177–178	36.9	29
**2b**	Me	H	CH	9.95	<0.001	6.9	8.65	146–149	57.5	1.6
**2c**	F	F	CH	10.4	0.0217	7.5	7.42	177	46.2	5.7
**2d**	H	H	N	9.03	0.00762	4.8	3.61	152	37.4	220
**2e**	Me	Me	N	17.7	2.70	5.8	4.36	104–106	78.1	76

a Thermodynamic solubility in a mixture of phosphate buffer (pH 7.4) and EtOH.

b Reversed-phase column.

**Figure 3:**
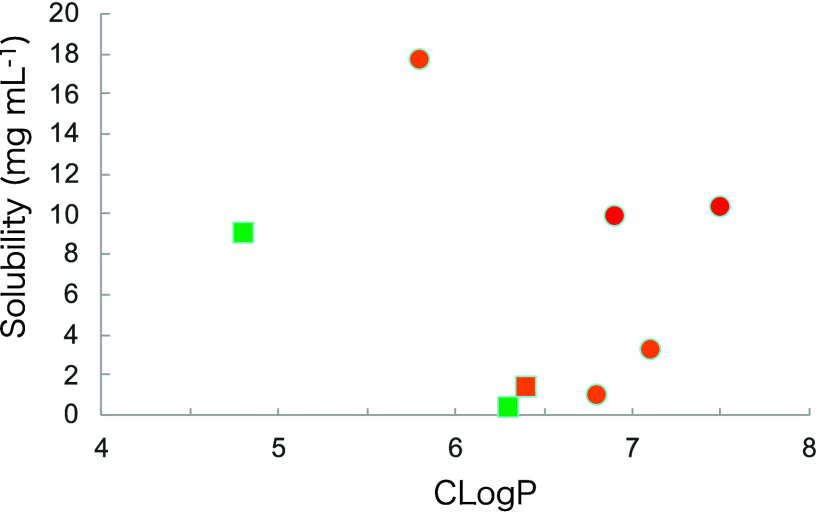
Aqueous solubility versus CLogP. Data points are colored by pEC_50_ ranges (6–7 (green), 7–8 (orange), 8–9 (red)). Circle symbols denote *ortho*-substituted compounds, while square symbols indicate non-substituted compounds.

The effects of *ortho*-substitution of biaryl groups are also exemplified in β-naphthoflavone **3a**, which is an aryl hydrocarbon receptor agonist (Table 2). The thermodynamic solubility of the *ortho*-methyl analog **3b** (262 μg/mL) was 3-fold greater than that of **3a**.^24^ Furthermore, the position of the methyl group significantly influenced the solubility: the rank order of aqueous solubility was *ortho* (**3b**) ≫ nonsubstituted (**3a**) ≥ *meta* (**3c**) > *para* (**3d**).^25^ Several mechanistic studies support the idea that *ortho*-substitution disrupts molecular planarity by increasing the dihedral angle, which leads to a lower melting point and, consequently, higher solubility. Notably, the *ortho*-dimethyl analog **3e** exhibited a 15-fold higher solubility (1270 μg/mL) than **3a**, despite its increased hydrophobicity. The solubility was also dependent on the number of methyl group(s): the rank order was dimethyl (**3e**) > monomethyl (**3b**) > nonsubstituted (**3a**). This trend aligns with the order of lower melting points, larger calculated dihedral angles, and lower λmax values. On the other hand, pyridine analogue **3f**, which lacks a hydrogen atom, showed a higher melting point, higher λmax, and decreased dihedral angle compared to **3a**. Nevertheless, **3f** demonstrated an improved aqueous solubility (299 μg/mL), likely due to its reduced hydrophobicity. Interestingly, as observed in prior examples, the increase in the dihedral angle (**3e**) resulted in a greater solubility improvement than the reduction in hydrophobicity (**3f**).

**Table 2:**
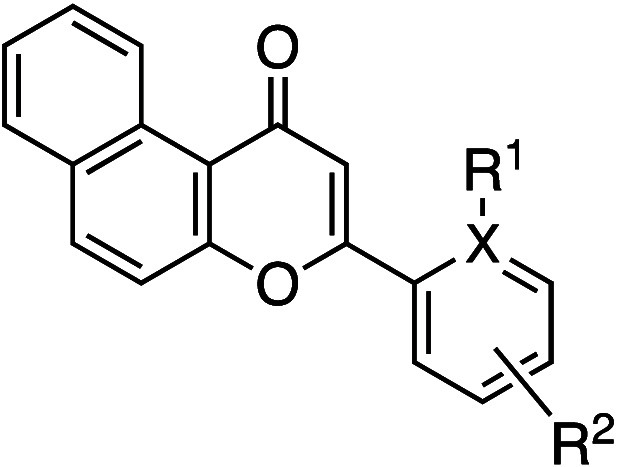
Improvement in Thermodynamic Aqueous Solubility by *ortho*-Substitution

compd	*R* ^1^	*R* ^2^	*X*	thermodynamic aqueous solubility (pH 7.4) (μg mL^–1^)^a^	ClogP	HLPC retention time (min)^b^	melting point (°C)	calculated dihedral angle (°)	λmax (nm)
**3a**	H	H	C	84.6	4.8	7.24	165–167	17.8	273
**3b**	H	*o*-Me	C	262	5.3	8.17	135–137	37.9	265
**3c**	H	*m*-Me	C	80.9	5.3	9.44	162	16.8	274
**3d**	H	*p*-Me	C	35.4	5.3	9.43	194–195	16.5	285
**3e**	Me	*o*-Me	C	1270	5.7	9.13	92	70.0	261
**3f**		H	N	299	3.3	4.52	187–188	0	285

a Thermodynamic solubility in phosphate buffer (pH 7.4): EtOH (1:1).

b Reversed-phase column.

Researchers at Novartis reported GNF6702 (**4a)** as an antiparasitic drug candidate (Table 3).^26^ However, the clinical progression of **4a** was hindered due to solubility-limited oral absorption. Compound **4a** features a flat molecular structure and a high melting point (224 °C), characteristics contributing to its low aqueous solubility. To address this issue, a methyl group was introduced at the *ortho*-position of the biaryl group (**4b**), resulting in a reduced melting point (139 °C) and improved aqueous solubility, despite the increased lipophilicity.^27^ When compared to the free base form of **4a**, the fumarate cocrystal of compound **4b** afforded a 5-fold higher supersaturation in FaSSIF, achieving a concentration of 73 μM. Moreover, the fumarate salt of **4b** demonstrated superior oral bioavailability in mice (46%) compared with **4a** (34%). In other models, the oral bioavailability of fumarate salt **4b** was 67% in rats, 44% in dogs, and 27% in monkeys.

**Table 3:**
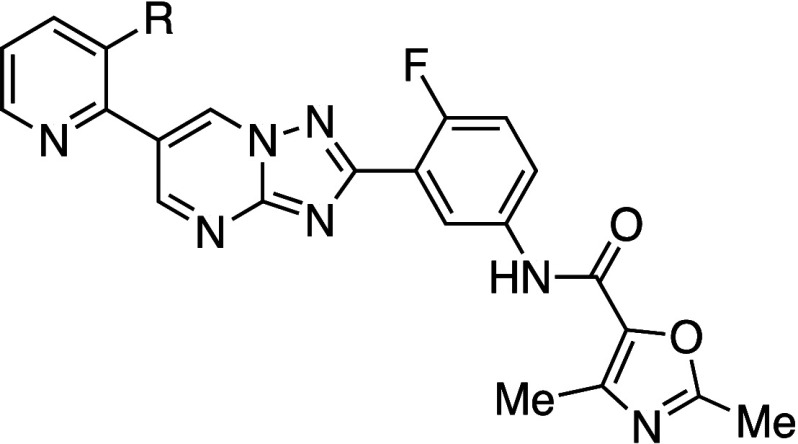
Improvement in Aqueous Solubility and Oral Bioavailability by *ortho*-Substitution

compd	*R*	aqueous solubility (pH 6.8) (μM)^a^	CLogP^b^	melting point (°C)	mouse *F* (%)
**GNF6702 (4a)**	H	10	–0.29	224	34
**4b**	Me	17	–0.086	139	46

a Solubility in a high-throughput assay format.

b CLogP was estimated by us, using ChemDraw version 20.0.

*ortho*-Substitution has remarkable potential for enhancing aqueous solubility, achieving an over 100-fold increase (Table 4).^28^ Wnt/β-catenin signaling inhibitor **5a** exhibited a limited kinetic solubility of 0.05 μg/mL in PBS buffer. Quantum mechanical analysis of **5a** revealed a highly planar conformation among the pyrazole ring, amide group, and quinoline ring. To address this limitation, a methyl group was introduced into the pyrazole ring (**5b**), effectively disrupting the molecular planarity by increasing the dihedral angle. This chemical modification resulted in a 120-fold improvement in aqueous solubility compared to **5a**.

**Table 4:**
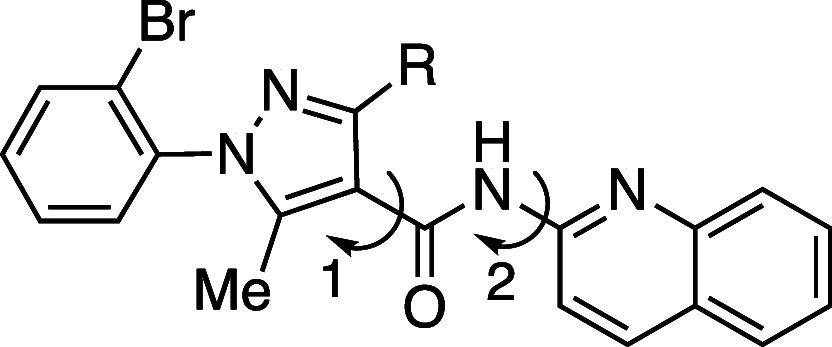
Improvement in Aqueous Solubility by *ortho*-Substitution

compd	*R*	kinetic aqueous solubility (pH 7.4) (μg mL^–1^)^a^	CLogP^b^	dihedral angle 1 (°)^c^	dihedral angle 2 (°)^c^
**5a**	H	0.05	3.4	171.4	178.5
**5b**	Me	5.9	3.3	166.6	178.7

a Kinetic solubility of solid at 25 °C for 6 h.

b CLogP was estimated by us, using ChemDraw version 20.0.

c Quantum mechanics analysis.

Researchers at Merck reported the interesting phenomenon that atropisomers showed greater aqueous solubility than the racemate (Table 5).^29^ For example, the kinetic aqueous solubilities of enantiomeric atropisomers **6P** and **6M** were greater than that of racemate **6**. A key observation was the higher melting point of racemate **6** (261 °C) compared to **6M** (224 °C), suggesting weaker intermolecular interactions in the atropisomeric crystal structures. However, during the 24 h incubation, the solid-state forms of both atropisomers transitioned from anhydrate to hydrate form.

**Table 5:**
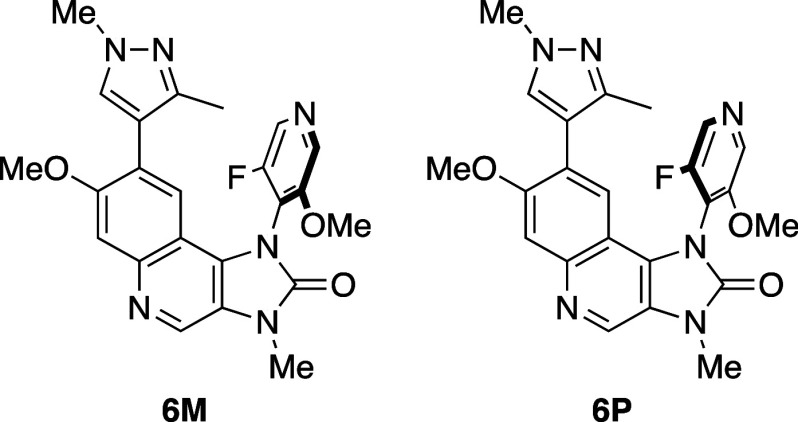
Improvement in Aqueous Solubility of *ortho*-Substituted Atropisomers

	kinetic aqueous solubility (μg mL^–1^)^a^					
compd	PBS pH 7.4	FaSSIF^b^ pH 6.5	FaSSIF^b^ pH 5.0	CLogP^c^	melting point (°C)	crystal density (g cm^–3^)
**rac-6**	20	54	193	1.4	261	1.354
**6M**	93	211	773	1.4	224	1.372
**6P**	95	235	741	1.4		1.374

a Kinetic solubility of solid at 37 °C for 2 h.

b Fasted state simulated intestinal fluid.

c CLogP was estimated by us, using ChemDraw Ultra 20.0.

Single-crystal X-ray diffraction (XRD) analysis of the anhydrate forms revealed densities of 1.374 g/cm^3^ for **6P**, 1.372 g/cm^3^ for **6M**, and 1.354 g/cm^3^ for racemate **6**. However, the authors noted that lattice energy and free energy are not directly correlated with the density of a specific solid-state form. They emphasized the importance of considering entropic effects, as these are reflected in both the melting point and solubility of a compound.

Effects on *ortho*-substitution have also been demonstrated in unfused bicyclic aryl systems. Efforts to improve the solubility of integrin antagonist **7a** initially focused on introducing hydrophilic substituents (**7b**–**7d**). However, these modifications resulted in decreased inhibitory activity in a cell-based assay using vascular smooth muscle cells (VSMCs) (Table 6).^30^

**Table 6:**
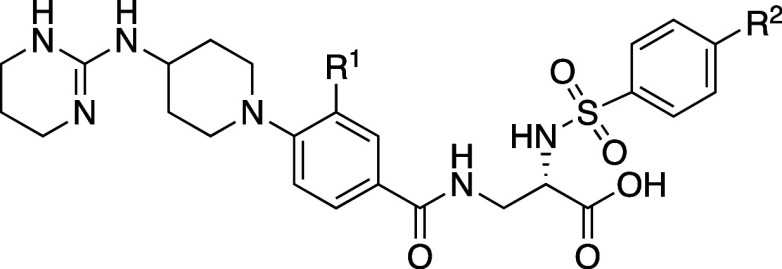
Improvement in Aqueous Solubility and Biological Activity by *ortho*-Substitution of the Unfused Bicyclic Phenyl System

Compd	R^1^	R^2^	thermodynamic water solubility (mg mL^–1^)	CLogP	HLPC retention time (min)^a^	melting point (°C)	α_v_β_3_IC_50_ (nM)	VSMC IC_50_ (nM)^b^
**7a**	H	H	<0.1	1.1	8.25	252–254	1.3	190
**7b**	OH	H					0.44	530
**7c**	F	CO_2_H					0.77	660
**7d**	OH	OH					0.30	390
**7f**	F	H	0.6	1.7	9.73	182–184	0.36	48
**7g**	OMe	H	1.3	0.79	8.72	162–164	0.19	110
**7h**	F	OH	0.1	1.4	6.16	193–197	0.14	53

a Reversed-phase column.

b α_v_β_3_-Mediated cell adhesion assay using human vascular smooth muscle cells (VSMC) and human vitronectin.

In contrast, a second approach involving the introduction of hydrophobic substituents (**7f** and **7g**) led to enhanced activity in both receptor-binding and VSMC assays. Furthermore, these compounds exhibited significantly increased thermodynamic aqueous solubility, with **7f** and **7g** being at least 6-fold and 13-fold more soluble than parent compound **7a**, respectively.

As for the mechanism of the increase in solubility of **7f** and **7g** compared with **7a**, a relationship between the rank order of aqueous solubility and the order of melting points indicates that the increase in aqueous solubility of **7f** and **7g** was caused by disruption of molecular planarity, leading to a decrease of intermolecular interactions despite an increase of hydrophobicity. Additionally, the single-crystal X-ray structure of **7f** revealed a substantial increase in the dihedral angle between the piperidine ring and the benzoyl group, corroborating the hypothesis of disrupted molecular planarity. Interestingly, hydroxyl analogue **7h**, despite its lower hydrophobicity compared with **7f**, exhibited decreased solubility. This decrease is consistent with its higher melting point, suggesting that the introduced hydroxyl group may have formed new intermolecular HBs, resulting in tighter crystal packing. These findings highlight the superior impact of disrupting molecular planarity (**7f** and **7g**) on improving aqueous solubility, compared to merely decreasing the hydrophobicity (**7h**).

Researchers at Bayer identified ATR kinase inhibitor **8a**, which showed low kinetic aqueous solubility (<1 μg/mL), resulting in an oral bioavailability of only 14% in rats, even though **8a** has a high permeability coefficient of 102 nm/s in Caco-2 cells.^31^ Moreover, the compound also raised safety concerns due to its activity in the hERG patch clamp assay *in vitro* (IC_50_: 5.8 μM). To reduce intermolecular interaction and planarity, (1) the (methylsulfonyl)­phenyl group in **8a,** which is capable of head-to-head interaction (vide infra in Section 3.5),^32^ was replaced with 1-methylpyrazole (equivalent to *ortho*-substitution), and (2) a methyl group was introduced at the 3-position of the morpholinyl group (equivalent to *ortho*-substitution) in **8a**. Lead compound **8a** and designed **8b** have an identical Log*D* value of 2.1, but **8b** showed more than 34-fold-increased aqueous solubility. In the Caco-2 model, **8b** showed a permeability coefficient of 211 nm/s, and after oral administration, the bioavailability ranged from moderate in dogs (51%) to high in rats (87%). In the hERG patch clamp assay *in vitro*, **8b** showed no activity (IC_50_: >10 μM). The X-ray structure of **8b** showed a twist between the naphthyridine scaffold and the 1-methylpyrazolyl group (torsion angle: 54.8°) (Figure 4). Additionally, the (3*R*)-methyl substituent of the morpholine points out the plane of the naphthyridine scaffold (Table 7).

**Table 7:**
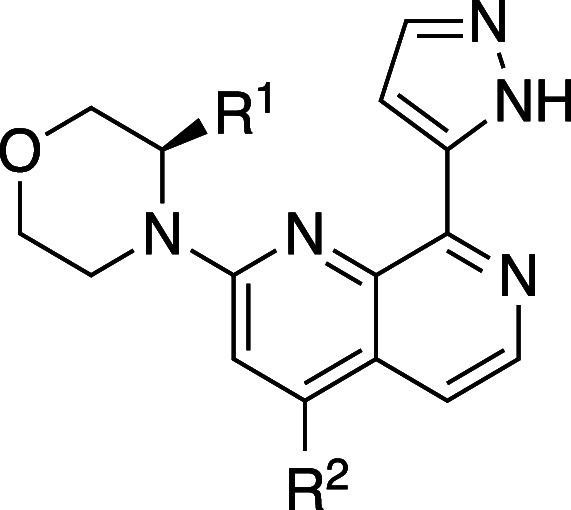

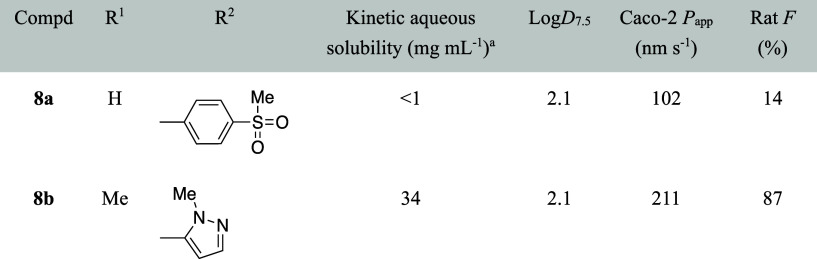
Improvement in Aqueous Solubility, Oral Bioavailability, and HERG Inhibition by *ortho*-Substitution of the Unfused Bicyclic Aryl System

a Solubility in a high-throughput assay format.

**Figure 4:**
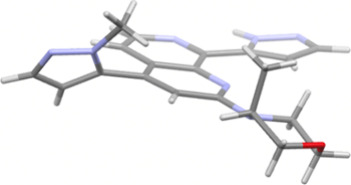
Single-crystal X-ray structure of **8b**.

Researchers in AstraZeneca recently reported interesting relationships between the number of ring atoms of *N*-aryl lactams and kinetic aqueous solubility.^33^ They found that measured Log*D* is reduced by half a log unit by the addition of methylene to the ring, and the six-membered-ring analog **9b** was 120-fold more soluble than the five-membered matched pair **9a** in phosphate buffer (Table 8). Additional analyses of in-house-matched molecular pairs revealed that several six-membered analogs of not only *N*-aryl lactams but also *N*-aryl imidazolinones and *N*-aryl oxazolinones were more soluble than the five-membered matched pairs. DFT calculation revealed that the five-membered lactams lie almost planar with the aromatic ring, whereas the six-membered lactams take a nearly orthogonal conformation due to the steric hindrance between the lactam’s carbonyl group and *ortho*-hydrogen atom. This orthogonal conformation would not only disrupt molecular planarity (reduction of packing energy) but also decrease electron delocalization (resonance), which can result in stronger HBs. Interestingly, the *ortho*-substituted six-membered ring compounds (**9c**) are more lipophilic and less soluble than the corresponding five-membered analogs (**9d**) because both types of analogs possess twisted conformation by *ortho*-substitution.

**Table 8:**
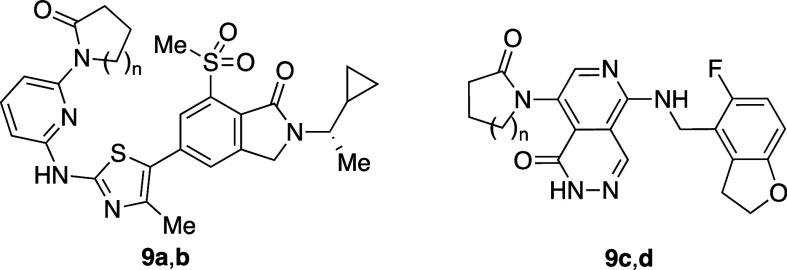
Improvement in the Kinetic Aqueous Solubility of *N*-aryl Lactam by Changing the Lactam Ring Size

compd	*n*	aqueous solubility (pH 7.4) (μM)^a^	ACD LogD	CLogP	measured Log*D*	Melting onset (°C)
**9a**	1	0.054	1.6	3.0	4.4	285
**9b**	2	6.3	2.0	3.5	3.9	243
**9c**	1	66			1.8	
**9d**	2	18			1.9	

a After 24 h, using solids obtained by evaporation of DMSO stock solutions.

The effects of the *ortho*-substitution of biaryl groups are also applicable to aryl groups possessing other flat substituents. Based on the melting points of substituted benzenes (vide infra in Section 3.8),^34^ *ortho*-isomers bearing flat substituent(s) (e.g., Ph, CO_2_H, Ac, NO_2_, CONH_2_, and NHAc) tend to exhibit the lowest melting points among their regioisomers. The observed difference in mean melting points between the *ortho*-isomers and other isomers was approximately 12 °C, which can be attributed to the increased dihedral angles resulting from the substitution pattern.

A concrete example of pharmaceutically relevant compounds exhibiting this trend is provided by *ortho*-dimethylbenzamide analogs (**10c** and **10d**) (Table 9).^35^ These analogs showed improved thermodynamic aqueous solubility compared with their monomethyl counterparts (**10a** and **10b**). The enhanced solubility was likely due to the disruption of molecular planarity, as evidenced by the over 70 °C reduction in melting points for the *ortho*-disubstituted analogs relative to their monosubstituted counterparts.

**Table 9:**
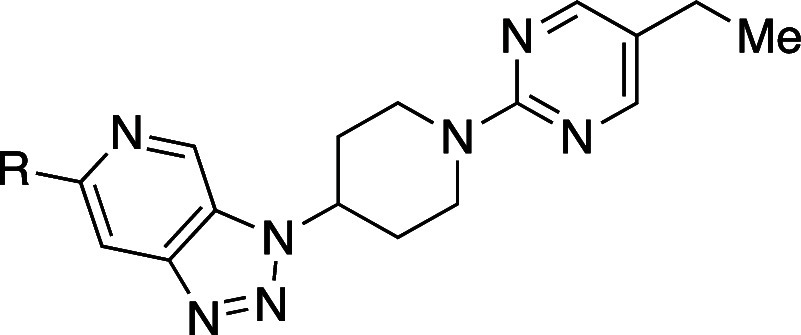

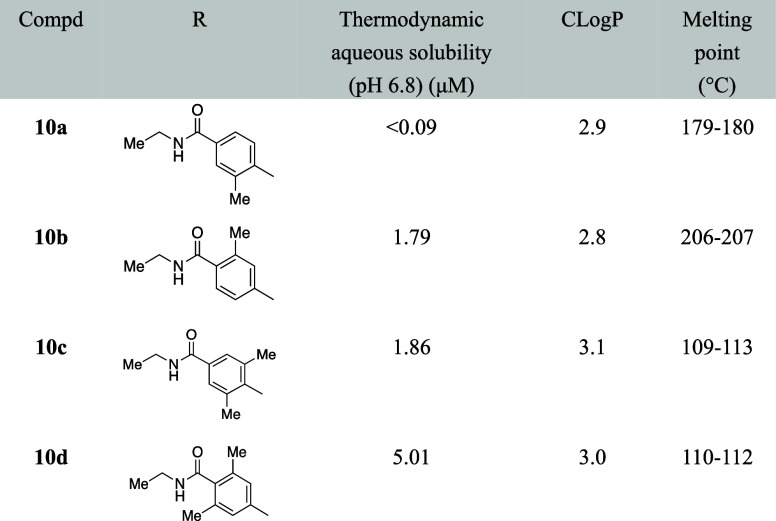
Improvement in Thermodynamic Aqueous Solubility by *ortho*-Substitution of Benzamide Group

#### 3.1.2 Disruption of Molecular Planarity by Substitution of Benzylic Positions

Researchers at AstraZeneca identified a selective *N*-methyl-d-aspartate antagonist **11a**, which faced challenges due to its poor solubility and bioavailability (Table 10).^36^ To address these issues, the α-methyl analogue **11b** was developed. The molecular planarity was disrupted in this analogue, resulting in at least a 5.8-fold improvement in aqueous solubility compared with **11a**. Similarly, α-methyl analogue **11b** had better oral bioavailability (30%) than the unsubstituted compound **11a** (5%). Mechanistically, the α-methylation resulted in a lower melting point, which in turn enhanced solubility, despite the concomitant increase in hydrophobicity.

**Table 10:**
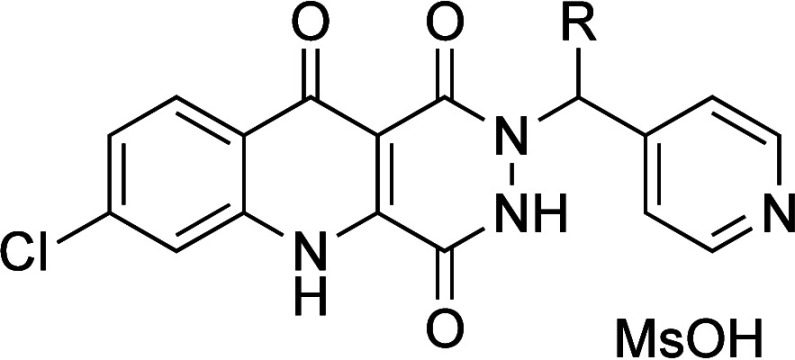
Improvement in Aqueous Solubility and Oral Bioavailability by α-Substitution

compd	*R*	aqueous solubility (pH 7.4) (mg mL^–1^)^a^	CLogP	melting point (°C)^a^	Caco-2 *P*_app_ (nm s^–1^)	Rat *F* (%)
**11a**	H	0.05	1.4	277–278	200	5
**11b**	Me	>0.29	1.8	245–247	11000	30

a Methanesulfonate salt.

Hypoxia-inducible factor prolyl hydroxylase domain inhibitor **12a** showed good *in vivo* efficacy, but its bioavailability in rats was only 16% (Table 11).^37^ This low bioavailability was attributed to its poor solubility in a pH 6.5 buffer (9.5 μg/mL). To address this issue, the authors introduced fluorine atoms onto the biphenyl moiety, hypothesizing that this modification would increase the dihedral angle. Indeed, the solubility of **12b** in pH 6.5 buffer was increased to 49 μg/mL. Subsequently, reducing the number of aromatic rings in the structure (**12c**) led to a 9-fold improvement in solubility compared with **12a**. Further optimization involved the introduction of a methyl group at the α-position to disrupt intermolecular interactions. Remarkably, this modification, affording compound **12e**, resulted in a dramatic increase of solubility to over 1000 μg/mL—more than 13-fold higher than that of **12d**. The enhanced solubility of **12e** was attributed to steric hindrance between the *ortho*-chloro atom and the α-methyl group, which caused increased torsion of the phenyl ring. This hypothesis was supported by the difference in melting points (**12d**: 262–267 °C; **12e**: 238–240 °C). Importantly, **12e** exhibited a significantly improved pharmacokinetic profile, with an oral bioavailability in rats of 77% compared with 16% for parent compound **12a**.

**Table 11:**
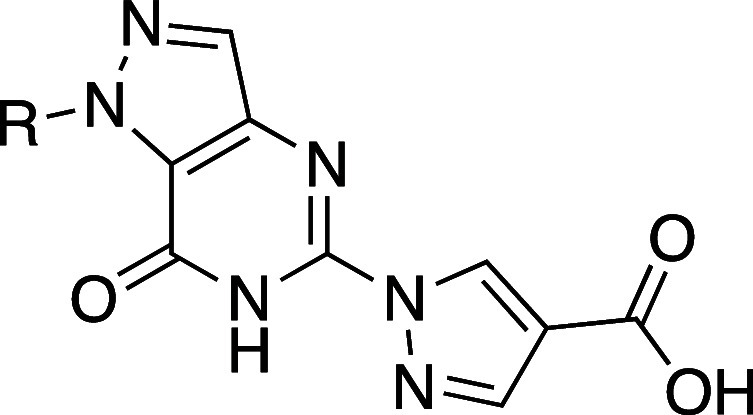

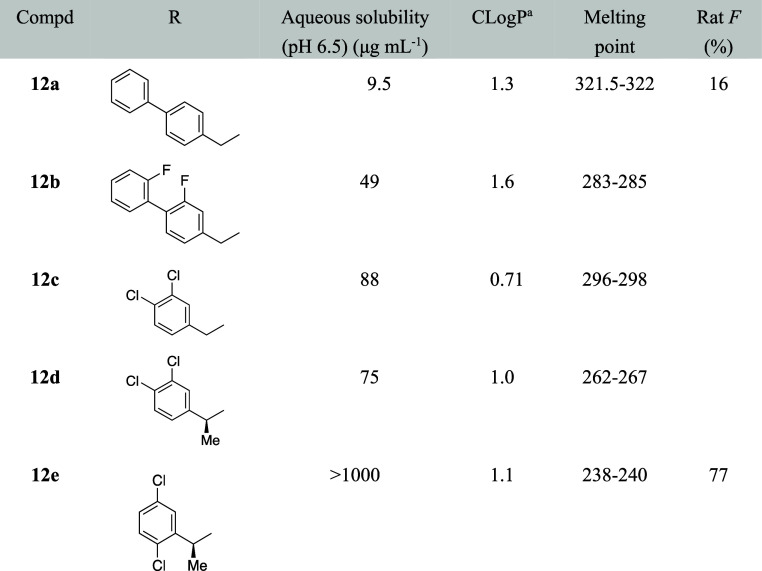
Improvement in Aqueous Solubility and Oral Bioavailability by α-Substitution

a CLogP was estimated by us, using ChemDraw Ultra 20.0.

During the course of the development of an inhibitor of mutant isocitrate dehydrogenase 1, the introduction of a methyl group (**13b** and **13c**) at the α-position of **13a** resulted in 12.5- and 8.9-fold improvement of solubility, respectively (Table 12).^38,39^ This enhancement was likely due to the disruption of molecular planarity caused by the α-methyl group despite the increase in hydrophobicity. Additionally, the pyrimidyl analog **13d** showed a 16.4-fold improvement in solubility compared with **13a**, with an oral bioavailability (*F*) of 81% in rats. A subsequent study achieved an even more pronounced effect: the introduction of a methyl group at the α-position of **13e** to produce **13f** led to a remarkable 116-fold improvement in aqueous solubility, again despite increased hydrophobicity.^39^

**Table 12:**
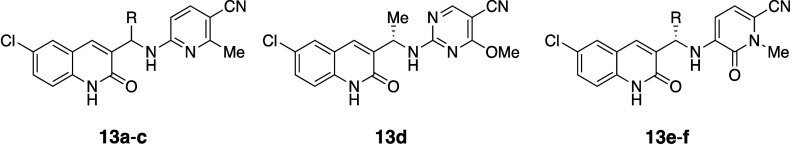
Improvement in Aqueous Solubility and Oral Bioavailability by α-Substitution

compd	*R*	kinetic aqueous solubility (pH 7.4) (μM)	CLogP^a^	rat *F* (%)
**13a**	H	0.64	1.0	
**13b**	(*R*)-Me	8.0	1.4	
**13c**	(*S*)-Me	5.7	1.4	
**13d**		10.5	–0.46	81
**13e**	H	0.295	–0.32	
**13f**	Me	34.5	–0.014	

a CLogP was estimated by us, using ChemDraw Ultra 20.0.

### 3.2 Case Studies on Decreasing the Flatness of Chemical Structures

Aromatic rings are ubiquitous in bioactive molecules, with the phenyl ring being a component of approximately 45% of marketed small-molecular drugs.^40^ The high planarity of the phenyl moiety promotes molecular stacking, contributing to reduced aqueous solubility and increased metabolic susceptibility of drug molecules.^41^ Introducing sp^3^ carbons into the benzene ring is an effective strategy to disrupt this planarity.

#### 3.2.1 Saturation (Escape from Flatland)

A large-scale data analysis focusing on the ratio of sp^3^-hybridized carbons (Fsp^3^) indicated a positive correlation between Fsp^3^ and success in drug development.^20^ In another data set of more than 1000 compounds with simple molecular structures, Fsp^3^ correlated positively with solubility and negatively with melting point. Furthermore, a recent study suggested that increasing Fsp^3^ reduces promiscuity and CYP450 inhibition.^42^ Researchers at GSK analyzed a data set of 100,000 GSK compounds and proposed the Property Forecast Index (PFI), calculated as the sum of the chromatographic Log*D*_7.4_ value and the aromatic ring count (Chrom Log*D*_7.4_ + #Ar).^41^ A PFI value of less than 5 was predictive of a higher probability of achieving kinetic solubility (>200 μM).

As a concrete example of matched molecular pair analysis, vanilloid receptor-1 antagonist **14a** (Table 13) showed insufficient thermodynamic aqueous solubility (<1 μg/mL in PBS or 0.01 M HCl).^43^ To improve its aqueous solubility, a strategy involving partial saturation of the 4-(trifluoromethyl)­phenyl ring was employed to reduce structural planarity, disrupt π-π stacking, and disrupt crystal packing. The partially saturated analogue **14b** exhibited improved thermodynamic solubility (13 μg/mL in 0.01 M HCl), being at least 13-fold more soluble than **14a**. The lower melting point of **14b** compared with **14a** supports the hypothesis that disruption of planarity contributed to its improved solubility.

**Table 13:**
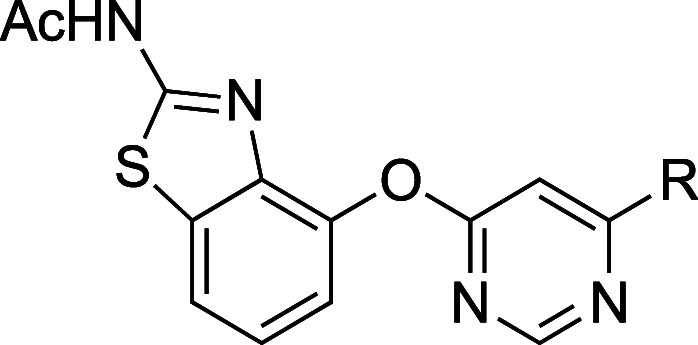

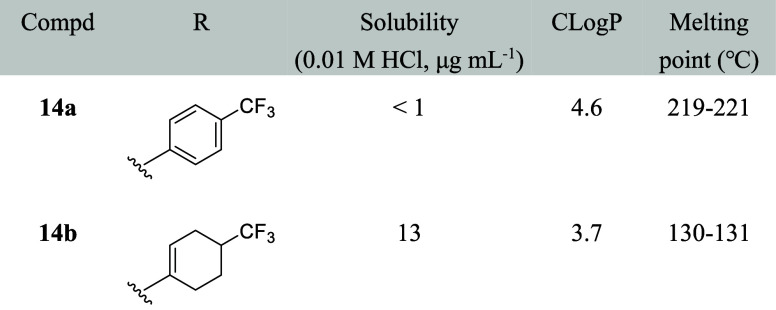
Improvement in Aqueous Solubility by Saturation of Aromatic Ring

Compound **15a** showed poor pharmacokinetics and low solubility, which precluded further development (Table 14).^44^ The authors hypothesized that the removal of two aromatic rings, that is, the oxadiazole ring and the phenyl ring of the benzoic acid moiety, would enhance the three-dimensional morphology of the compound, the aim being to weaken the crystal packing of **15a** and thereby improve the dissolution rate. Indeed, *trans*-cyclohexyl analog **15b** showed 400-fold improved aqueous solubility, greater PAMPA permeability, and increased oral bioavailability in rats. In a phase 1 clinical trial, **15b** was well absorbed in both normal healthy volunteers and patients, with both the AUC and Cmax exhibiting dose-proportionality following single oral doses in the range from 0.5 to 25 mg in the fed state.^11^

**Table 14:**
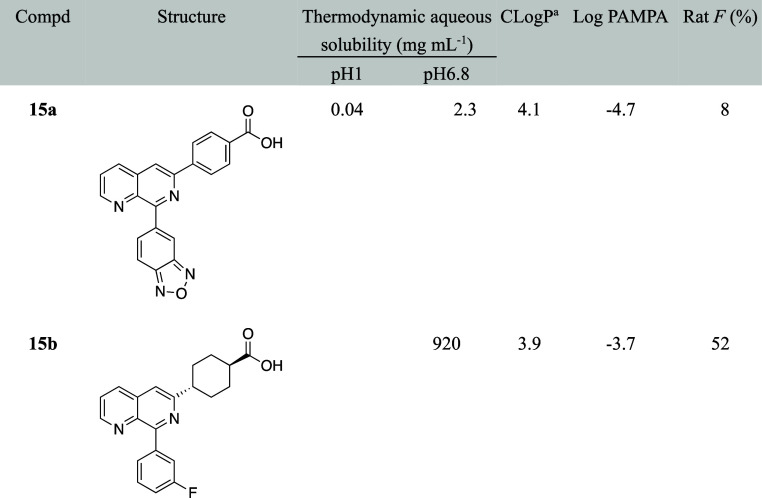
Improvement in Thermodynamic Aqueous Solubility and Oral Bioavailability by Saturation/deletion of Aromatic Ring

a CLogP was estimated by us, using ChemDraw Ultra 20.0.

Fairhurst et al. reported that 2-formylpyridinyl ureas act as reversible covalent inhibitors of fibroblast growth factor receptor 4. Chemical modification involving substitution of the phenyl moiety in compound **16a** with piperidine (**16b**) resulted in a lower melting point (133 °C) compared to that of **16a** (175 °C). This modification also improved thermodynamic water solubility, with aqueous solubilities of <2.9 μM for **16a** and 4.9 μM for **16b** at pH 6.8 (Table 15).^45^

**Table 15:**
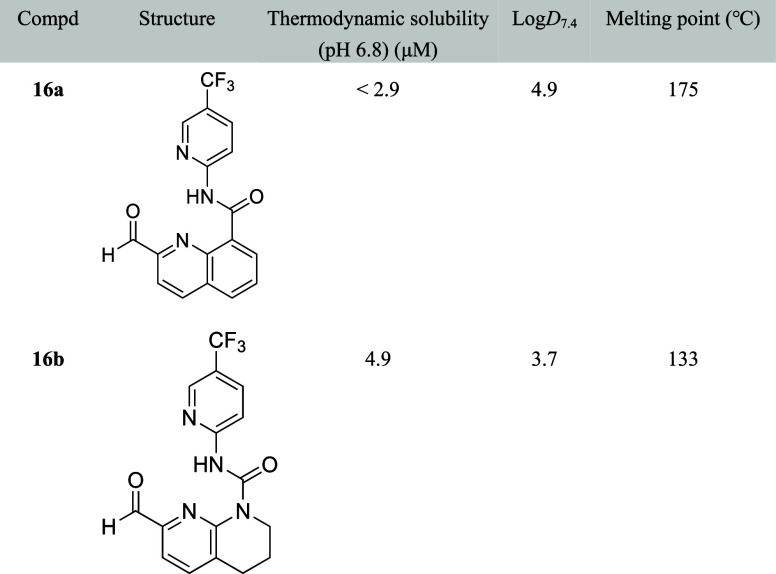
Improvement in Aqueous Solubility by Increase of Saturation

#### 3.2.2 Phenyl Ring Mimetics (Aufheben of Phenyl Spacer and Druglikeness)

Several phenyl bioisosteres containing sp^3^ carbons have been reported. When a benzene ring substituted at the para position is replaced, the distance between the substitution sites becomes crucial. The positional relationships and surface area of various phenyl bioisosteres are summarized in Table 16.^46^

**Table 16:**
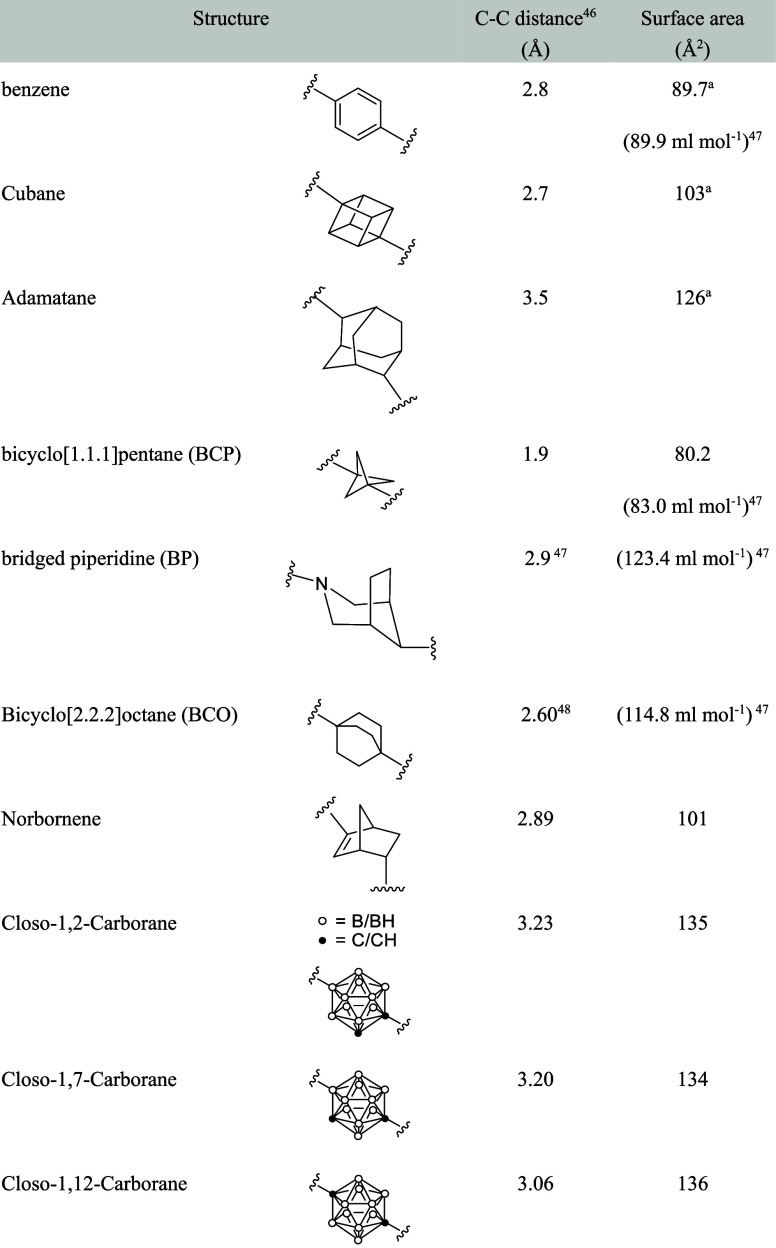
Structural Information of *para*-Substituted Phenyl Bioisosteres

a Surface area and volume were calculated using a model of a solvent-excluded molecular surface of a compound with hydrogen atoms substituted on both sides. C–C distance is the distance between the substitution sites.

Cubane, being approximately the same size as a phenyl group, has long been recognized as being of interest in the field of phenyl bioisosteres.^49^ The distance along the body diagonal of cubane (2.68 Å) closely matches that of benzene (2.77 Å) (Table 16), despite slightly longer individual C–C bond lengths (1.573 Å (sp^3^) vs 1.397 Å (sp^2^)).^50^ A pioneering report on cubanes as bioisosteres of a *para*-phenyl group appeared in 2016.^51^ However, with regard to druglikeness, only the Log*P* values were used to predict the water solubility and membrane permeability of cubane analogs. The improvement of Log*P* values resulting from substitution of the phenyl group is shown in Table 17.

**Table 17:**
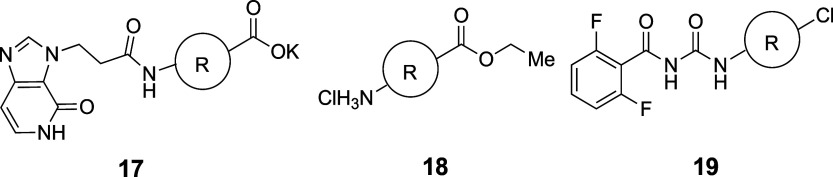

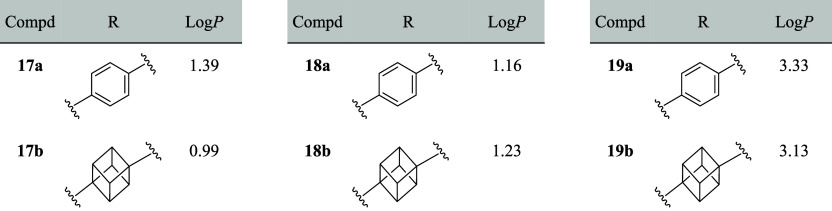
Effect of Replacement of a Phenyl Group with Cubane on Hydrophobicity

In a study of bioisosteres of monosubstituted phenyl, Todd et al. synthesized compounds in which the phenyl group of the antimalarial lead compound **20a** was substituted with cubane **20b**, bicyclo[1.1.1]­pentane (BCP) **20c**, and closo-1,2-carborane **20d** (Table 18).^46^ Both cubane **20b** and closo-1,2-carborane (**20d**) exhibited higher melting points and lower aqueous solubility than **20a**, suggesting that cubane- and carborane-substitutions do not always improve solubility. In addition, **20b** and **20d** showed high clearance and short half-lives in studies with human liver microsomes (HLMs) and mouse liver microsomes (MLMs). In the case of BCP derivative **20c**, there was no significant change in lipophilicity or water solubility, and the melting point was higher than that of **20a**.

**Table 18:**
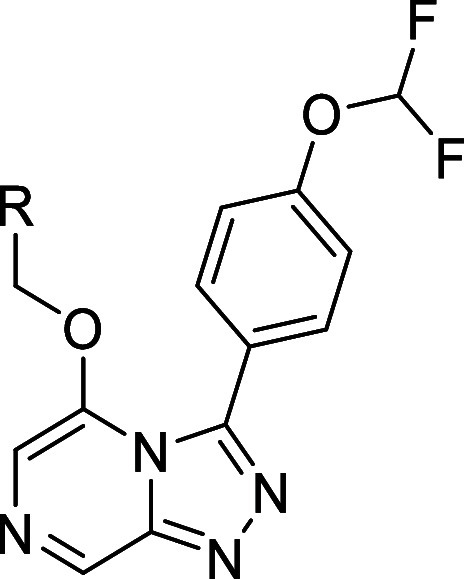

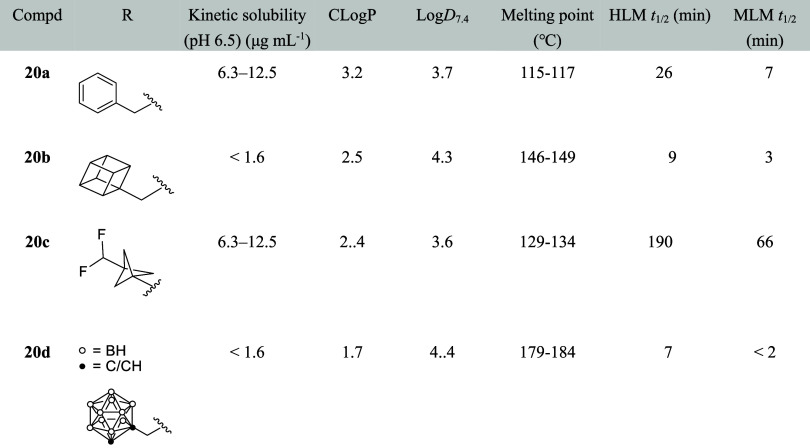
Effect of the Bioisosteric Replacement of the Terminal Phenyl Ring

Auberson et al. compared the water solubility of relatively simple model structures in which the benzene ring (**21a**, **22a**) was replaced with BCP (**21b**, **22b**), bicyclo[2.2.2]­octane (BCO) (**21c**, **22c**), or cubane (**21d**, **22d**) (Table 19).^52^ The results indicate that BCP affords superior water solubility compared with BCO, cubane, and phenyl in these cases.

**Table 19:**
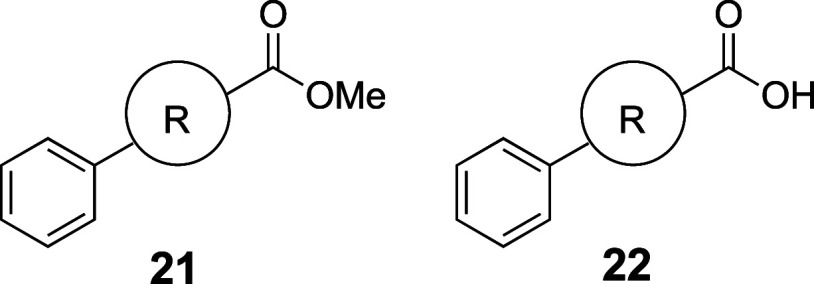

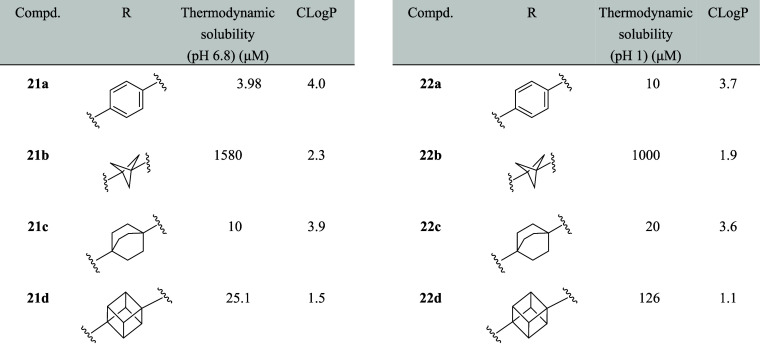
Improvement of Thermodynamic Aqueous Solubility by Substitution of a Phenyl Group with Phenyl Isosteres

Stepan et al. developed a γ-secretase inhibitor **23b** by replacing the *para*-substituted fluorophenyl ring of BMS-708,163 (**23a**) with a BCP motif.^53^ Compared with fluorophenyl analog **23a**, BCP analog **23b** showed 11.6-fold and 32.7-fold improvement in thermodynamic aqueous solubility at pH 6.5 and pH 7.4, respectively. According to our mechanistic analysis, the density of a single crystal of **23b** (1.468 g/cm^3^) was lower than that of **23a** (1.528 g/cm^3^) suggesting that “escape from flatland” at least partially contributed to the solubility improvement. Another mechanism contributing to the solubility improvement would be the decrease in Log*D*_7.4_ (4.70 for **23a**, 3.80 for **23b**). Reduction of Log*D* would also reduce the clearance in human hepatocytes (CL_int,app_), that is, the clearance of **23b** was lower than that of **23a**. BCP analogue **23b** also showed a significant improvement in permeability (5.52 × 10^–6^ cm/s for **23a**, 19.3 × 10^–6^ cm/s for **23b**). Compound **23b** showed excellent oral bioavailability in rats (100%), as well as sufficient brain partitioning (total brain/total plasma = 0.61) (Table 20).

**Table 20:**
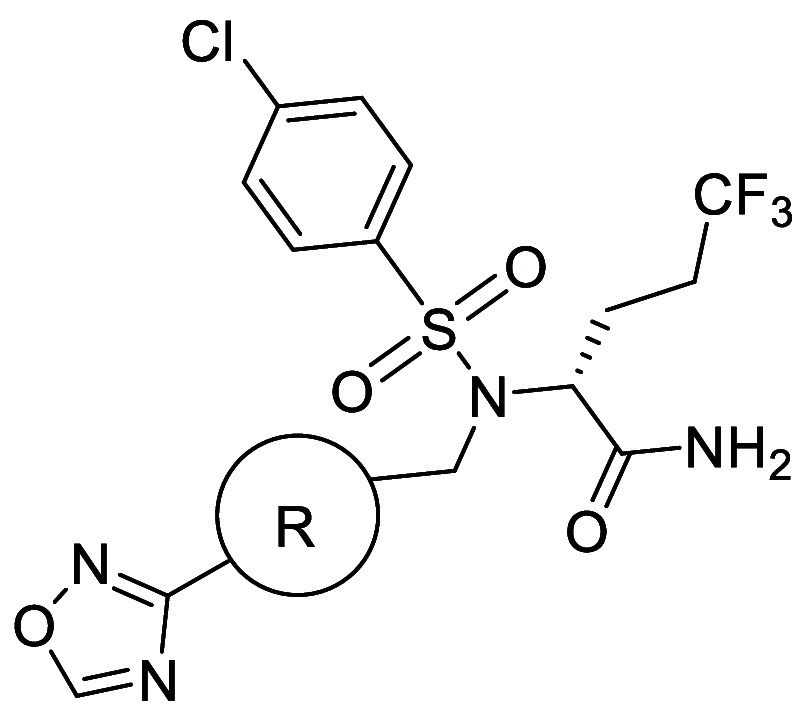

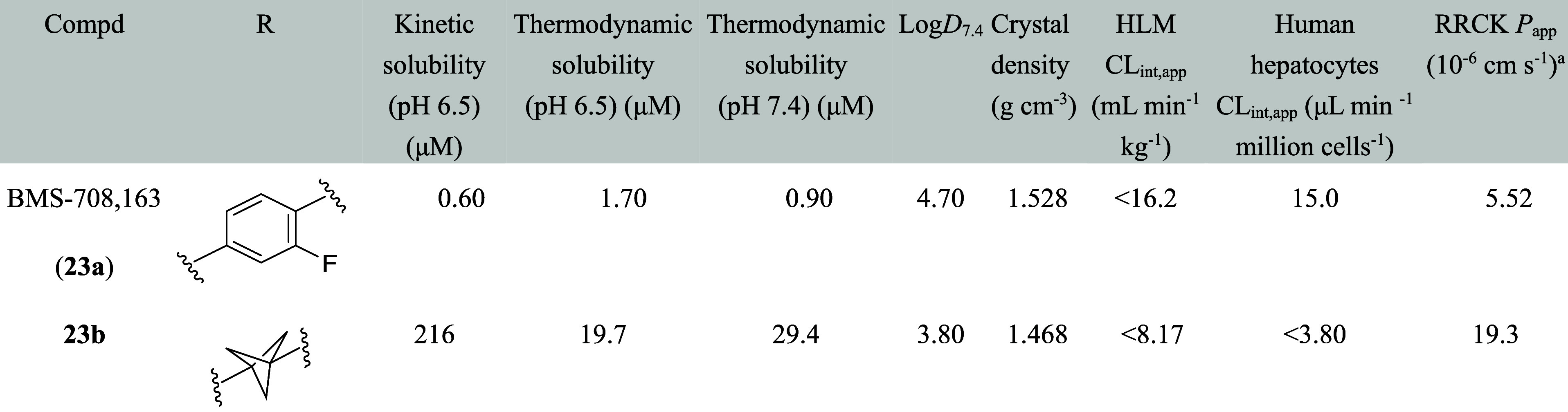
Improvement in Thermodynamic Aqueous Solubility and Permeability by Bioisosteric Replacement of the Benzene Ring with BCP

a RRCK (Ralph Russ canine kidney) cells with low transporter activity were isolated from MDCK (Madine–Darby canine kidney) cells.

The same group also reported BCP substitution of the benzene ring in imatinib (**24a**), a potent inhibitor of ABL1 kinase, affording **24c** (Table 21).^54^ BCP analogue **24c** exhibited an over 80-fold improvement in aqueous solubility (2506 μM at pH 7.4) compared to **24a** (Table 21). There was a significant decrease in melting point (**24a**: 204 °C vs **24c**: 195–198 °C), suggesting that a decrease in intermolecular interactions contributed at least partially to the solubility improvement, in addition to the decrease in lipophilicity (**24a**: Log*D*_7.4_ = 2.45 vs **24c**: Log*D*_7.4_ = 1.51).

**Table 21:**
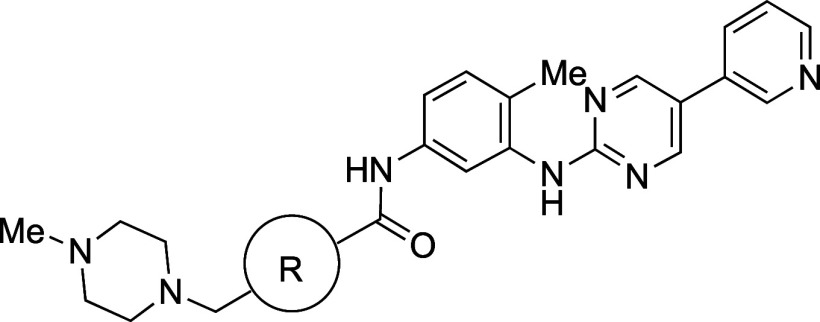

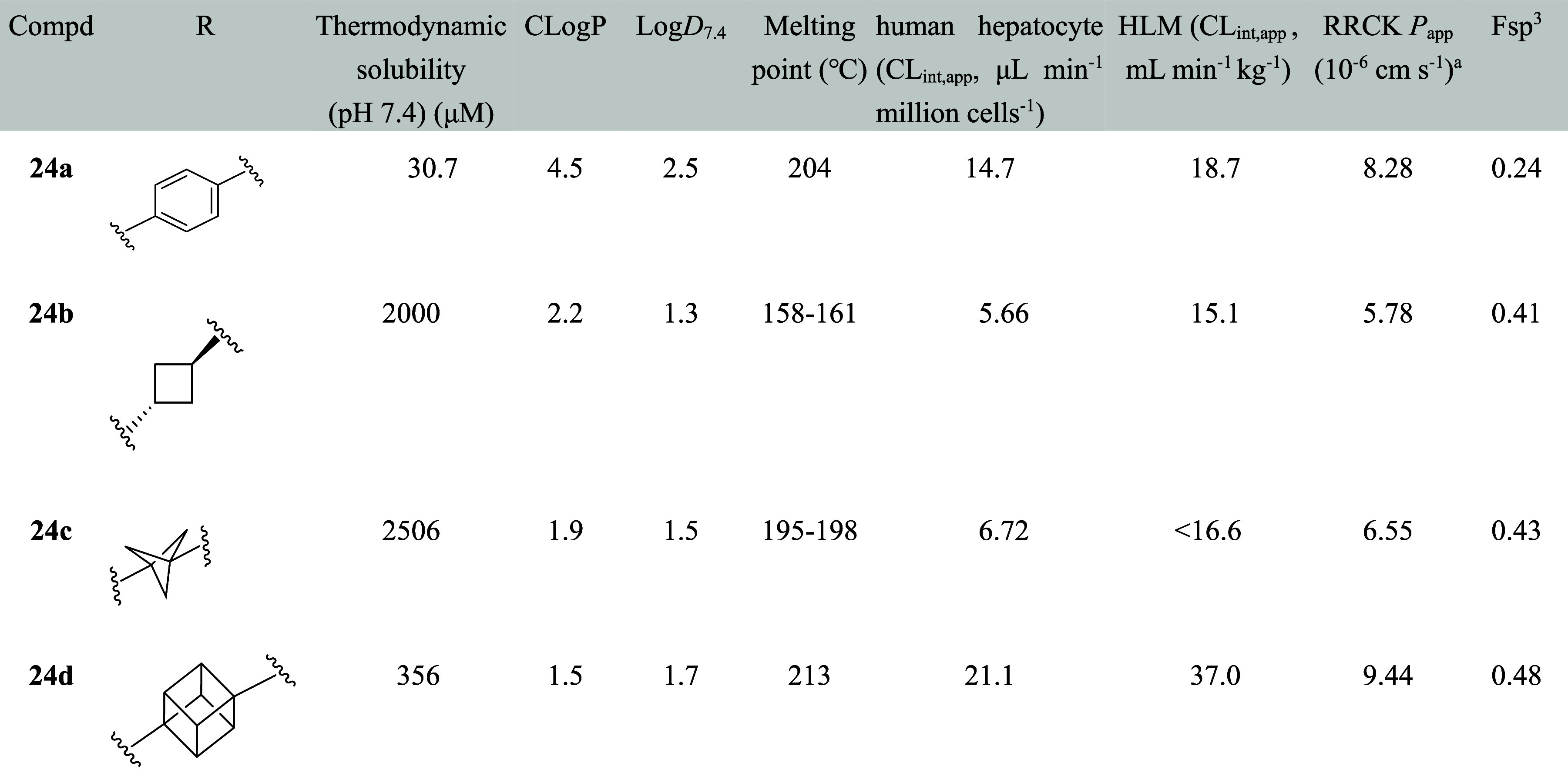
Improvement in Thermodynamic Aqueous Solubility by Bioisosteric Replacement of the Benzene Ring with BCP

a RRCK cells with low transporter activity were isolated from MDCK cells.

Hirst et al. successfully incorporated the BCP moiety into known lipoprotein-associated phospholipase A2 inhibitors as a bioisosteric phenyl substituent. The BCP-containing compound **25b** exhibited a 9-fold increase in kinetic solubility (74 μM) compared to compound **25a** (8 μM) (Table 22).^55^ Furthermore, the thermodynamic solubility in FaSSIF was determined, with analogue **25b** displaying an approximately 3-fold improvement (>1000 μg/mL versus 399 μg/mL for compound **25a**). This increase in solubility was accompanied by greater lipophilicity, as indicated by the measured Log*D*_7.4_, which increased from 6.3 to 7.0. Analog **25b** showed significantly enhanced permeability of 705 nm/s compared to 230 nm/s for compound **25a**. Additionally, low clearance was observed for **25b** in a HLM assay (1.22 mL/min/g). The authors concluded that these data lend weight to the hypothesis that disrupting molecular planarity and reducing the aromatic ring count can increase solubility and improve the overall pharmacokinetic profile.

**Table 22:**
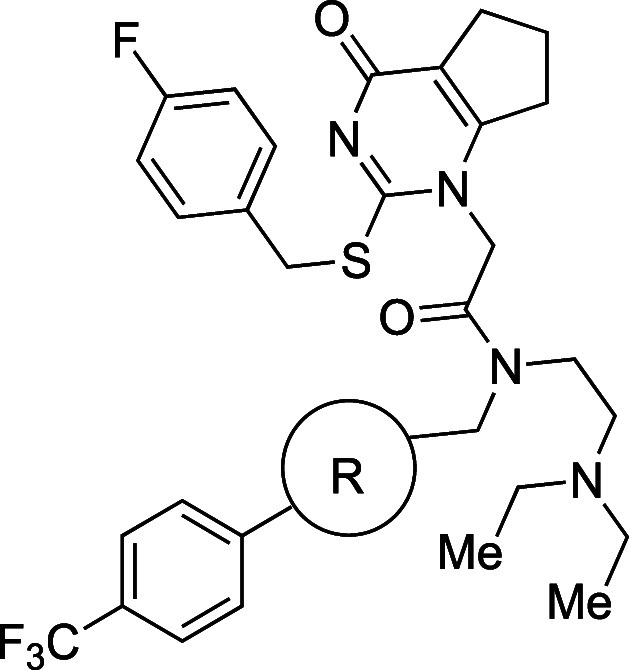

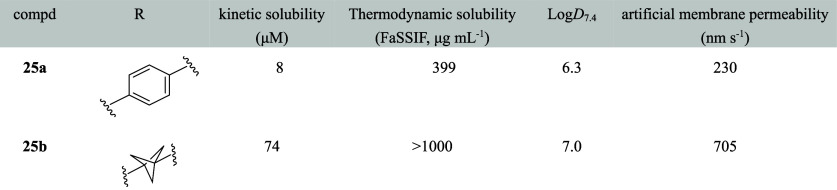
Improvement in Thermodynamic Aqueous Solubility and Permeability by Bioisosteric Replacement of the Benzene Ring with BCP

Adsool et al. developed BCP-resveratrol by replacing the phenyl group of resveratrol (**26a**) with BCP to alleviate the pharmacokinetic issues associated with resveratrol itself (Table 23).^56^ BCP-resveratrol **26b** showed 33-fold higher thermodynamic solubility than **26a**. Furthermore, **26b** also showed higher *C*_max_ and AUC values than **26a**, indicating a favorable *in vivo* PK profile.

**Table 23:**
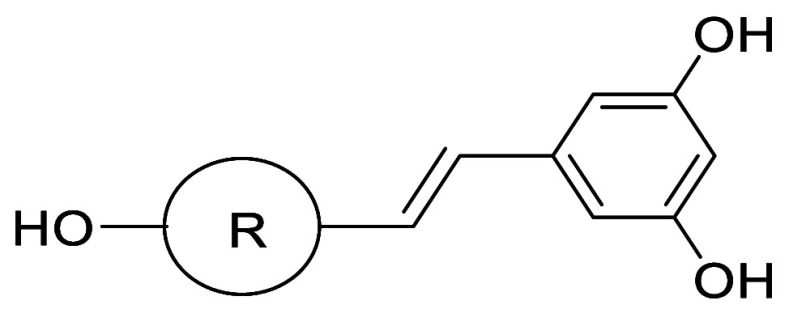

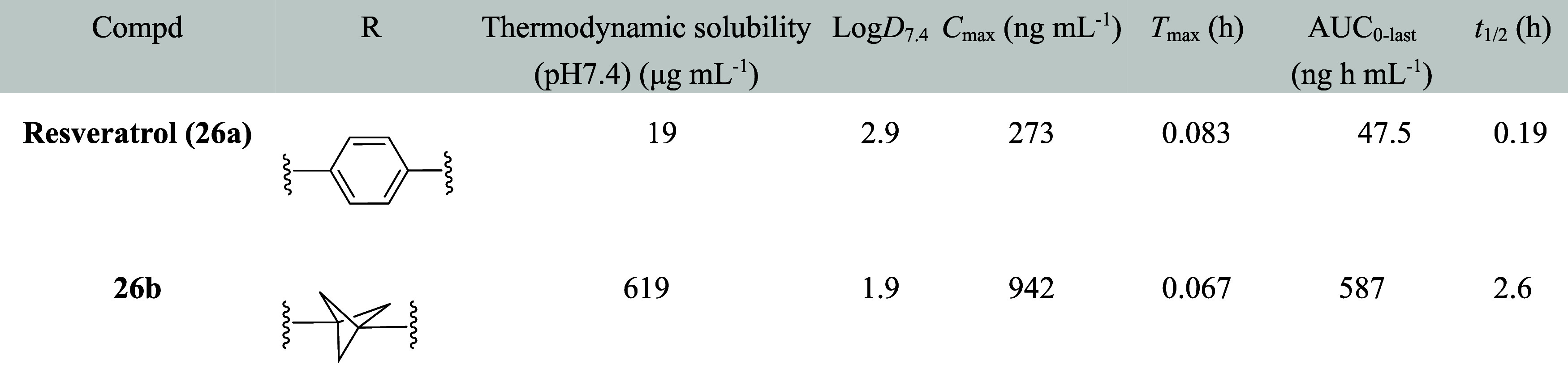
Improvement in Thermodynamic Aqueous Solubility and Pharmacokinetics by Bioisosteric Replacement of the Benzene Ring of Resveratrol

Attempts were also made to further enhance the physicochemical properties of **26b** through modifications of the BCP structure. Mykhailiuk et al. synthesized difluoro-substituted BCP **27c**. Compared to the compound with a benzene ring (**27a**), **27c** exhibited a similar Log*D* but showed higher water solubility (Table 24). However, **27c** showed lower water solubility than BCP **27b**.^57^

**Table 24:**
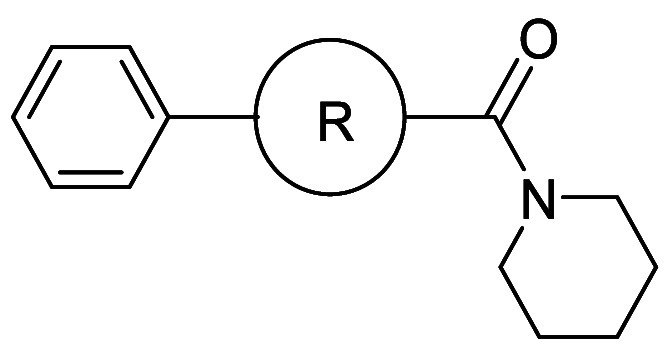

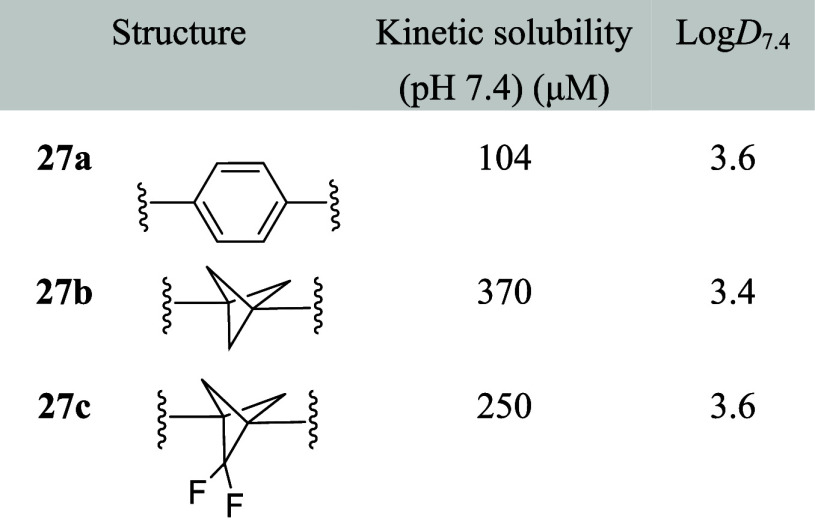
Improvement in Aqueous Solubility by Substitution of Benzene Ring with Difluoro-Substituted BCP

Ratni et al. propose the use of a bridged piperidine (BP) moiety as a phenyl bioisostere. The bond angle of BP is 170°, deviating slightly from the value of 180° seen in 1,4-phenyl, BCP, and BCO, but the distance between substituent positions is 2.9 Å, which is similar to that of 1,4-phenyl (2.8 Å) rather than BCP’s 1.9 Å (Figure 5). To test this alternative scaffold, they compared the activity and physicochemical properties of BP, phenyl, BCP, and BCO derivatives of four sets of pharmaceutical compounds including a clinical candidate.^47,48^ In all four case studies, BP analogs showed the highest aqueous solubility, while phenyl analogs showed the lowest solubility. Log*D* values of BP analogs were around 0.5 lower than those of the corresponding phenyl analogs. Thus, BP appears to be an excellent alternative to phenyl, markedly improving druglike properties such as solubility. The physicochemical properties of representative examples of phenyl isosteres, including BP, are shown in Table 25.

**Figure 5:**
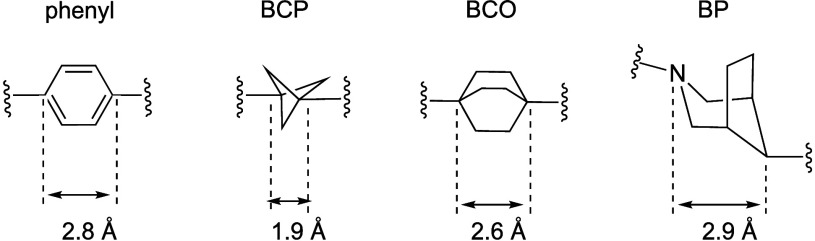
Distance between substituent positions of phenyl, BCP, BCO, and BP.

**Table 25:**
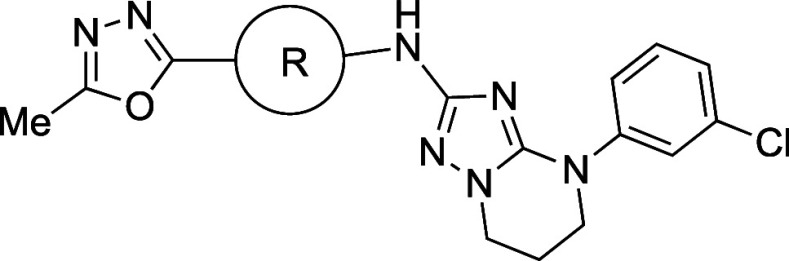

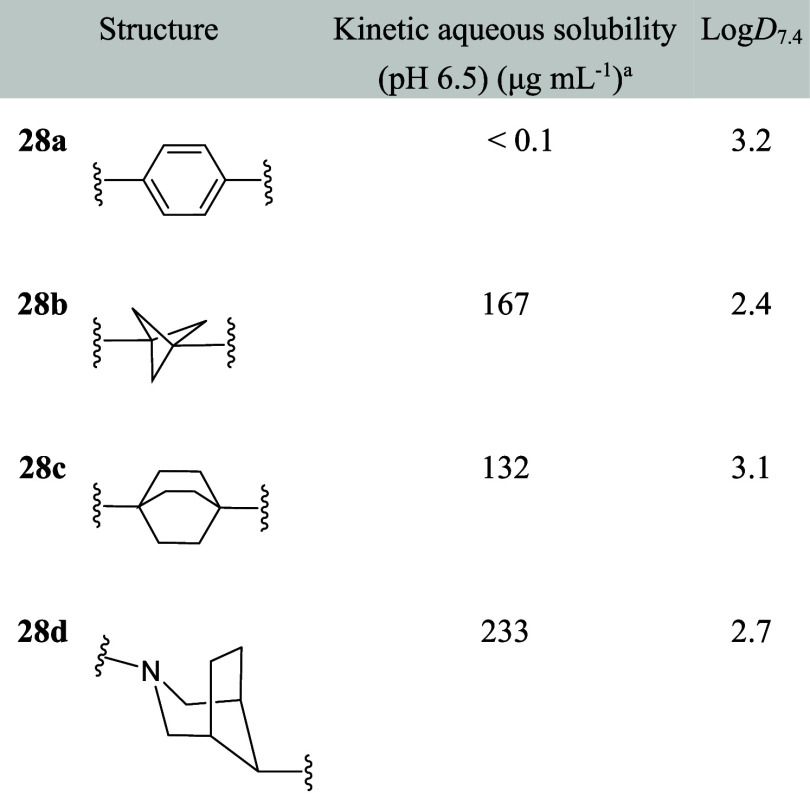
Improvement in Aqueous Solubility by Substitution with Phenyl Isosteres, BCP, BCO, and BP

a LYSA (lyophilization solubility assay) assay (after evaporation of DMSO solution, the compounds were dissolved in phosphate buffer (pH 6.5) overnight).

Zhong et al. synthesized compounds by replacing a portion of the biphenyl structure of an HCV NS5A inhibitor with a cyclohexylphenyl or bicyclo­[2,2,2]­octylphenyl motif. Compounds such as cyclohexylphenyl analog **29b** and BCO analog **29c** exhibited favorable oral bioavailability in rats, and their liver/plasma concentration ratios were also satisfactory (Table 26).^58^

**Table 26:**
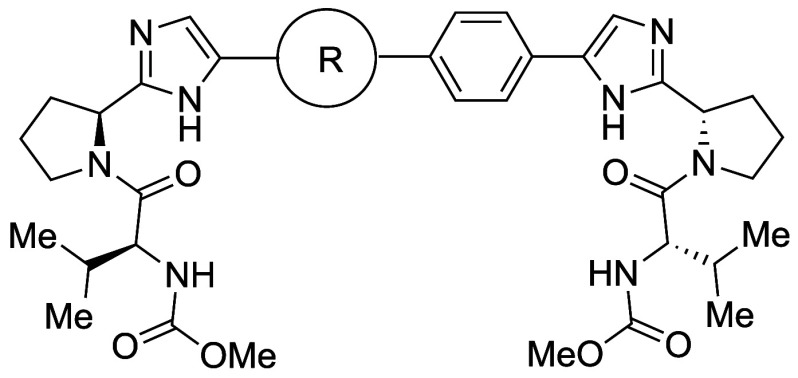

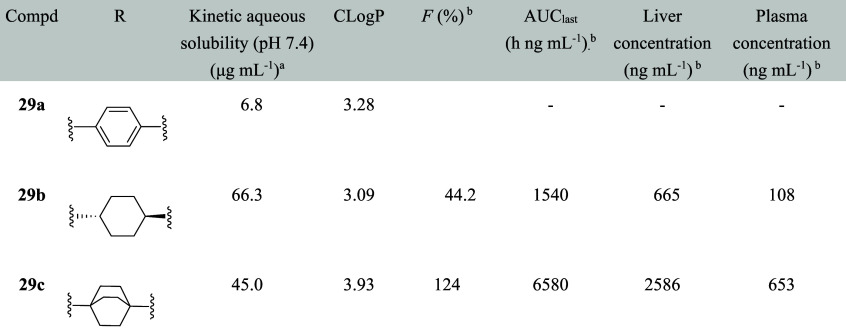
SAR on the Bridge of HCV NS5A Inhibitors

a The solubility data were collected by using μSOL Explorer with UV detection at 254 nm. ^b^. 5 mg/kg, po administration in rats.

There have also been reports on bioisosteres of *meta*- and *ortho*-disubstituted phenyl groups, such as oxabicyclo[2.1.1]­hexanes, bicyclo[3.1.1]­heptanes (BCHep), 3-azabicyclo[3.1.1]­heptanes, and [2.2.0]­bicyclohexanes. Table 27 summarizes the bond angles and bond lengths of the bioisosteres of *para*- and *meta*-disubstituted phenyl and *meta*-phenyl structures along with BCP.

**Table 27:**
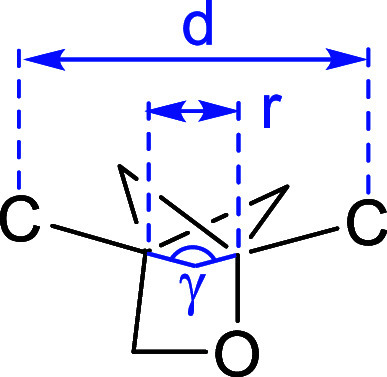

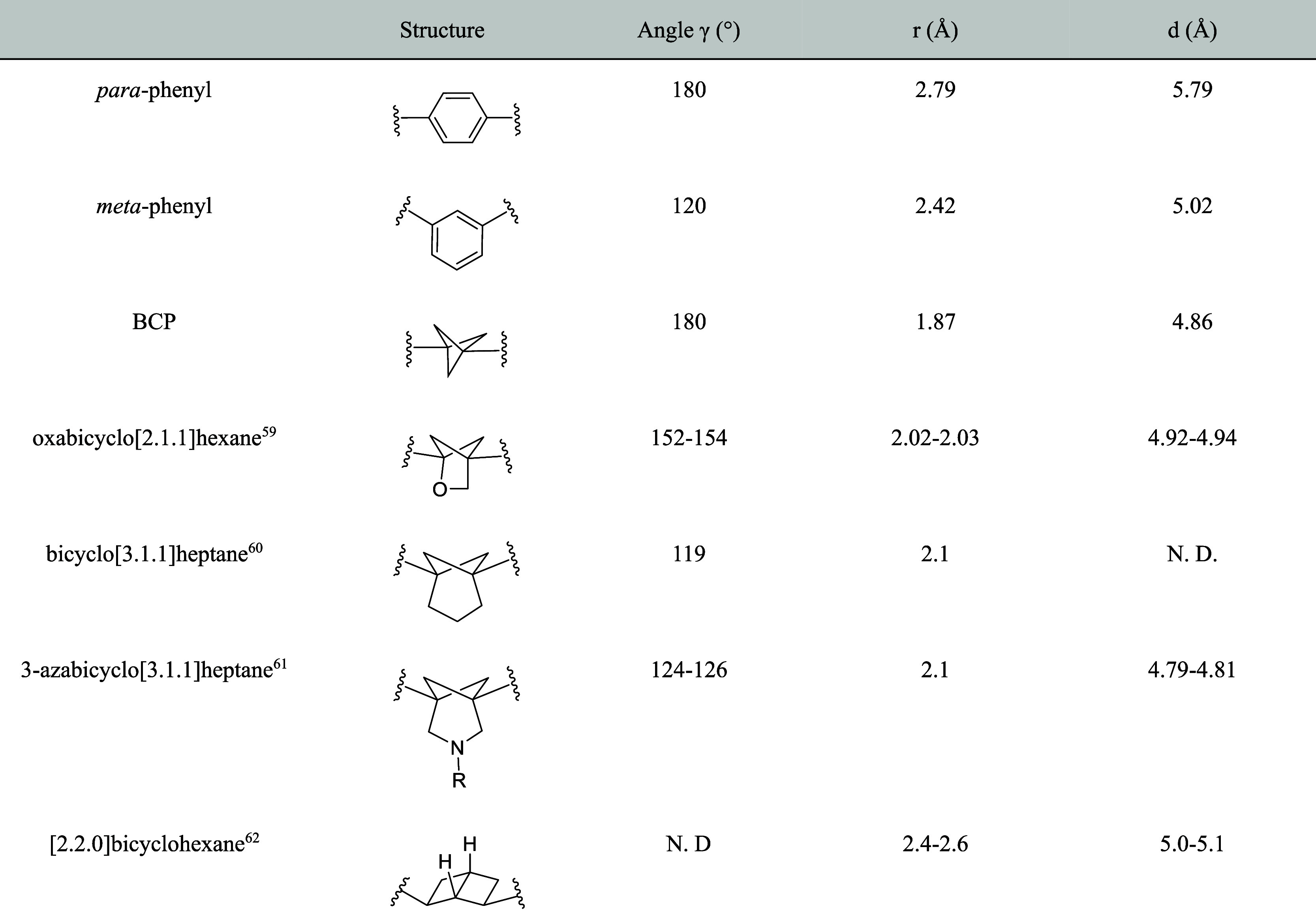
Structural Properties of Bioisosteres of *meta*-Disubstituted Phenyl Groups

Mykhailiuk et al. developed oxabicyclo[2.1.1]­hexanes as analogs of BCP.^59^ Adding an oxygen atom to BCP (**30c** and **30d**) results in a slightly different geometry but still provides better kinetic solubility than that of BCP analog **30b** (Table 28). The distance between the substituted positions was slightly longer than that of BCP but slightly shorter than that of *meta*-disubstituted benzene (Table 27). The angle γ, representing the deviation between the exit vectors in oxabicyclo[2.1.1]­hexanes (152–154°), falls between the values for *para*-disubstituted phenyl (180°) and *meta*-disubstituted benzene (120°). This suggests that oxabicyclo[2.1.1]­hexanes are promising bioisosteres not only for *para*-substituted phenyl, but also for *meta*-substituted benzene, and represent a useful tool for improving physicochemical properties.

**Table 28:**
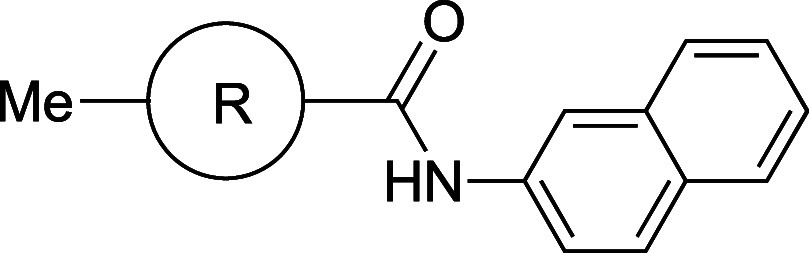

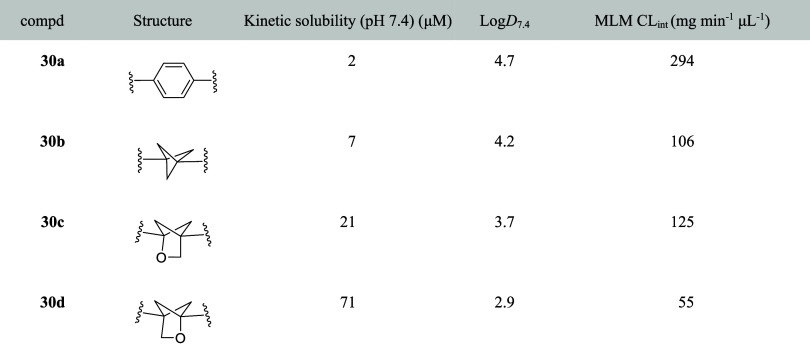
Oxabicyclo­[2.1.1]­hexanes as *para*-Phenyl and *meta*-Phenyl Isosteres

Anderson^63^ and Uchiyama^60^ independently developed practical syntheses of disubstituted BCHep as *meta*-substituted benzene mimetics. The internal C–C distance between the substituted positions was slightly shorter (2.1 Å) than that of *meta*-disubstituted benzene (2.4 Å). The angle γ, representing the deviation between the exit vectors in BCHep (119°), is almost the same as that of the *meta*-disubstituted benzenes (120°). BCHep analogs of sonidegib (**31b**) and URB597 (**32b**) showed remarkably similar physicochemical properties including CLogP, tPSA, and aqueous solubility (Table 29). Interestingly, the permeability of BCHep-substituted **31b** in Caco-2 cells was improved. These results indicated that the BCHep scaffold is suitable as a *meta*-benzene isostere.

**Table 29:**
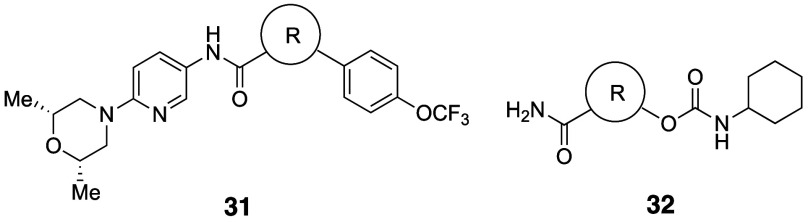

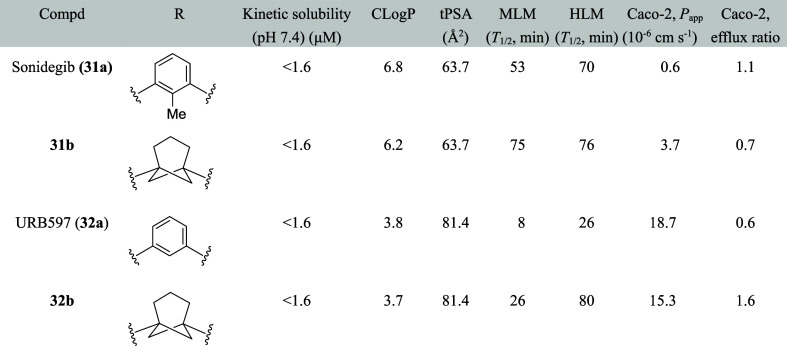
BCHep as a *meta*-Phenyl Isostere

Mykhailiuk et al. reported 3-azabicyclo[3.1.1]­heptanes as *meta*-substituted pyridine isosteres (Table 30).^61^ The internal C–C distance between the substituted positions (2.1 Å) and the angle γ (124–126°) representing the deviation between the exit vectors in 3-azabicyclo[3.1.1]­heptanes were similar to those of substituted pyridine (2.4 Å and 125°, respectively). Azabicyclo[3.1.1]­heptane analog **33b** showed 12.6-fold improved kinetic solubility and a decrease of Log*D* compared to the pyridine analog rupatadine (**33a**).

**Table 30:**
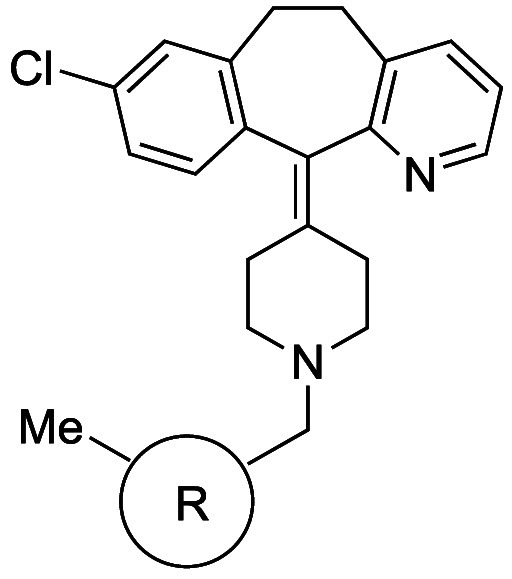

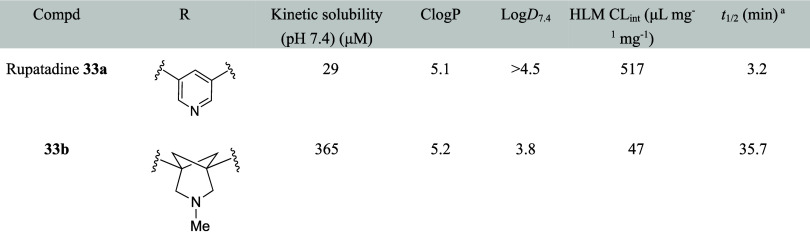
Improvement in Aqueous Solubility by Substitution with *meta*-Pyridine Isostere 3-Azabicyclo[3.1.1]­heptane

Fessard and Brown reported [2.2.0]­bicyclohexanes ([2]-ladderanes) as *meta*-substituted benzene isosteres.^62^ The internal C–C distance between the substituted positions was slightly longer (2.6 Å) than that of the *meta*-disubstituted benzene (2.4 Å). Based on matched molecular pair analysis of simple compounds (**34a** vs **34b** and **35a** vs **35b**), the authors concluded that the introduction of [2.2.0]­bicyclohexane analogs does not significantly impact physicochemical properties, including Log*P*, aqueous solubility, and permeability (Table 31).

**Table 31:**
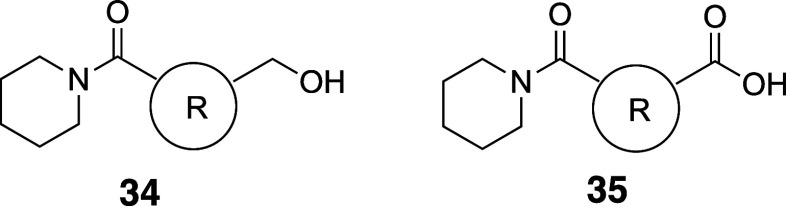

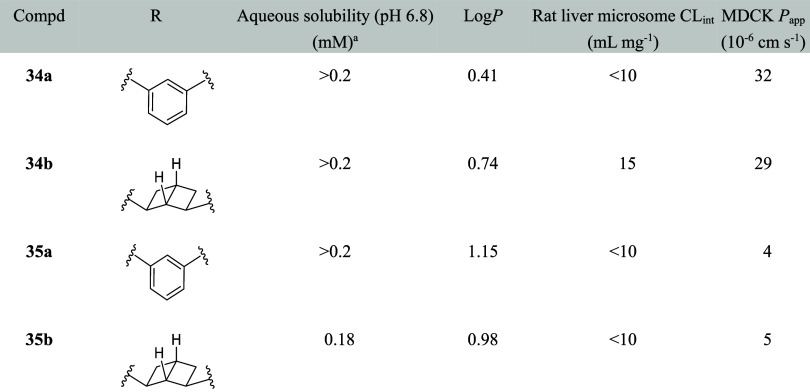
[2.2.0]­Bicyclohexane as a *meta*-Phenyl Isostere

a LYSA assay (after evaporation of their DMSO solution, the compounds were dissolved in phosphate buffer (pH 6.5) for 16 h).

There are also reports of *ortho*-disubstituted phenyl bioisosteres. Myknailiuk et al. reported bicyclo­[2,1,1]­hexanes as bioisosteres of *ortho*-disubstituted benzenes consisting of sp^3^ carbons (Table 32).^64^ Despite the difference in planarity (angle θ) between the bicyclo­[2,1,1]­hexane core (3D) and *ortho*-substituted phenyl (2D) (angle θ: bicyclo­[2,1,1]­hexanes = 44.9–78.0° vs *ortho*-substituted phenyl = 7.5–8.3°), they share similarities in other characteristics. The distance between the substituents (d) in the *ortho*-benzene of telmisartan (**36a**) is 3.10 Å, and the *d* value in the corresponding bicyclo­[2,1,1]­hexanes core **36b** is estimated to be close to that value, 3.22 Å. They also found that the bicyclo­[2,1,1]­hexane-type model compound **37b** had 1.2-fold higher water solubility while showing slightly higher Log*D* than the corresponding *ortho*-disubstituted phenyl compound **37a** (Table 33).

**Table 32:**
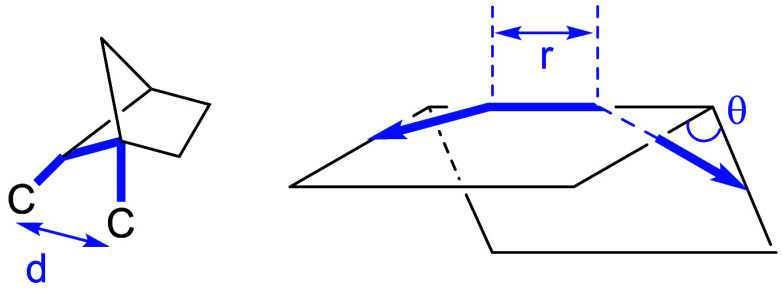

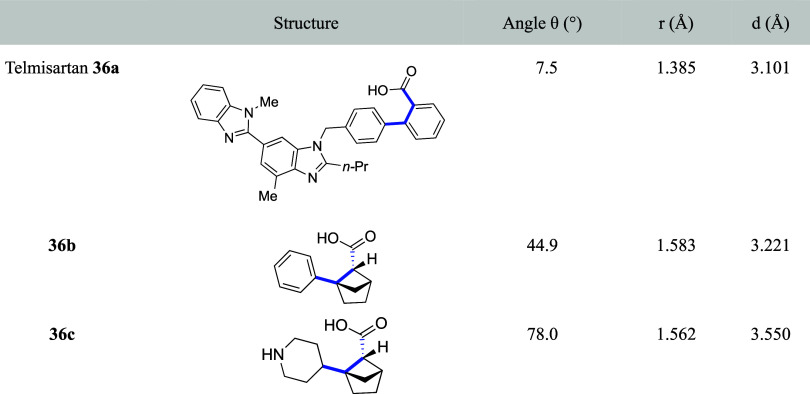
Difference of Angle and Inter-substituent Distance between *ortho*-Substituted Phenyl and Bicyclo­[2,1,1]­hexanes

**Table 33:**
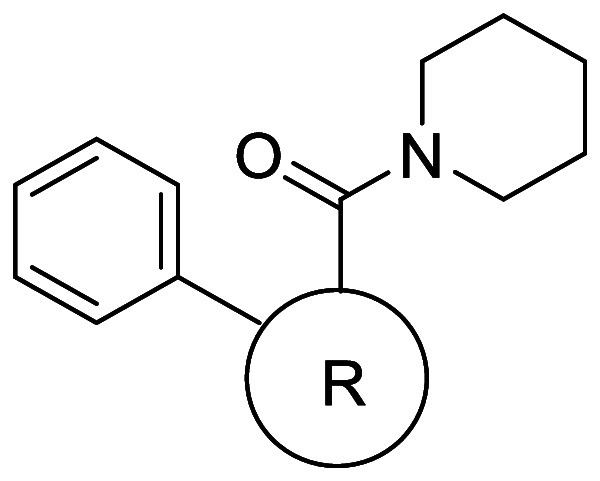

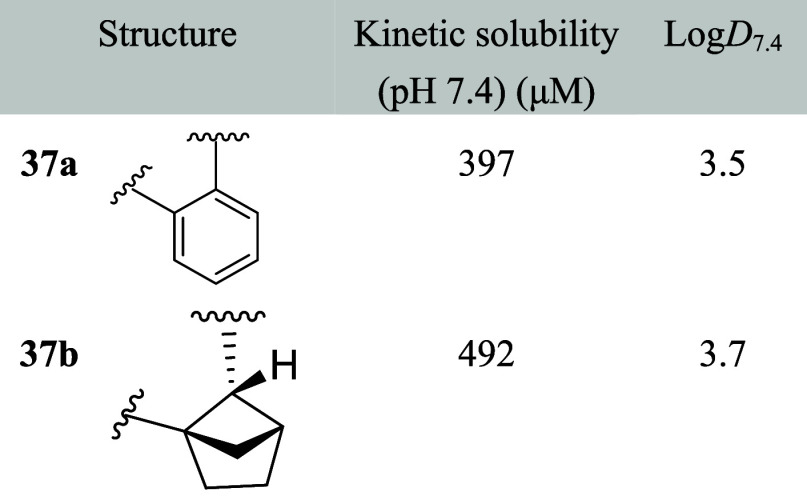
Solubility and Lipophilicity of the Bicyclo­[2,1,1]­hexane Analog

Baran et al. reported 1,2-disubstituted BCP as *ortho*- or *meta*-substituted phenyl mimetics.^65^ According to their matched molecular pair analysis, the kinetic solubility of the BCP **31c**, **38b**, **39b**–**d**, **40b**, and **43b** increased compared to the corresponding drugs, which are neutral at physiological pH, that is, sonidegib (**31a**) (15- to 20-fold), boscalid (**38a**) (24- to 34-fold), tolvaptan (**39a**) (about 1.6- to 5-fold), axitinib (**40a**) (over 67-fold), and telmisartan (**43a**) (over 16-fold) (Table 34). The authors noted that not much increase in solubility was achieved for drugs that are charged at physiological pH (meclizine and lomitapide (**42a**)). Interestingly, neutral phthalylsulfathiazole mimetics (**41b**–**c**) did not show solubility improvement, probably due to the high solubility of the parent drug (**41a**). They also examined Log*D*, permeability (RRCK), and hepatocyte stability. Unfortunately, many of the mimetics did not show improved permeability compared to the corresponding parent drugs. On the other hand, sonidegib mimetic **31c** showed over 40-fold higher permeability than sonidegib (**31a**). All BCP were separated in an enantiopure form by supercritical fluid chromatography, and the absolute stereochemistry of (−)-**40** was confirmed to be *S* by single-crystal X-ray crystallography.

**Table 34:**
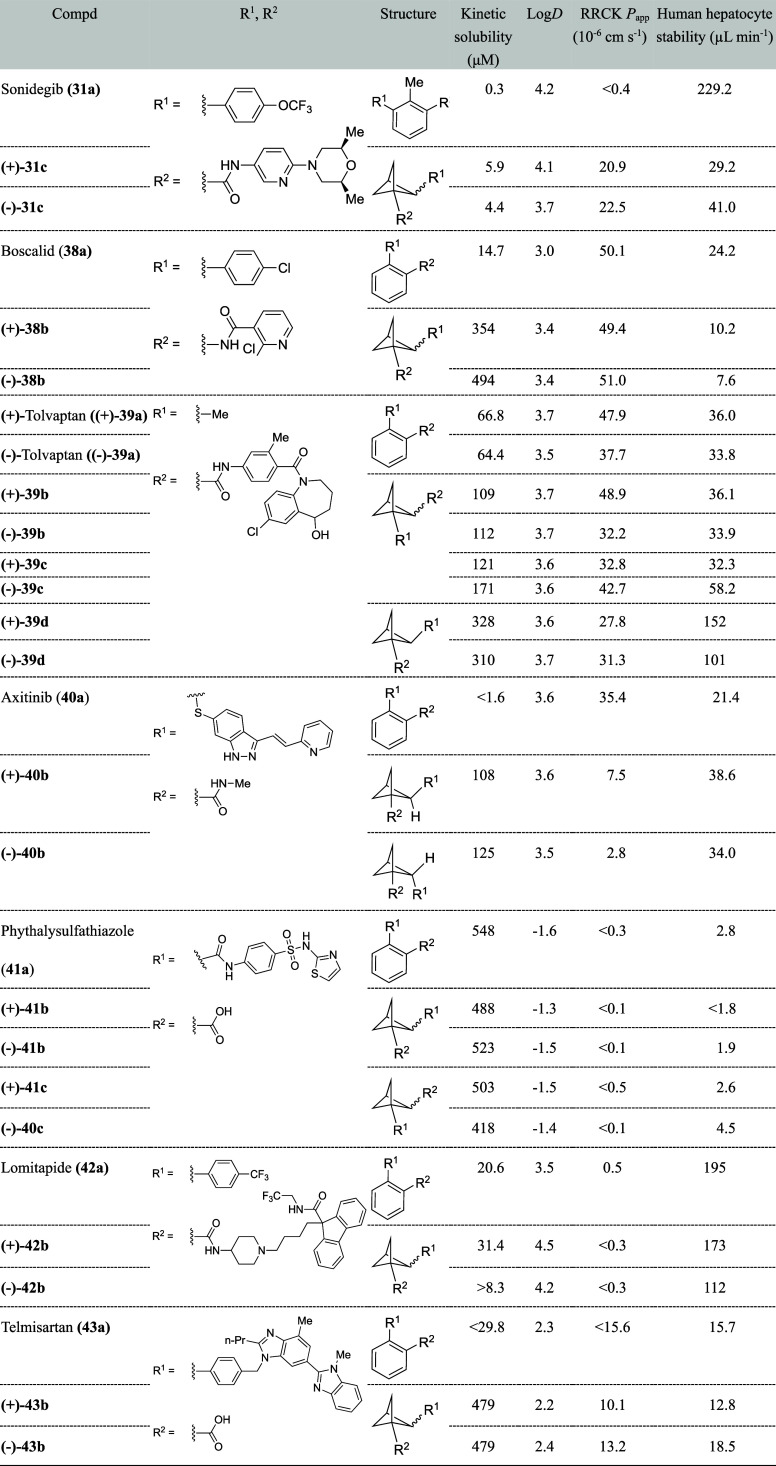
Physicochemical and Metabolic Profile of 1,2-Disubstituted BCP Analogs

#### 3.2.3 Bridged Heterocycles

There is increasing evidence that bridging basic heterocycles can lead to enhanced solubility due to not only reduced planarity but also increased basicity. Researchers at AstraZeneca reported that bridging morpholines and piperazines across the ring with one-carbon tethers reduces lipophilicity.^66^ For example, inhibitors of interleukin-1 receptor associated kinase 4 bearing bridged morpholines **44c** and **44d** exhibit lower Log*D*_7.4_ than the parent morpholine **44a**, whereas monomethyl analog **44b** shows increased Log*D* (Table 35). Bridged morpholine **44d** was found to be more basic than **44a**. Conformational analysis revealed that the bridges introduce a more rigid structure, which significantly reduces the total surface area of the molecules, thereby decreasing the lipophilicity. Additionally, the solvent accessibility of the morpholine oxygen atom is increased in the one-carbon-bridged analogs compared to both the monomethyl analogs and morpholine itself. The lower lipophilicity due to the one-carbon bridges can have multiple benefits, especially in cases of high susceptibility to metabolism or strong hERG inhibition, where these bridges may offer useful design solutions. Several matched molecular pairs (**45**–**47**) where one-carbon bridges resulted in increased p*K*_aH_, reduced Log*D*, reduced clearance in rats, and weaker hERG inhibition have been reported (see Figure 6).

**Table 35:**
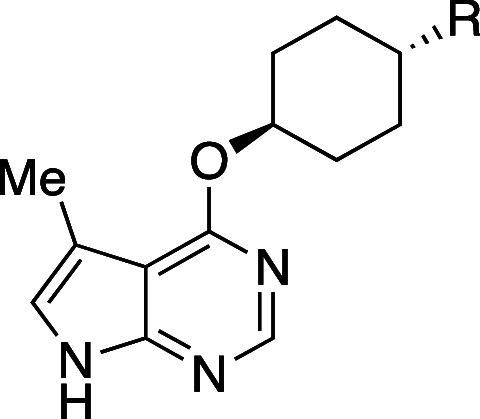

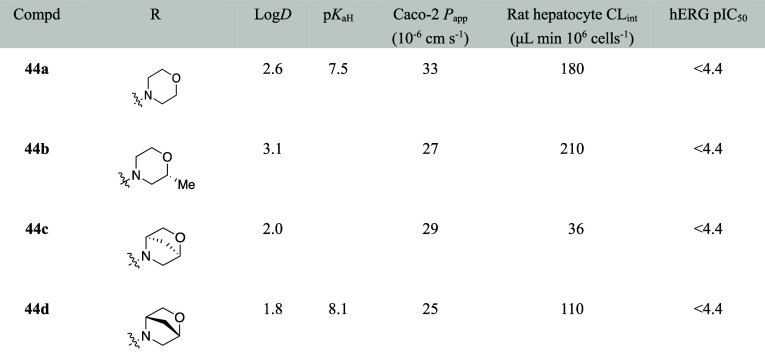
Decrease in Log*D* by Bridging Morpholino Group

**Figure 6:**
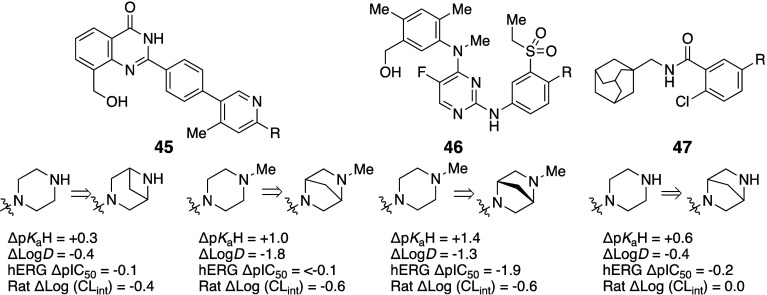
Decrease in Log*D* and increase in p*K*_aH_ by bridging piperazines.

Another study found that the addition of a one-carbon bridge to piperazine (**48b** and **48c**) and replacement with spirocyclic derivatives (**48d** and **48e**) decreased Log*D* and increased basicity compared to **48a** (Table 36).^67^ The bridges introduce a more rigid structure, leading to a reduced total surface area and decreased lipophilicity.

**Table 36:**
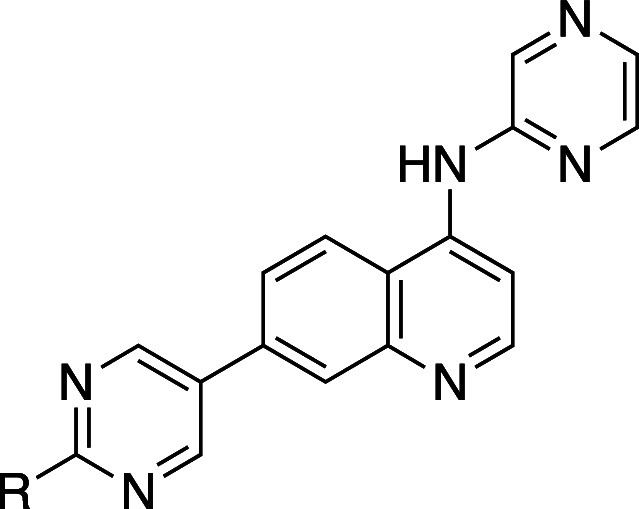

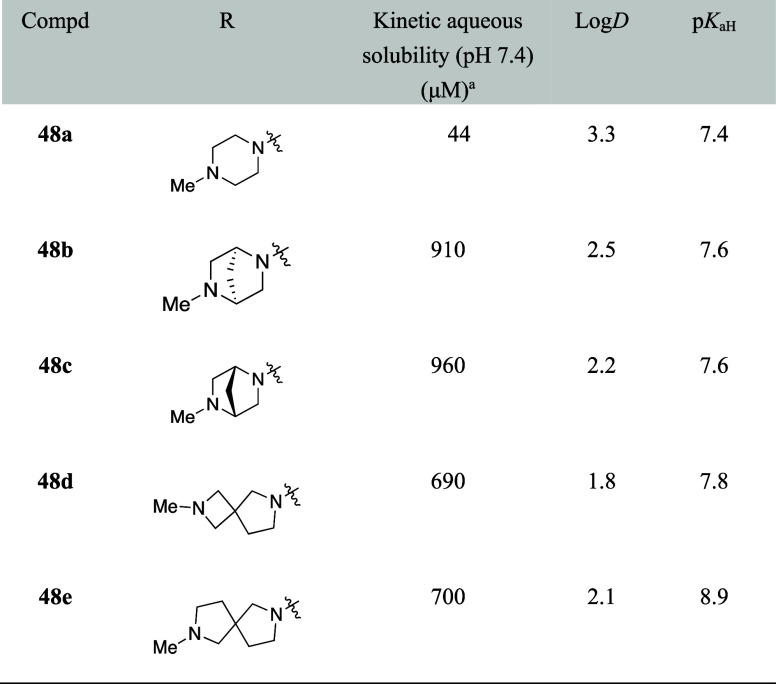
Decrease in Log*D* by Bridging Piperazine or Replacement with Spirocyclic Rings

a Solubility in phosphate buffer pH 7.4 from a solution in DMSO after 20–24 h.

In a separate report, the addition of a one-carbon bridge to piperazine (**49a**) led to a significant increase in aqueous solubility.^68^ The one-carbon-bridged analogue **49b** showed an over 1460-fold increase in aqueous solubility compared to **49a**, accompanied by a 60 °C decrease in melting point. This result suggests that disruption of planarity caused by the bridge at least partially contributes to the improvement in solubility. Interestingly, the tPSA of **49b** remains the same as that of **49a**, while the ClogP of **49b** is lower. Other analogs **49c** and **49d** also exhibit lower melting points, likely due to disrupted planarity. Replacement with a spirocyclic analogue **49i** resulted in the lowest melting point in this series, likely due to bending of the molecular structure (vide infra in Section 3.6). Another spirocyclic analogue **49j** showed over 480-fold improvement in aqueous solubility compared to **49a**. This improvement is likely attributed to a combination of factors, including a lower melting point caused by molecular bending and disruption of planarity as well as a decrease of Log*P* due to the presence of a secondary amino group. Compound **49j** (2 mg/kg) showed 27.4% oral bioavailability in mice, with similar AUC and Cmax values to **49a** (25 mg/kg), assuming linear pharmacokinetics (Table 37).

**Table 37:**
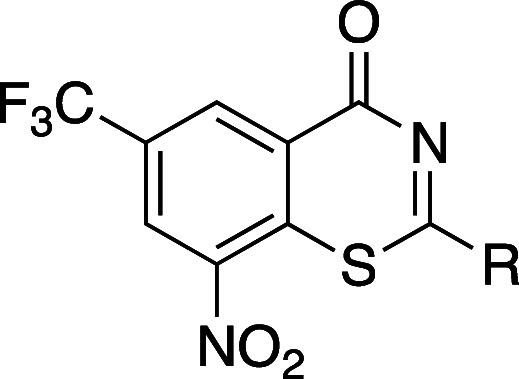

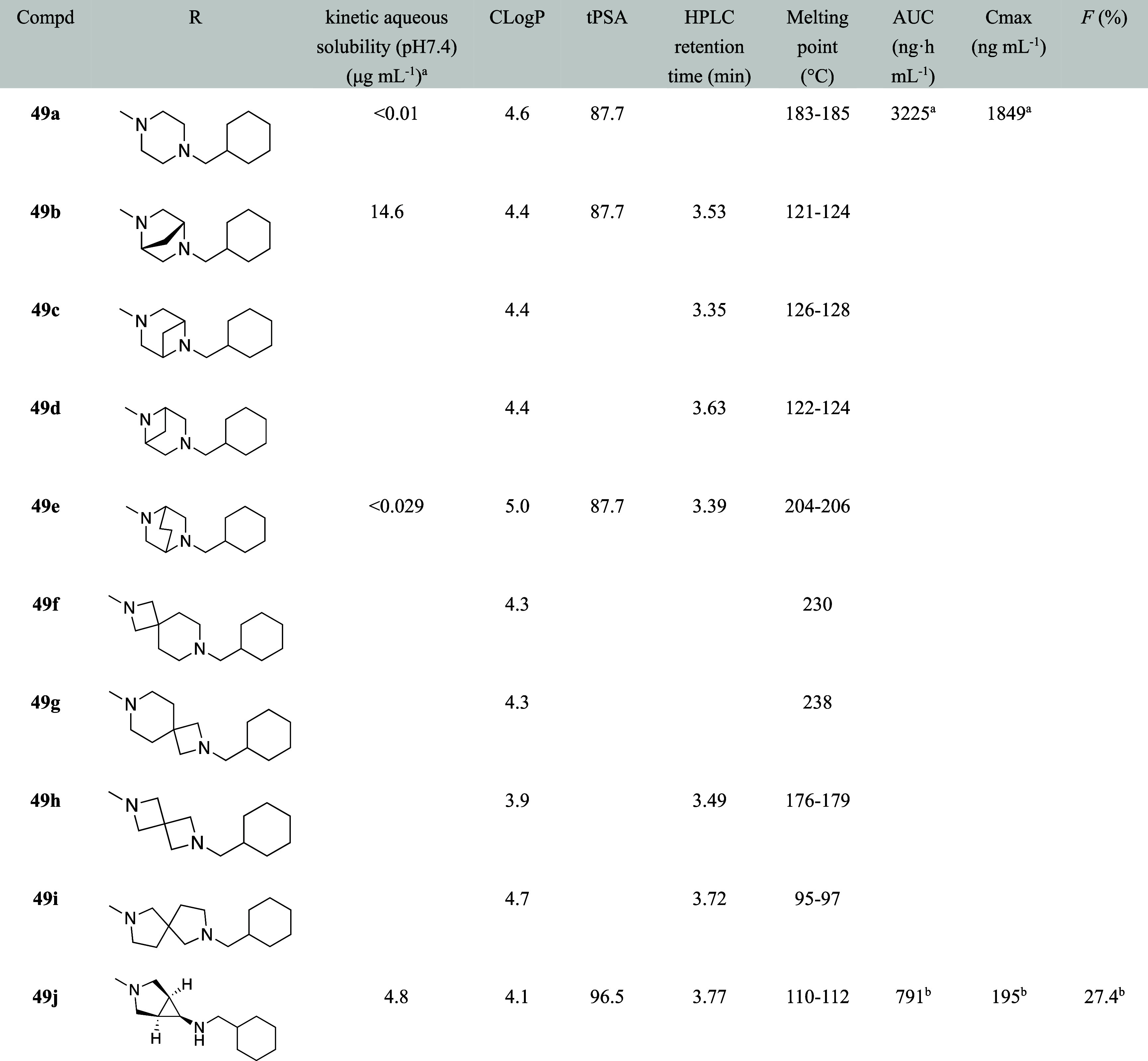
Improvement in Aqueous Solubility by Bridging Piperazine or Replacement with Spirocycles

a Mice were orally dosed with 25 mg/kg.

b Mice were orally dosed with 2 mg/kg.

### 3.3 Case Studies on Decreasing Intermolecular HBs (Aufheben of Hydrophobicity and Solubility)

While Ro5 predicts that the limited number of HBDs and HBAs is a good indicator of oral bioavailability, there are notable examples where druglikeness was improved by both a decrease in intermolecular HBs (Section 3.3) and an increase in intramolecular HBs (Section 3.4). This suggests that modern medicinal chemistry can benefit from more precise molecular design strategies that distinguish between intermolecular and intramolecular HBs.

Thalidomide (**50**) has low aqueous solubility due to the high melting point, probably caused by strong intermolecular HB in the solid state. Removal of the HBD by the introduction of a methyl group on the imide (**50b**) resulted in lower melting point and lower crystal density, leading to increasing aqueous solubility, even though the hydrophobicity of methyl analog **50b** is higher (Table 38).^69^

**Table 38:**
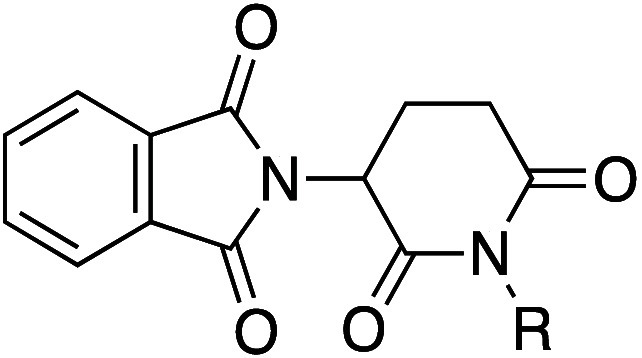
Improvement in Aqueous Solubility by Decreasing Intermolecular HB

compd	*R*	thermodynamic aqueous solubility (pH 6.4) (μg mL^–1^)	CLogP^a^	melting point (°C)	crystal density (g mL^–1^)
**50a**	H	52	0.53	275	1.48
**50b**	Me	276	1.2	159	1.43

a CLogP was estimated by us, using ChemDraw Ultra 20.0.

Upon oral administration, the cannabinoid receptor antagonist **51a** was inactive *in vivo*, whereas methyl analog **51b** was active *in vivo* (Table 39).^70^ Both **51a** and **51b** were poorly soluble in water (<1 mg/mL at pH 7). However, **51a** was moderately soluble in ethanol at reflux, while **51b** dissolved readily under the same condition. Single-crystal X-ray structural analyses and melting point measurements revealed that **51b** showed a less dense crystal packing than **51a**. In the case of **51a**, the two NH hydrogens form intramolecular HBs with both the dihydropyrazole moiety and the sulfonyl group. Additionally, **51a** forms intermolecular HBs between the SO_2_ oxygen atom and the amidine hydrogen atom of a neighboring molecule of **51a**. Introducing a methyl group at the amidine position (**51b**) disrupted these intermolecular HBs, leading to a reduced crystal density and melting point. Consequently, the higher aqueous solubility and dissolution rate of **51b** likely contributed to its improved *in vivo* oral activity by enhancing dissolution in the gastrointestinal tract.

**Table 39:**
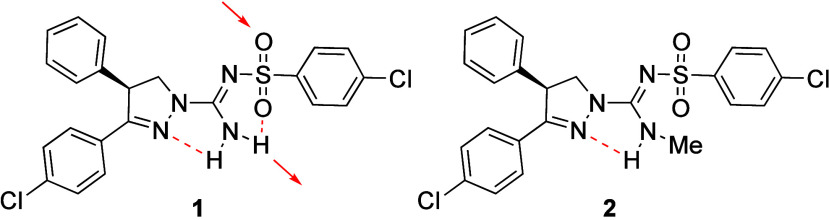
Improvement in Solubility by Decreasing Intermolecular HB

compd	*R*	kinetic solubility^a^	CLogP	melting point (°C)	crystal density (g cm^–3^)
**51a**	H	moderate	4.3	235	1.535
**51b**	Me	easy	4.8	170	1.481

a Solubility in EtOH at reflux intramolecular HBs are shown as red dotted lines. Intermolecular HBs are indicated by arrows.

Single-crystal structural analysis of androgen receptor antagonist **52a** revealed an intermolecular HB between the amide carbonyl group and the hydroxyl group of a neighboring molecule of **52a**, resulting in tight crystal packing and low aqueous solubility (Figure 7).^71^ To improve the solubility, the amide carbonyl group in the hydantoin moiety was removed to disrupt the intermolecular HB. Removal of this amide carbonyl (**52b**) resulted in a 13-fold increase of solubility over **52a**. The melting point of **52b** was also lower than that of **52a**, despite the higher CLogP. These results suggest that the reduction in crystal packing density due to the removal of the intermolecular HBAs was a key factor in the enhanced solubility of **52b**, despite the increased hydrophobicity.

**Figure 7:**
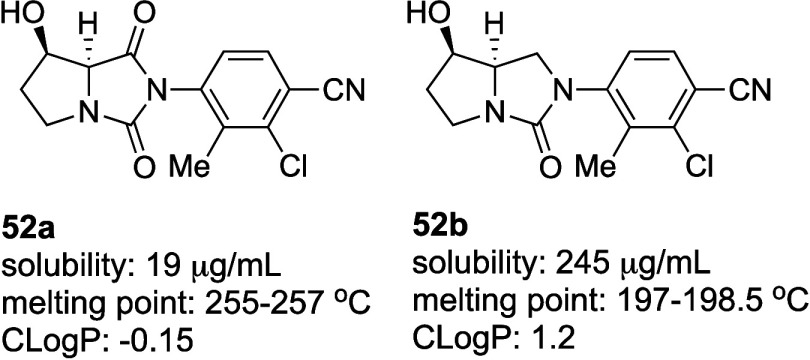
Improvement in solubility by decreasing intermolecular HB.

Cyclic pyrrolidinyl analog **53c** exhibited an approximately 3-fold improvement in aqueous solubility over the linear analog **53a** (Table 40).^35^ This improvement may be attributed to a decrease in intermolecular HB caused by a reduction in the number of HBAs, which is also consistent with the lower melting point of **53c** relative to that of **53a**. Another possible explanation is that the relatively bulky pyrrolidine moiety in **53c** disrupts the coplanarity between the amide group and the phenyl ring, further contributing to the solubility enhancement. In contrast, azetidinyl analogue **53b** showed lower aqueous solubility, despite having a lower CLogP, because of its higher melting point compared to **53c**.

**Table 40:**
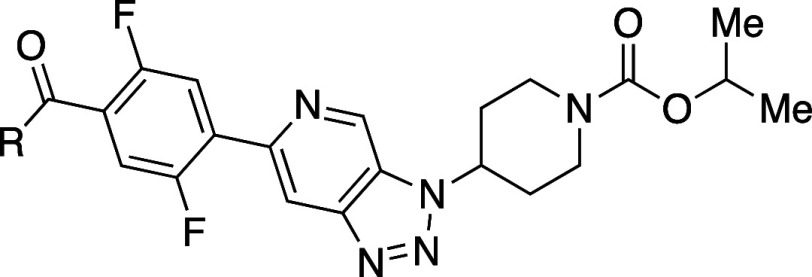

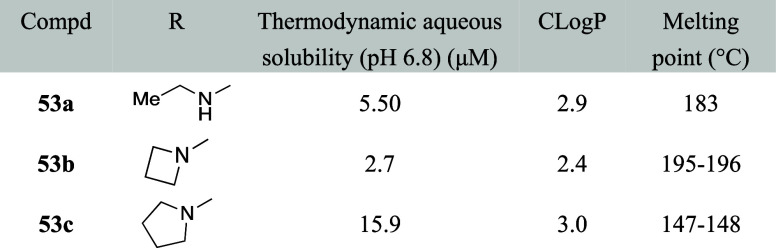
Improvement in Thermodynamic Aqueous Solubility by Decreasing Intermolecular HB

### 3.4 Case Studies on Increasing Intramolecular HBs (Aufheben of HB and Permeability)

Although Section 3.3 details several concrete examples of decreasing intermolecular HBs, aligning with the principles of Ro5, it is also important to note that an increase in intramolecular HBs improved both the solubility and permeability.

Introduction of a nitrogen atom (**54b**) at the *ortho* position of the 2-naphthamide analog of neurokinin receptor antagonist **54a** resulted in the formation of an intramolecular HB (Table 41). Isoquinoline analog **54b** showed improved aqueous solubility and permeability.^72^ Furthermore, **54b** exhibited a lower CLogP than that of **54a**, suggesting that the reduction in hydrophobicity also contributed to the enhancement of aqueous solubility.

**Table 41:**
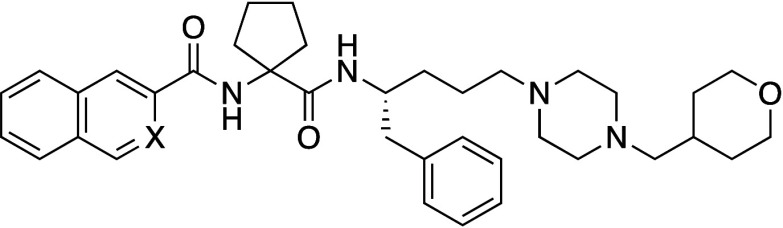
Improvement in Aqueous Solubility and Permeability by Increasing Intramolecular HB

compd	*X*	kinetic aqueous solubility (pH 6.4) (μg mL^–1^)	CLogP	PAMPA (10^–6^ cm s^–1^)	Caco-2 (10^–6^ cm s^–1^)
**54a**	CH	1.57	4.28	5.96	14.65
**54b**	N	2.80	3.14	15.5	25.20

Diastereomers **55a** and **55b** illustrate the remarkable impact of stereochemistry on physicochemical and biological properties (Table 42).^73^ *cis*-C8,C9 diastereomer **55b** exhibited 87-fold higher aqueous solubility than the *trans*-C8,C9 diastereomer **55a**. Mechanistic studies revealed that *cis-* **55b** had a Log*D* value of 3.9, which is 0.6 units lower than that of *trans-* **55a**. The most stable neutral conformations of amines **55a** and **55b** are both rigid due to a strong intramolecular HB between the amide N–H and the tertiary amine (Figure 8). However, differences in strain energy were observed: neutral *cis-* **55b** was 3.1 kcal/mol higher in free energy than *trans-* **55a**, and its cationic form was 1.2 kcal/mol higher, resulting in a total energy difference of 1.9 kcal/mol. Further investigations using ^1^H NMR spectroscopy supported these findings. In line with the increase in its Log*D* value, **55a** showed enhanced permeability in Caco-2 cells, and *cis-* **55b** demonstrated a significant increase in permeability in cells with a pH of 7.4 on the apical side compared to pH 6.5, whereas *trans-* **55a** showed no pH-dependent permeability changes. The results underscore the importance of preparing and evaluating pure stereoisomers in drug discovery or chemical probe projects, as stereochemistry can have profound effects on physicochemical, pharmacokinetic, and pharmacodynamic properties.

**Table 42:**
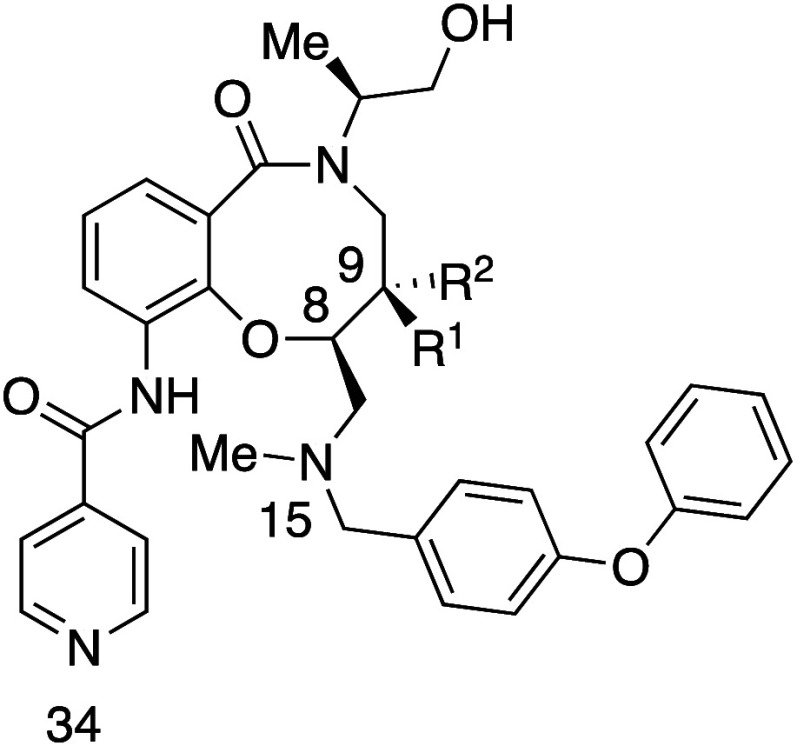
Difference of Aqueous Solubility between Diastereomers

compd	*R* ^1^	*R* ^2^	kinetic aqueous solubility (pH 7.4) (μM)^a^	Log*D*_7.4_	p*K*_aH_ (N34, pyridine)	p*K*_aH_ (N15, 3′-amine)	Caco-2 *P*_app_ pH 6.5/7.4 (10^–6^ cm s^–1^)	Caco-2 *P*_app_ pH 7.4/7.4 (10^–6^ cm s^–1^)
**55a**	H	Me	1	4.5	2.76	6.08	63.8	63.9
**55b**	Me	H	87	3.9	2.97	7.16	21.8	27.5

a Determined at pH 7.4 in PBS containing 1% DMSO.

**Figure 8:**
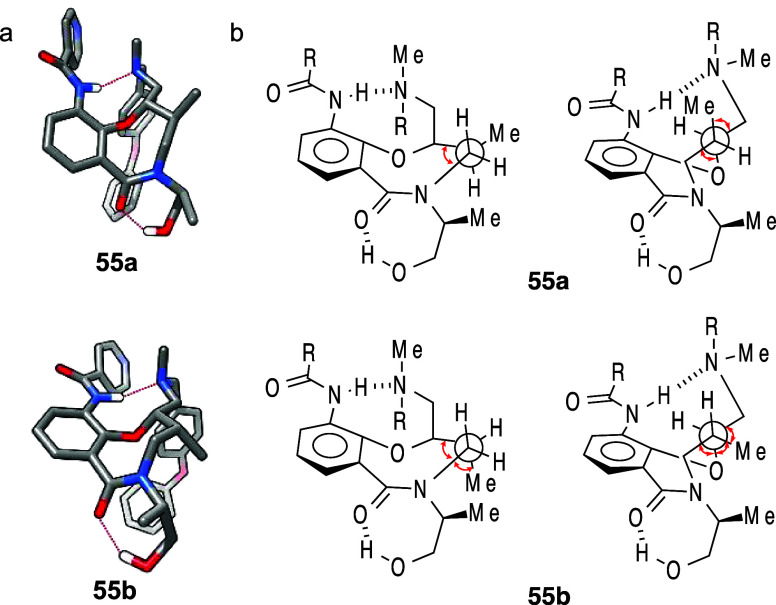
(a) The most stable conformations of the neutral forms of **55a** and **55b**. (b) Newman projections looking down the C10–C9 bond (left) and the C9–C8 bond (right) with gauche interactions highlighted with red arrows. Their stable conformations shown in panel (a) were generated using maestro (Schrodinger), and the images were generated by UCSF Chimera software.

### 3.5 Removal of Head-to-Head Interactions

Disruption of the intermolecular interaction between methylsulfonyl groups has been shown to improve solubility. For example, **56a** exhibited very low solubility (0.03 μM) and a high melting point (201–202 °C), despite lacking HBDs (Table 43).^32^ Single X-ray crystal structure analysis revealed that **56a** adopts a flat conformation, with efficient molecular stacking facilitated by strong polar interactions between sulfonyl groups and methyl group hydrogens (Figure 9). These head-to-head interactions align the molecules, resulting in a tight crystal lattice.

**Table 43:**
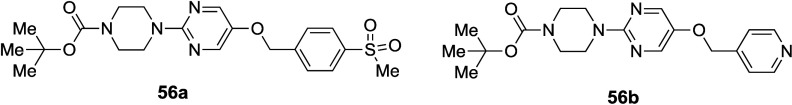
Improvement in Aqueous Solubility by Decreasing Intermolecular Interaction of the Sulfonyl Group

compd	aqueous solubility (pH7.4) (μM)	Log*D*_7.4_	melting point (°C)	crystal density (g cm^–3^)
**56a**	0.03	3.2	201–202	1.361
**56b**	23	3.4	147–148	1.277

a Shaken for 24 h.

**Figure 9:**
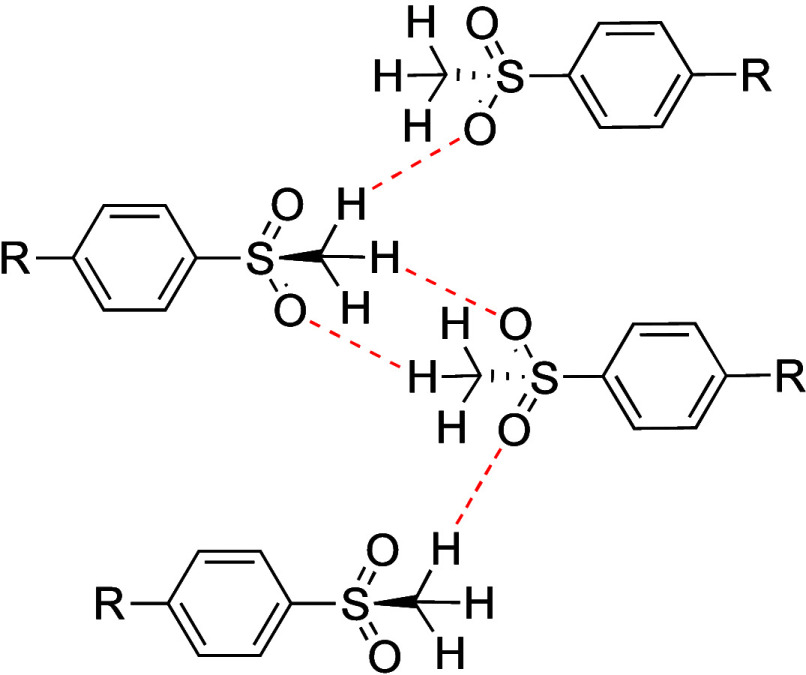
Head-to-head interaction of sulfonyl groups.

A search of the Cambridge Crystallographic Database indicated that 18% of methyl sulfone moieties are associated with head-to-head molecular interactions; another 18% are involved in ladder-like interactions, and 23% are implicated in both. In line with this analysis, replacing the (methylsulfonyl)­phenyl group in **56a** with a pyridyl group (**56b**) resulted in a 760-fold improvement in solubility, accompanied by a slight increase in Log*D*_7.4_. Single-crystal X-ray crystal analysis of **56b** confirmed the absence of head-to-head interactions and the network of polar interactions associated with the methyl sulfone group. This led to a reduced crystal density, a lower melting point, and improved solubility.

Compound **57a** exhibited low solubility in both the Japanese Pharmacopoeia first fluid (JP1, pH 1.2) and FeSSIF (Table 44).^74^ Substituting the methylsulfonyl group in **57a** with a dimethylcarbamoyl group (**57b**) improved the solubility by 24-fold in JP1 and 4-fold in FeSSIF, despite an increase in CLogP.

**Table 44:**
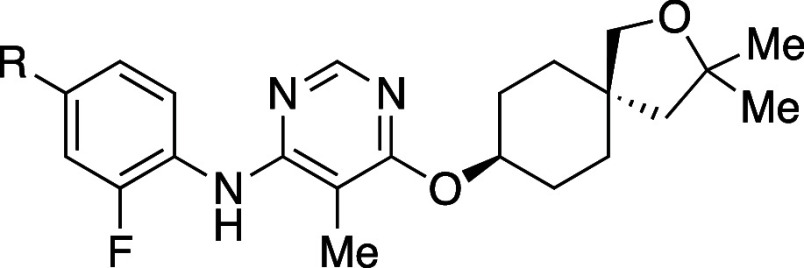

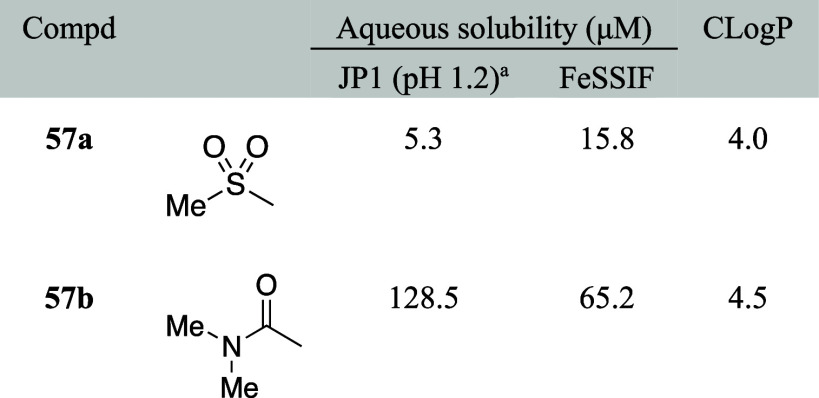
Improvement in Aqueous Solubility by Decreasing Intermolecular Interaction of Sulfonyl Group

a Japanese Pharmacopoeia first fluid for dissolution test adjusted to pH 1.2.

### 3.6 Bending Molecular Structure by Changing the Position of Substituents

Sections 3.1 and 3.2 discussed strategies to improve druglikeness by modulating molecular planarity, which refers to a molecule’s flatness or thickness (akin to the dimension perpendicular to the plane of depiction). In contrast, this section focuses on molecular linearity or bending, which describes a different axis of the molecular shape. Bending characterizes a molecule’s deviation from a straight line (i.e., its linearity or width), distinct from flatness. There’s increasing evidence that bending molecular structures is a strategy to improve druglikeness. The melting points of C18 *cis*-fatty acids decrease as the number of *cis*-double bonds increases. This is attributed to the introduction of bent molecular structures that disrupt crystal packing, which leads to lower melting points. Therefore, modifying linear molecular structures to create bent conformations is expected to be a promising strategy to enhance solubility by reducing intermolecular interactions.

For example, repositioning the tetrahydropyrimidylamino group from the 4-position of the piperidine ring in integrin antagonist **7a** to the 3-position (**7i**) led to a substantial increase in aqueous solubility (at least 35-fold) compared to **7a** (Table 45).^75^ Notably, this improvement was achieved without alteration of the molecular weight. Compound **7i** also exhibited greater hydrophobicity and a lower melting point compared to **7a**, highlighting the role of bent molecular shapes in improving solubility (Figure 10).

**Table 45:**
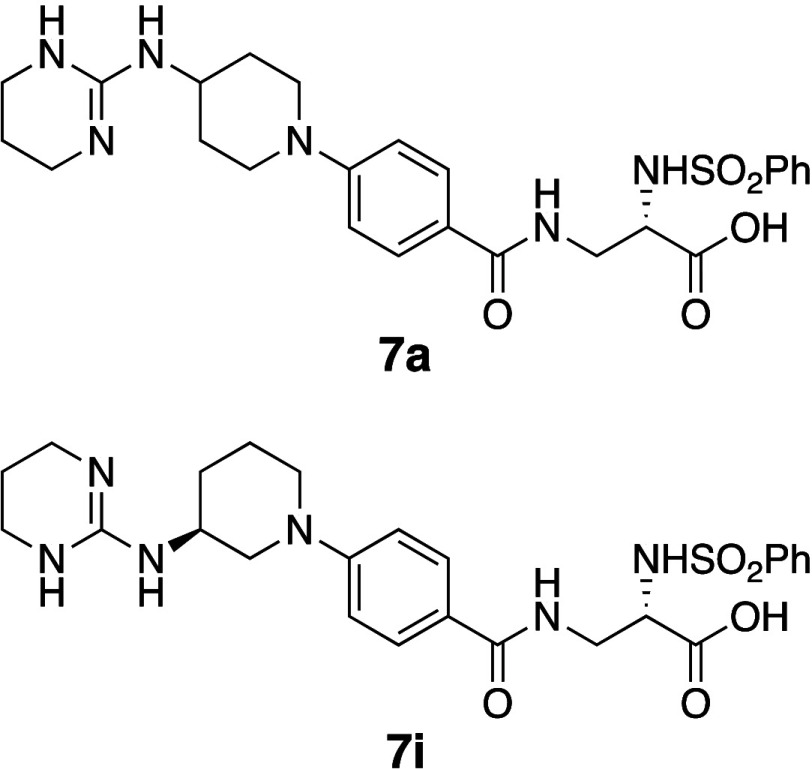
Improvement in Aqueous Solubility by Bending the Molecular Structure

compd	thermodynamic water solubility (mg mL^–1^)^a^	CLogP	HLPC retention time (min)^a^	melting point (°C)
**7a**	<0.1	1.1	8.25	252–254
**7i**	3.5	1.1	12.2	181–184

a Reversed-phased column.

**Figure 10:**
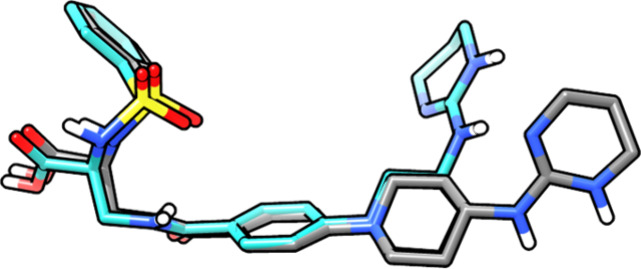
The overlay of **7a** (gray) and **7i** (cyan). Their stable conformations were generated using maestro (Schrodinger), and the images were generated by UCSF Chimera software.

Poor solubility of the retinoic acid receptor agonist **58a** was attributed to its high lipophilicity and linear chemical structure (Table 46), and bent analogs (**58b**–**58d**) showed dramatically increased solubility.^76^ The *meta*-substituted analog **58b** showed 22-fold higher solubility in a phosphate buffer/EtOH (7:3) mixture compared to **58a** and was also soluble (0.42 μg/mL) in a mixture of phosphate buffer and EtOH (9:1). The *ortho*-substituted analog **58c** showed 240-fold higher solubility and was also soluble in phosphate buffer alone (4.0 μg/mL). The most pronounced improvement was observed for **58d**, with an 890-fold increase in solubility compared to **58a**. As for the mechanism, the order of lower melting points (**58d** < **58c** < **58b** < **58a**) was the same as the order of higher solubility in a mixture of phosphate buffer and EtOH (7:3) (**58d** > **58c** > **58b** > **58a**). As regards hydrophobicity, the Log*P* values of **58b** and **58d** were almost the same as that of **58a**. These results indicate that the increased aqueous solubility of **58b** and **58d** is mainly due to loosening of the crystal structures due to bent molecular shapes.

**Table 46:**
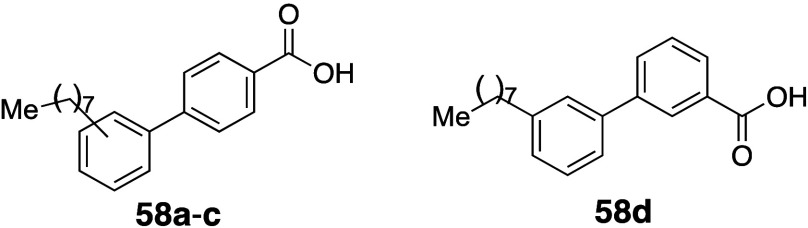
Improvement in Thermodynamic Aqueous Solubility by Bending the Molecular Structure

		thermodynamic aqueous solubility (pH 7.4) (μg mL^–1^)^a^					
compd	position	7:3	9:1	10:0	Log*P*	melting point (°C)	λmax (nm)
**58a**	*para*	8.4	<0.1	<0.1	4.8	151	280
**58b**	*meta*	190	0.42	<0.1	4.8	130.5–132.5	272
**58c**	*ortho*	2000	30	4.0	4.5	100.5	256
**58d**		7500	2.7	<0.1	4.7	80.9–81.1	<220

a Thermodynamic aqueous solubility in a mixture of phosphate buffer (pH 7.4) and EtOH.

Bent analogs of 2-nitroimidazopyrazinone, such as *meta*-phenylpyridines **59c** and **59d**, also showed significantly improved solubility compared to their *para*-phenylpyridine counterparts **59a** and **59b** (Table 47).^77^

**Table 47:**
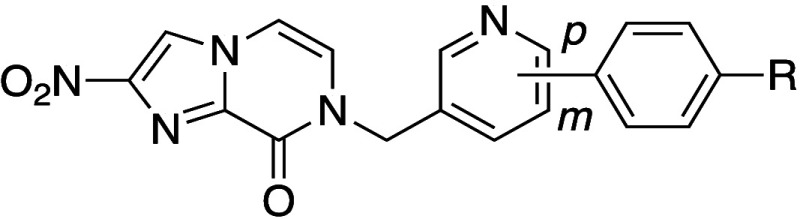
Improvement in Aqueous Solubility by Bending the Molecular Structure

compd	*meta* or *para*	R	kinetic aqueous solubility (pH 1) (μM)^a^	ALogP	HLPC retention time (min)^b^
**59a**	*para*	OCF_3_	<1	3.9	2.94
**59b**	*para*	Me	7.8	2.3	2.69
**59c**	*meta*	OCF_3_	16	3.5	2.87
**59d**	*meta*	Me	27	1.8	2.61

a A solution of the compound in DMSO was aliquoted into 0.1 M HCl (pH 1) and shaken for 24 h at room temperature.

b Reversed-phase column.

Substitution of a piperazine ring with a homopiperazine ring is another effective strategy to improve solubility. Compound **60a** exhibited limited aqueous solubility (44 μM) (Table 48), but replacing its *N*-methyl piperazine group with *N*-methyl homopiperazine (**60b**) resulted in a 23-fold increase in solubility (Table 48).^67^ This improvement was attributed to increased basicity and reduced Log*D* of **60b**, as well as a lower melting point (205–216 °C for **60b** vs 240–258 °C for **60a**), suggesting that bending the molecular structure resulted in reduced intermolecular interaction, which in turn at least partially contributed to the improved solubility.

**Table 48:**
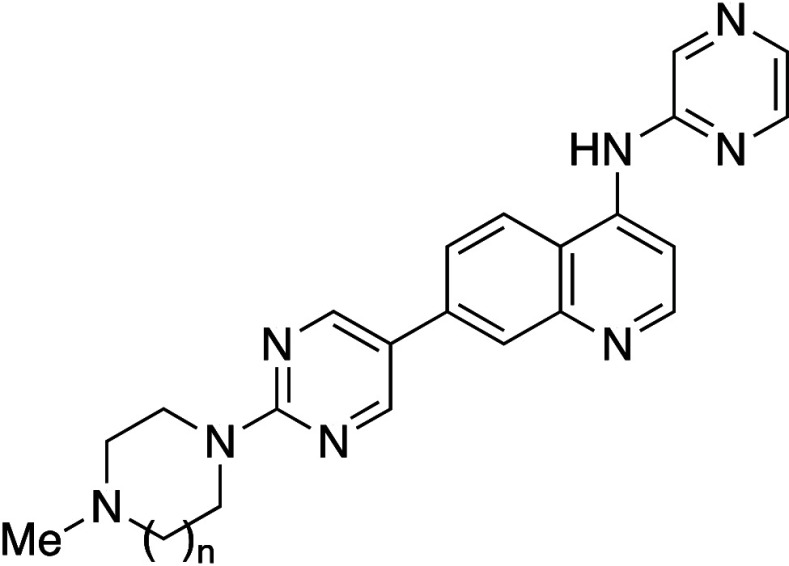
Improvement in Aqueous Solubility by Bending the Molecular Structure

compd	*n*	kinetic aqueous solubility (pH 7.4) (μM)^a^	Log*D*	p*K*_aH_	melting point (°C)
**60a**	1	44	3.3	7.4	240–258
**60b**	2	990	2.6	8.3	205–216

a Solubility in phosphate buffer pH 7.4 from a solution in DMSO after 20–24 h.

Substitution of the benzene ring of **61a** with pyrazole rings (**61b** and **61e**) led to 3-fold and 12-fold improvements in solubility, respectively, accompanied by reductions in CLogP and melting point (Table 49).^78^ Furthermore, substituting the 3-position methyl group of quinolone in **61b** with an ethyl group (**61d**) decreased the melting point and increased solubility, despite a concomitant increase in CLogP. This result was likely due to disruption of the planarity and increased entropy.

**Table 49:**
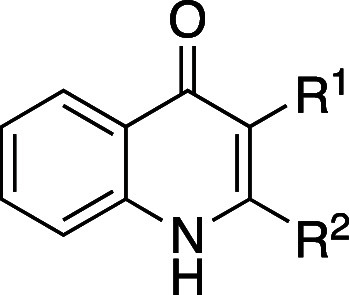

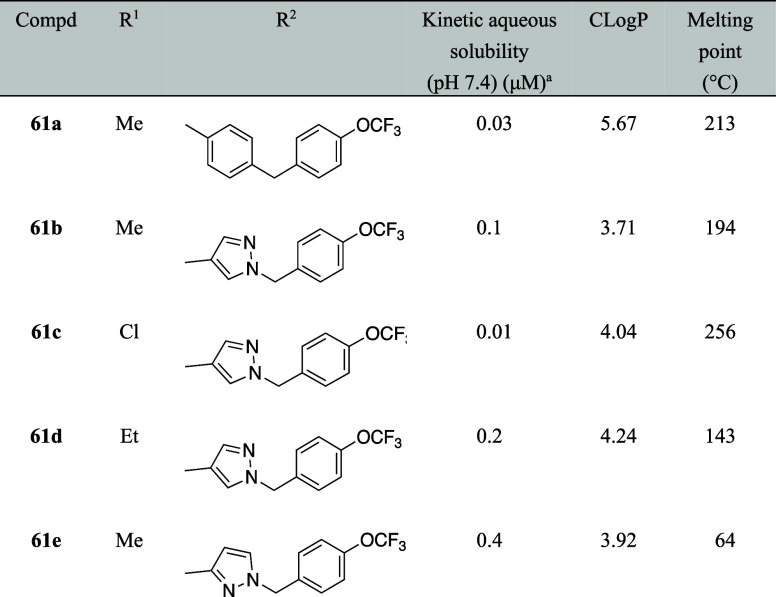
Improvement in Aqueous Solubility by Bending the Molecular Structure

Azobenzenes are a group of photoswitchable molecular machines that can exist in either *trans* or *cis* form. *trans*-Azobenzenes have planar conformations that are thermodynamically more stable and can be generated by visible light irradiation or spontaneously by thermal isomerization. On the other hand, irradiation of azobenzenes with UV light generates the *cis* isomers. *cis*-Azobenzene adopts a bent conformation with its phenyl rings twisted about 55° out of the plane of the azo group. Therefore, we hypothesized that *cis*-azobenzenes would possess better aqueous solubilization than the corresponding *trans* isomers as a result of weaker intermolecular interactions. Indeed, the aqueous solubilization of azobenzene **62a** could be controlled reversibly by irradiation with UV and visible light (Figure 11), and it varied depending on the UV irradiation wavelength and intensity. The solubilization of compound **62b** in phosphate buffer was increased by up to 20-fold by exposure to UV irradiation, compared to that without irradiation.^79^

**Figure 11:**
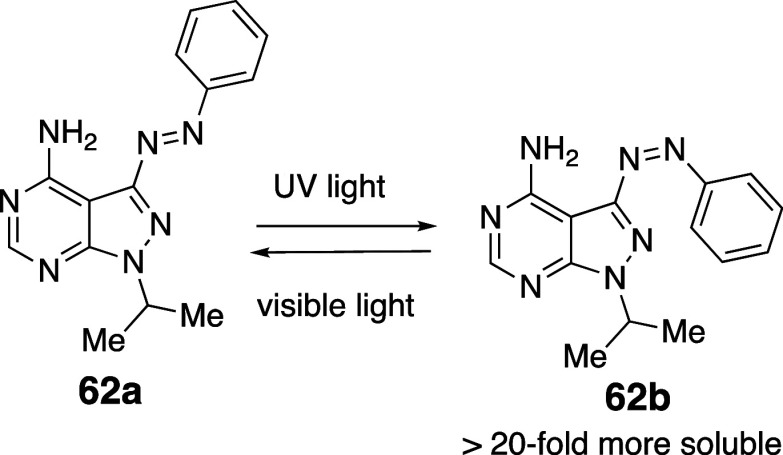
Improvement in aqueous solubilization of azobenzene **62a** by *cis*-isomerization.

### 3.7 Disruption of Molecular Symmetry

For simple compounds, higher molecular symmetry (quantified by the symmetry number, σ) is generally associated with higher melting point,^80,81^ though the relationship between molecular symmetry and physicochemical properties in more complex pharmaceutical compounds remains underexplored. The estrogen receptor antagonist cyclofenil (**63a**), for example, exhibits poor aqueous solubility (<1 μg/mL in 0.067 M phosphate buffer) due to its symmetric molecular structure (σ = 2; point group: C_2_v, considering conformational isomerization of the cyclohexyl group) (Table 50). To improve the solubility, the molecular symmetry was disrupted by introducing alkyl groups, yielding desymmetric analogs **63c**, **63d**, and **63e** (point group: Cs) and (*R*)-**63f** (point group: C_1_).^82^ These modifications significantly enhanced the aqueous solubility, despite the associated increase in hydrophobicity. For example, **63c** showed a 17.3-fold improvement in solubility over its symmetric isomer **63b**, and (*R*)-**63f** was 7.7-fold more soluble than symmetric **63g**. These increases in solubility were correlated with reductions in both the melting point and crystal density, suggesting that disruption of intermolecular interactions contributed to the observed improvement. Additionally, (*R*)-**63f** possesses a chiral center, further breaking molecular symmetry. Interestingly, the presence of chiral centers has been associated with a higher success rate in progressing from discovery to clinical testing and eventual drug approval.^20^ This study underscores the importance of incorporating chirality into molecular design to achieve better druglike properties, including enhanced solubility and membrane permeability.

**Table 50:**
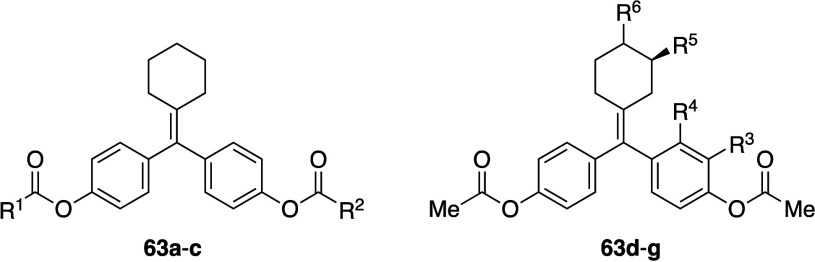
Improvement of Thermodynamic Aqueous Solubility by Disruption of Molecular Symmetry

compd	*R* ^1^	*R* ^2^	*R* ^3^	*R* ^4^	*R* ^5^	*R* ^6^	thermodynamic aqueous solubility (pH 6.8) (μg mL^–1^)^a^	Log*P*	melting point (°C)	crystal density (g cm^–3^)	dihedral angle (°)^b^
**63a**	Me	Me					7.76	4.91	137.5	1.268	55.3
**63b**	Et	Et					0.622	6.02	139.0		
**63c**	Me	*n*-Pr					10.8	6.00	69.0	1.236	
**63d**			Me	H	H	H	10.3	5.26	114.0		54.4
**63e**			H	Me	H	H	23.6	5.12	99.9		66.1
(*R*)-**63f**			H	H	Me	H	27.8	5.34	92.0	1.227	
**63g**			H	H	H	Me	3.61	5.41	137.0	1.243	

a Solubility in a mixture of phosphate buffer (pH 6.8) and EtOH (6:4).

b The most stable forms were estimated with Spartan’18.

### 3.8 Introduction of an Out-of-Plane Substituent at the *meta* Position of a Phenyl Group^34^

An analysis of the melting points of substituted benzene regioisomers (161 sets) revealed no significant difference in melting points between *ortho-* and *meta-*isomers when general substituents were considered. However, *ortho*-isomers bearing flat substituents tended to exhibit the lowest melting points, as discussed in Section 3.1.1. This indicates that the rule that *meta*-substitution leads to improved solubility should exist. To explore this idea, we defined a plane substituent as the one possessing an extension in two dimensions, collected and analyzed the dihedral angles of substituents bearing an sp^2^ atom from the Cambridge Structural Database, and divided them into plane and out-of-plane substituents (plane substituents: Ph, CO_2_H, Ac, NO_2_, CONH_2_, and NHAc; out-of-plane substituent groups: Me, Et, *t-*Bu, F, Cl, Br, CF_3_, CN, NH_2_, OH, OMe, SO_2_NH_2_, and SO_2_Me). Among compounds with out-of-plane substituents in the above 161 sets, *meta*-isomers were observed to have the lowest melting points among the regioisomers. We then validated this trend in pharmaceutical compounds with more complex structures using lead compounds with out-of-plane substituents such as **64a**, **65a**, and the androgen receptor antagonist bicalutamide eutomer **66i**. Across six of eight sets of pharmaceutical compounds bearing disubstituted benzene rings with out-of-plane substituents (Table 51), the *meta*-isomers showed the lowest melting points and the highest thermodynamic aqueous solubility among the isomers. These findings highlight that *meta*-substitution with out-of-plane groups is a promising strategy to interfere with crystal packing, thereby improving the aqueous solubility of pharmaceutical compounds.

**Table 51:**
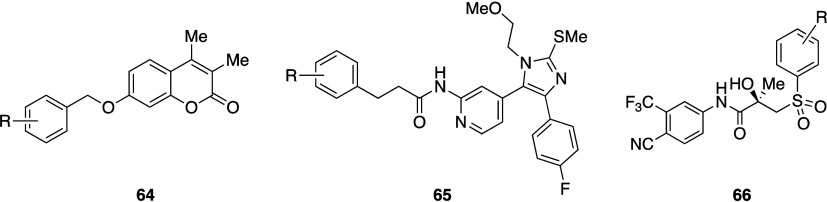
Improvement in Thermodynamic Aqueous Solubility by Introduction of a Out-of-plane Substituent

compd	*R*	thermodynamic aqueous solubility (pH 7.4) (μg mL^–1^)	Log*P*	melting point (°C)
**64a**	*o*-Me	5.2^a^	4.6	142.6
**64b**	*m*-Me	21.4^a^	4.7	112.0
**64c**	*p*-Me	19.1^a^	4.8	121.3
**65a**	*o*-OMe	0.0255^b^	4.3	132.1
**65b**	*m*-OMe	0.275^b^	4.1	90.9
**65c**	*p*-OMe	0.0473^b^	4.1	142.1
**66a**	*o*-Me	12.6^b^	2.3	139
**66b**	*m*-Me	18.8^b^	2.2	125
**66c**	*p*-Me	11.8^b^	2.2	161
**66d**	*o*-Et	19.3^b^	2.8	117
**66e**	*m*-Et	49.2^b^	2.7	48
**66f**	*p*-Et	7.9^b^	2.7	139
**66g**	*o*-F	27.2^b^	1.8	157
**66h**	*m*-F	4.1^b^	2.0	194
**66i**	*p*-F	14.6^b^	1.9	184
**66j**	*o*-Cl	13.6^b^	2.1	145
**66k**	*m*-Cl	29.3^b^	2.5	113
**66l**	*p*-Cl	5.4^b^	2.4	173
**66m**	*o*-Br	8.4^b^	2.3	187
**66n**	*m*-Br	56.3^b^	2.6	131
**66o**	*p*-Br	11.6^b^	2.5	163
**66p**	*o*-OMe	69.9^b^	1.8	145
**66q**	*m*-OMe	30.6^b^	2.1	125
**66r**	*p*-OMe	14.7^b^	2.0	155
**66s**	H	7.0^b^	1.9	181

a Thermodynamic aqueous solubility in a mixture of phosphate buffer (pH 7.4) and EtOH (7:3).

b Thermodynamic aqueous solubility in phosphate buffer (pH 7.4).

## 4 Improvement in Aqueous Solubility and Permeability of bRo5 Molecules (Aufheben of MW and Druglikeness)

Recent advancements in biology and medical sciences have led to the identification of novel drug targets for which traditional drug discovery approaches, such as screening for enzyme inhibitors, are ineffective, and new strategies have been developed to tackle these challenging targets.^83^ However, the designed molecules, including macrocycles and bifunctional molecules such as PROTACs, often lie in the bRo5 chemical space due to their high MW and structural complexity. This shift has underscored the importance of studying factors influencing the druglikeness of bRo5 molecules, particularly their aqueous solubility and membrane permeability. This section summarizes key findings on the solubility and permeability of bRo5 molecules and discusses guidelines for developing druglike bRo5 compounds.

### 4.1 Solubility and Permeability of bRo5 Molecules

According to the solute dissolution model shown in Figure 1, water forms a cavity to accommodate solute dispersion during the second step of the process. The formation of this cavity incurs a free energy penalty primarily due to the entropy loss of water. This penalty is particularly pronounced for larger solutes (i.e., high-MW molecules), making cavity formation less favorable compared to that of smaller solutes. Consequently, as the MW of a compound increases, its solubility tends to decrease. Similarly, membrane permeability inversely correlates with MW. These characteristics have been validated through multiple analyses of physicochemical parameters using proprietary pharmaceutical company data and academic databases.^84–86^ However, the MW is not the sole determinant of solubility and permeability. The solute dissolution model encompasses multiple steps beyond cavity formation, making MW only one of several contributing factors, and thus, the solubility of high-MW molecules can be improved by leveraging other parameters. For example, Gleeson’s analysis of solubility assay data for 44,584 molecules at GlaxoSmithKline demonstrated that ionization state significantly influences solubility.^84^ Additionally, Tolls et al. investigated the relationship between molecular size and aqueous solubility using C10- to C19-alkanes, highlighting the impact of molecular conformation.^87^ Their free energy simulations of solvation revealed that folded conformations are energetically more favorable than all-*trans* conformations as folding reduces cavity size and minimizes the associated free energy penalty. Thus, molecular designs that modulate ionization states and promote favorable folding can enhance the solubility of bRo5 drugs. Regarding permeability, Gleeson et al. observed a complex relationship between ionization state and permeability, mediated by lipophilicity. While neutral and basic molecules exhibit similar permeability, acidic and zwitterionic molecules are generally less permeable. Interestingly, zwitterionic molecules with CLogP < 3 are less permeable than acidic molecules, whereas highly lipophilic zwitterionic molecules (CLogP > 5) are more permeable.^84^ These findings suggest that factors other than MW, such as lipophilicity and ionization state, also play crucial roles in determining the permeability of high-MW molecules.

Although hydrophilicity and ionization state also affect the solubility and permeability of small molecules, molecular design for optimal solubility/permeability in bRo5 molecules differs significantly due to their more three-dimensional structures. In the following sections, we summarize key studies and discuss parameters that should be considered in designing bRo5 molecules with favorable druglikeness.

### 4.2 Lessons from Orally Available bRo5 Drugs

Over the past decade, increasing numbers of orally available bRo5 drugs, encompassing both naturally occurring molecules and synthetic compounds, have been reported. Analyses of these successful cases from various perspectives have provided valuable guidelines for designing bRo5 molecules with favorable druglikeness. This section highlights the lessons learned so far.

#### 4.2.1 3D Polarity: a Determinant of Solubility of bRo5 Molecules

In the hydration step of the solute dissolution model (Figure 1), polarity emerges as a critical factor for improving solubility. Whitty et al. analyzed 20 clinically approved, orally available macrocycles and proposed that achieving good solubility of high-MW bRo5 drugs requires tPSA of more than 0.2 Å^2^ per unit of MW.^88^ However, while predicting the solubility of bRo5 compounds might appear straightforward based on polarity parameters such as tPSA, actual polarity depends on the compound’s conformation in water.^89^ Kihlberg and colleagues demonstrated that the difference between tPSA and maximum molecular 3D PSA increases with MW.^90^ Notably, their analyses showed that the coefficient of determination (*r* ^2^) for Log*S* vs 3D PSA was higher than that for Log*S* vs tPSA, underscoring the importance of incorporating conformational data when assessing solubility.

Jerhaoui et al. developed orally available Mcl-1 protein–protein interaction (PPI) inhibitors with macrocyclic structures and MW exceeding 650 (Table 52).^91^ Beginning with the poorly soluble Mcl-1 PPI inhibitor **67** (AMG176), the authors optimized the compound to obtain **71a**, which exhibited significantly improved solubility. Interestingly, during this optimization process, a single stereochemical change was found to influence the solubility. For example, **70b** showed markedly lower solubility and higher lipophilicity than its epimer **70a**. Similar stereochemistry-dependent effects were observed in comparisons between **71a** and **71b** and between **71c** and **71d**. Molecular dynamics (MD) simulations of **71c** and **71d** revealed that stereochemical changes altered the conformation, reducing the polar fraction of the total solvent-accessible surface area in **71d** compared to **71c**. These findings highlight the role of specific 3D conformations in water in determining the aqueous solubility.

**Table 52:**
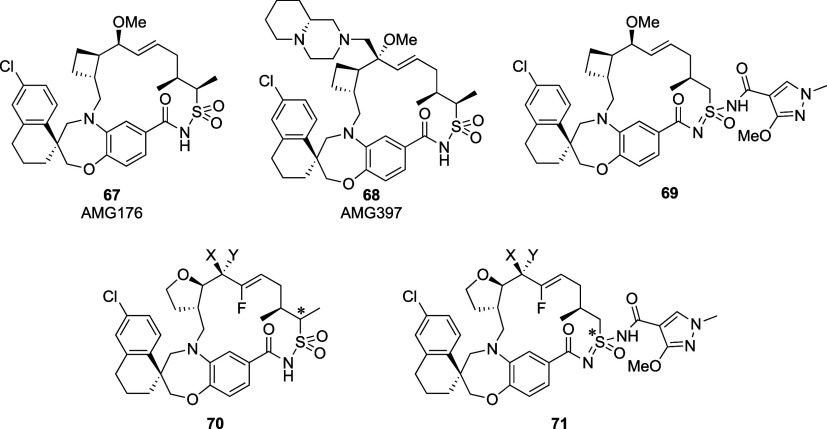

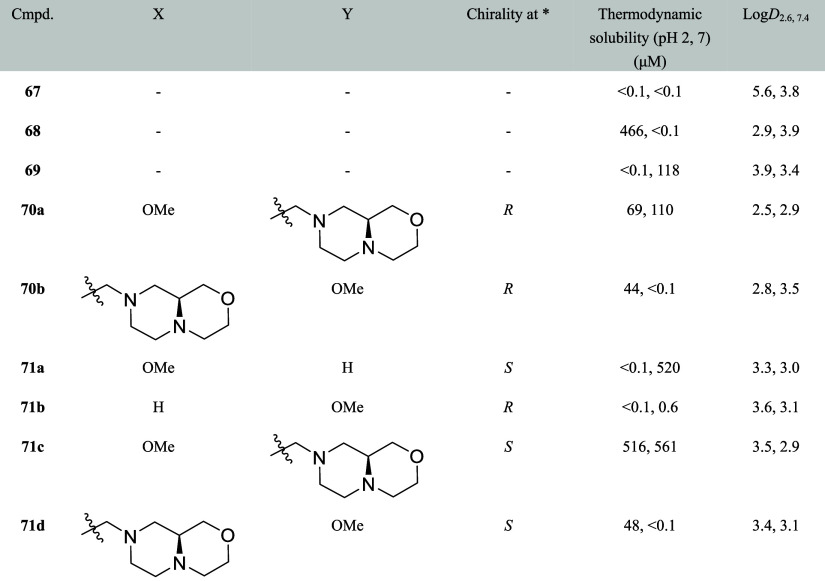
Structures, Activities, and Properties of the Mcl-1 Inhibitors

#### 4.2.2 Chameleonicity: a Property Explaining Why bRo5 Drugs Can Be Both Soluble and Permeable

The cellular membrane is a hydrophobic environment that favors lipophilic molecules. This characteristic was highlighted in a comprehensive permeability analysis of over 200 macrocycles conducted by Kihlberg et al., which established a strong positive correlation between lipophilicity and cell permeability.^92^ Conversely, parameters related to polarity, such as the number of HBDs/HBAs and charge, were found to restrict the permeability.

Naturally occurring cyclic peptides and macrocycles with diverse scaffolds often exhibit good passive permeability and solubility despite their large MW, typically violating Ro5.^93^ Cyclosporine A (CsA), for instance, is a clinically used immunosuppressive drug that is orally available. Extensive physicochemical and computational studies on CsA and CsA-inspired synthetic cyclic peptides have provided insights into the reasons for their membrane permeability.^94^ A key property underlying their behavior is conformational flexibility, which enables these compounds to extend their structures in aqueous environments to expose polar functional groups (HBDs and HBAs) for hydration while folding to shield these groups in the hydrophobic cellular membrane.^95–99^ This behavior, termed “molecular chameleonicity”, arises from environment-dependent intramolecular HB switching. In lipophilic environments, such as the lipid bilayer, intramolecular HBs reduce the number of exposed HBDs and HBAs. This widely accepted mechanism has been shown to apply not only to cyclic peptides but also to bRo5 synthetic clinical candidates,^90^ macrocycles,^100^ antiviral drugs,^101^ and PROTACs.^102^

Several research groups have explored the prediction and evaluation of bRo5 molecular permeability and druglikeness through chameleonicity theory. For instance, Ermondi et al. employed chameleonicity as a guideline for designing bRo5 drugs based on physicochemical properties.^103^ They investigated model molecules by comparing experimental and computational predictions of chameleonicity. Experimentally, changes in capacity factors in a PLRP-S column system eluted with varying organic phase ratios were used to assess chameleonic behavior, reflecting changes in lipophilicity due to environment-dependent conformational shifts. Computationally, differences in conformational sampling between water and CHCl_3_ were analyzed, and strong agreement was observed between experimental and predicted results. Price et al. introduced a high-throughput chameleonicity descriptor, the experimental PSA (EPSA)-to-tPSA ratio (ETR). EPSA, determined by a supercritical fluid chromatography (SFC)-based method, leverages a low dielectric constant mobile phase that promotes intramolecular HB formation.^104^ Thus, the ETR (EPSA per tPSA) quantifies a compound’s ability to reduce its effective polarity due to external influences. Their analysis of a data set using ETR established simple MW and ETR thresholds for good permeability: ETR ≤0.8 for MW 500–800 and ≤0.6 for MW 800–1000. Lokey et al. proposed lipophilic permeability efficiency (LPE) as a robust metric for quantifying membrane permeability.^105^ For compounds with AlogP values below 4, they observed a strong correlation between the experimental decadiene–water distribution coefficient (Log*D*_dec/w_) and PAMPA, even distinguishing stereoisomers. They further established a linear relationship between AlogP, which is known to correlate with aqueous solubility, and Log*D*_dec/w_. Based on these findings, the authors derived the equation: LPE = 1.06 × AlogP – 5.47. A higher LPE value indicates a greater membrane permeability.

Interestingly, structural modifications of drugs often result in relatively consistent changes in LPE. For example, methylation of a solvent-exposed amide NH increased the LPE by approximately +1.0. This allows for the estimation of changes in physicochemical properties based on the ΔLPE values.

Theoretical studies on the membrane permeability of bRo5 molecules have increasingly attracted the attention of computational chemists and chemoinformaticians. Numerous studies have simulated solvent-dependent conformational flexibility of bRo5 molecules, particularly cyclic peptides, and computational predictions of druglikeness for these compounds have been explored.^106–108,96^

#### 4.2.3 *N*-Methylated Amide Bond and Ester Bond: Strategies for Improving ADME of bRo5 Molecules

*N*-methylated amide bonds and ester bonds are frequently found in naturally occurring cyclic peptides and are commonly employed to enhance the permeability of not only small molecules (see Section 3.3), but also cyclic peptide-based bRo5 drugs.^109,110^ It has been shown that the number of *N*-methylations does not correlate with the permeability of cyclic peptides.^110^ However, *N*-methylation at the peptide backbone often induces conformational changes, as *N*-alkylated amide bonds, such as those in proline-containing peptides and peptoids, are known to promote *cis*-*trans* isomerization.^111,112^ This conformational impact of *N*-methylation has been demonstrated to enhance both the permeability and chameleonicity of cyclic peptides.^110,112,112,113^

The Lokey group investigated the effects of *N*-methylation and β-branching using the scaffold of sanguinamide A (**72a**, Table 53) as a model, which features two amide NH groups involved in intramolecular HB.^114^ Comparisons of the solubility and permeability of sanguinamide A (**72a**) and its four *N*-methylated derivatives revealed position-dependent effects (Table 53). Specifically, methylation at the 3-position (**73c**) significantly enhanced permeability, while the solubility increased only 2-fold. In contrast, methylation at the 2-position (**73a**), where the NH group is involved in intramolecular HB, markedly improved solubility but reduced permeability. This outcome is consistent with the disruption of intramolecular HB by *N*-methylation, which alters the conformation, increases the number of solvent-exposed amide bonds, and disrupts chameleonicity driven by intramolecular HB. The substantial solubility improvement arising from 2-position methylation also suggests that the closed form of the molecule predominates in aqueous environments, raising questions about the extent to which chameleonicity influences druglikeness. In contrast, *N*-methylation at the 3-position, which does not involve intramolecular HB, may reduce the 3D PSA without inducing significant conformational changes.

**Table 53:**
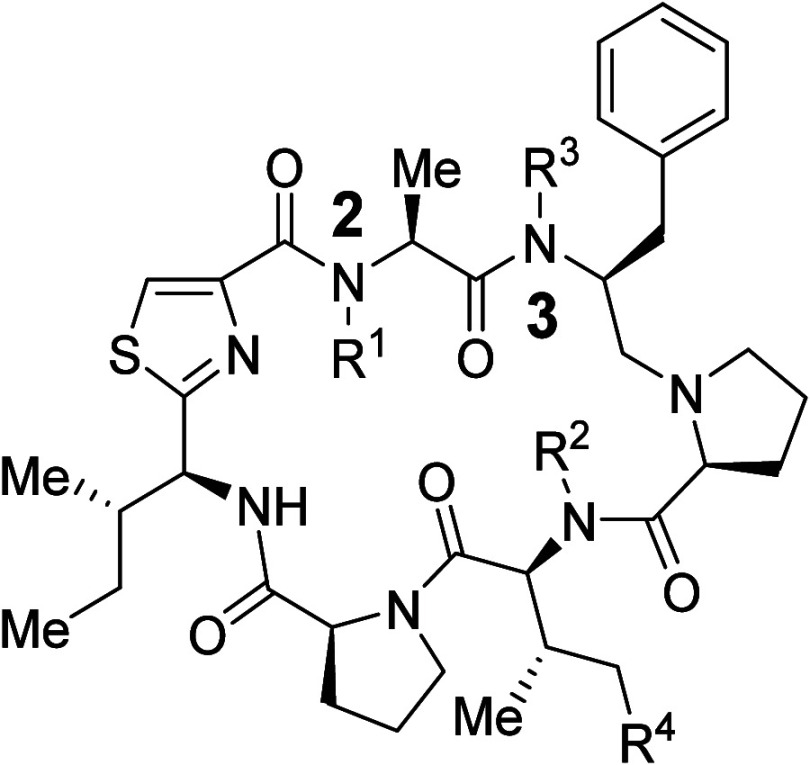
Impact of Sanguinamide A *N*-methylation on the Physicochemical Properties and Druglikeness

compd	*R* ^1^	*R* ^2^	*R* ^3^	*R* ^4^	thermodynamic aqueous solubility (μM)^a^	ALogP	HLPC retention time (min)	PAMPA (10^–6^ cm s^–1^)	Caco-2 (10^–6^ cm s^–1^)	LHSA^b^ (Å)	-ΔG_H20_ ^c^ (kcal mol^–1^)
**72a**	H	H	H	Me	107	2.86	2.77	5.3	1.3	221	24.8
**73a**	Me	H	H	Me	801	3.07	1.48	0.5	0.2	228	24.3
**73b**	H	Me	H	Me		3.07	1.79	1.3	0.4		
**73c**	H	H	Me	Me	172	3.07	3.03	11.0	7.4	340	23.6
**73d**	H	H	Me	H	296	2.61	2.24	9.1	6.8		

a Thermodynamic solubility in PBS.

b Largest hydrophobic surface area.

c Desolvation energy.

Similarly, the Fairlie group demonstrated that *N-*methylation of sanguinamide A induces position-dependent changes in conformation and the largest hydrophobic surface area (LHSA), with permeability positively correlating with LHSA (Table 53).^115^ They proposed that a larger hydrophobic surface is associated with a lower desolvation energy (Figure 12a) compared to that of a polar surface (Figure 12b), resulting in higher passive membrane permeability.

**Figure 12:**
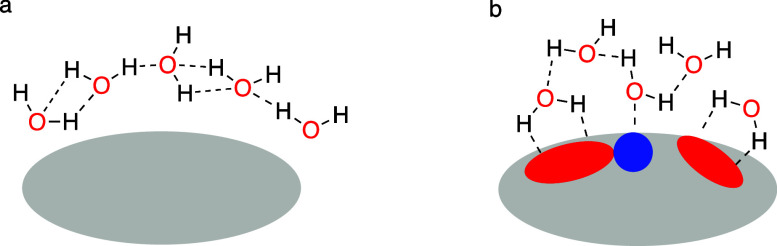
Models of water networks. (a) Methylation of cyclic peptide increases the hydrophobic surface (gray) having minimal interactions with water, lower desolvation energy, and higher passive membrane permeability. (b) Hydrophobic surface (gray) punctuated by polar atoms (red and blue) that interact with water, raising the desolvation energy and reducing passive membrane permeability.

According to Lokey et al., *N*-methylation at positions involved in intramolecular HB should be avoided to enhance the permeability. Rational methods for identifying solvent-exposed amide NH groups are therefore needed. To address this, Craik et al. investigated amide NMR chemical shift temperature coefficients (ΔδNH/ΔT).^116^ They found a correlation between ΔδNH/ΔT and hydrogen–deuterium (H-D) exchange rates in model cyclic hexapeptides. Backbone amide NHs with ΔδNH/ΔT values below −4.6 ppb/K typically exhibited rapid H-D exchange rates, indicating their lack of involvement in intramolecular HB. Based on this finding, the authors performed an *N*-methylation scan of backbone amide NHs in a model cyclic hexapeptide (Table 54). *N*-methylation at NHs with ΔδNH/ΔT values below −4.6 ppb/K (e.g., **74h**) significantly improved the Caco-2 permeability, demonstrating the utility of this method for predicting solvent-exposed NHs. Using this approach, they identified an orally available cyclic peptide with an oral bioavailability (*F*) of 33%.

**Table 54:**
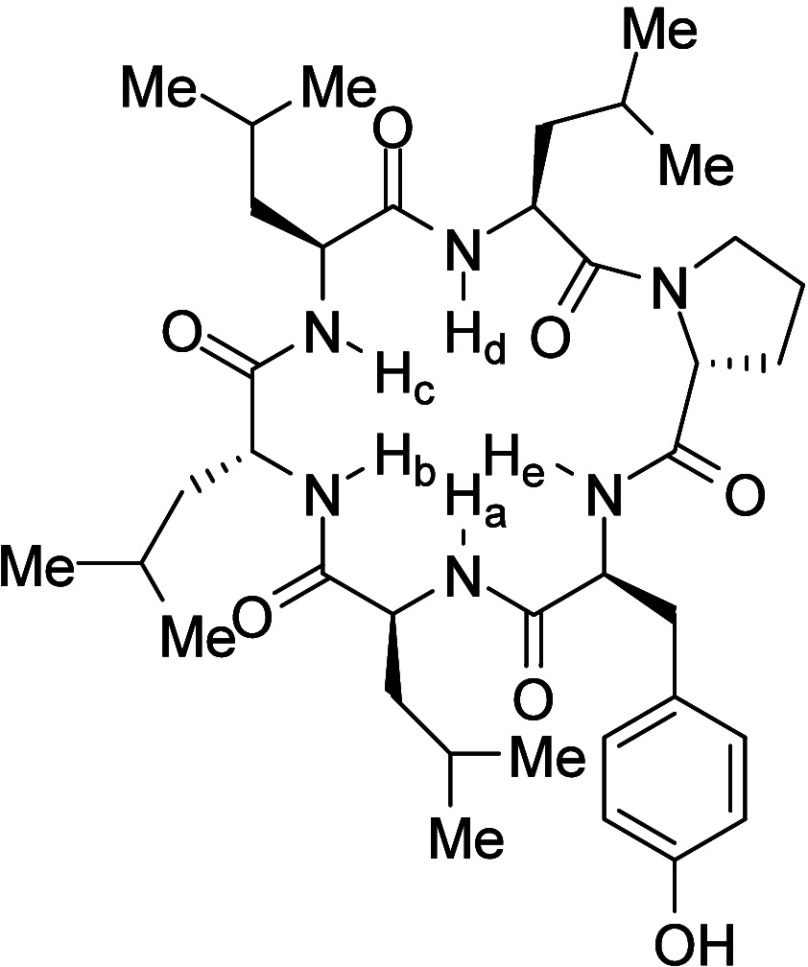
Amide Temperature Coefficient (ΔδNH/ΔT) and Caco-2 Permeability of *N*-methylated Peptides

	ΔδNH/Δ*T* value of each position					
cyclic peptide	NHa	NHb	NHc	NHd	NHe	Caco-2 *P*_app_ (10^–6^ cm s^–1^)
**74a**	–0.93	–7.74	–8.29	–5.69	–8.01	1.09
**74b**	–1.80	NMe	–6.90	–1.58	–6.44	1.68
**74c**	–4.40	–8.02	NMe	–4.10	–7.20	7.83
**74d**	–0.42	–7.76	–7.34	–3.84	NMe	9.13
**74e**	–2.19	NMe	NMe	–0.76	–6.96	10.76
**74f**	–1.69	NMe	–7.21	–1.24	NMe	7.75
**74g**	–0.60	–8.25	NMe	–2.94	NMe	11.33
**74h**	–1.75	NMe	NMe	–0.23	NMe	15.92
**74i**	NMe	–5.24	–6.82	–7.18	–7.70	1.13
**74j**	NMe	–5.24	–6.04	–3.18	NMe	5.18

Ester bonds in cyclic peptides have also been recognized as structural features that enhance membrane permeability, given their prevalence in membrane-permeable natural products and their impact on conformation.^109^ In 2021, it was reported that amide-to-ester substitutions in the ligand linkages of heterobifunctional PROTAC structures improved membrane permeability (see Section 4.3.1).^117^ In 2023, the collaborative group of Sando, Morimoto, and Lokey evaluated the effect of amide-to-ester substitutions on cyclic peptide permeability.^118^ Using a single-ester/*N*-methyl scan of amide bonds in a cyclic hexapeptide, they found that ester-substituted cyclic peptides exhibited higher permeability than their *N*-methylated counterparts. Their experimental and computational analyses suggested that the increased lipophilicity of ester bond-containing macrocycles contributes to this improvement. Another factor identified was the difference in conformation between ester-bonded derivatives and *N*-methylated analogs. While ester-bonded derivatives retained conformations similar to those of the original cyclic peptides, *N*-methylation altered the conformation, potentially generating new solvent-exposed amide NHs and reducing permeability.

However, ester-bond substitutions in larger cyclic peptides did not consistently enhance permeability to the same extent as *N*-methylation. These findings highlight the need for further studies to determine the conditions under which ester-bond substitutions are most effective.

#### 4.2.4 Desirable Properties for bRo5 Cyclic Peptides Learned from CsA

A research group from Chugai Pharmaceuticals identified key parameters for designing druglike bRo5 cyclic peptides.^119^ They focused on the properties of CsA and evaluated the druglikeness of 553 cyclic peptides with characteristics similar to those of CsA (Table 55).

**Table 55:** Comparisons of Structural Features and Physicochemical Parameters of 553 Cyclic Peptides and CsA

	553 peptides		
properties	min	Max	CsA
no. of amino acids residues	8	12	11
no. of *N*-alkyls	0	8	7
CLogP	4.4	15.2	14.4
no. of OHs	0	1	1
MW	845	1499	1203

Their analysis revealed three desirable parameters for cyclic peptides to exhibit favorable druglikeness: (1) 9 to 11 residues, (2) 6 or more *N*-alkylated amino acid residues (NAAs) in the peptide backbone, and (3) a CLogP value of 12.9 or higher (Table 56). Using these criteria, they constructed a second library of 11-residue cyclic peptides with nine randomized amino acids, ensuring that 65% of the peptides theoretically contained the desired number of NAAs. While CLogP values varied among peptides in the library, this was not considered problematic, as lipophilicity can be fine-tuned by modifying side-chain structures without significantly altering the conformation. Screening this library led to the discovery of the KRAS inhibitor AP8784 (**75**), whose optimization resulted in LUNA18 (**76**), a compound undergoing phase I clinical trials as of December 2024 (Tables 56 and 57).

**Table 56:**
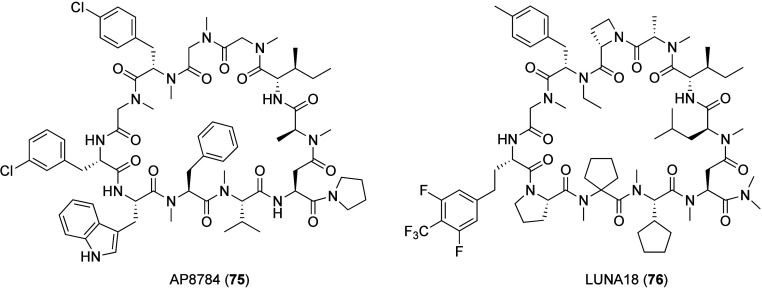
Desirable Structural Features and CLogP of Cyclic Peptides for Favorable Druglikeness and the Values of AP8784 and LUNA18

	no. of residues	no. of *N*-alkyls	CLogP
desirable values for favorable druglikeness	9 to 11	6 or more	12.9 or more
AP8784 (**75**)	11	7	12.7
LUNA18 (**76**)	11	8	14.5

**Table 57:** PK Parameters of LUNA18 (**76**)

	mouse		rat		dog		monkey	
route	IV	PO	IV	PO	IV	PO	IV	PO
dose (mg kg^–1^)	1	10	1	10	0.3	0.3	0.165	3
parameter								
AUC_inf_ (ng h mL^–1^)	1700	3600	3400	740	4100	2000	680	3400
CL (mL min^–1^ kg^–1^)	9.8		5.0		1.3		4.2	
V_ss_ (L kg^–1^)	2.6		0.96		0.99		1.2	
*T*_1/2_ (h)	5.5	3.5	5.2	3.6	14	15	8.0	8.5
*C*_max_ (ng mL^–1^)		780		1800		190		630
*T*_max_ (h)		2.0		2.0		2.0		2.3
*F* (%)		21		22		47		26

An integrated evaluation of the 553 cyclic peptides with CsA-like properties revealed that metabolic stability generally increased with the number of residues, whereas permeability was optimal for 8-, 9-, and 11-residue peptides but decreased for 10- and 12-residue peptides. Based on these findings, the authors identified 11 residues as the optimal number. This conclusion aligns with the theory of chameleonicity: hydrophilic open conformations in aqueous environments can suppress oxidative metabolism, while lipophilic closed conformations enhance the membrane permeability. The case of LUNA18, which exhibits both favorable solubility and permeability, further supports this hypothesis.

Finally, the authors proposed that these desirable properties could be generalized and applied to other cyclic peptides and bRo5 molecules. They translated their findings into two general criteria: (1) a CLogP-to-residue ratio of 1.17 or higher and (2) an *N*-alkylation ratio of 0.5 or higher. This study raises the possibility that integrated investigations of other bRo5 molecules, such as large peptide-mimetic protease inhibitors (e.g., HIV inhibitors) and heterobifunctional compounds such as PROTACs, might yield other criteria for optimal properties.

### 4.3 Druglikeness Studies of PROTACs

PROTACs are compounds that induce the degradation of a protein of interest (POI) by leveraging the ubiquitin–proteasome system.^120,121^ PROTACs possess heterobifunctional structures, consisting of a small-molecule (or peptidic) ligand targeting the POI linked to another small-molecule (or peptidic) ligand that binds to an E3 ubiquitin ligase, thereby bringing the POI and E3 into close proximity. The success of PROTACs has established this induced-proximity-based strategy as a generalizable approach for drug design, leading to the emergence of other “TAC (targeting chimera)” technologies. Since the first report on PROTACs in 2001, they have gained recognition as a promising modality for drug discovery, with over 25 PROTACs now in clinical trials.^121^ This growing interest underscores the importance of studying the druglikeness of PROTACs. Medicinal chemistry optimizations of PROTACs have demonstrated that their maximum activity can be achieved at picomolar concentrations, despite the individual binding affinities of their warheads being in the nano- to micromolar range.^122,123^ This difference is attributed to their catalytic mechanism, which allows them to function effectively at doses lower than their binding affinities might suggest. Furthermore, low doses are preferred to mitigate the hook effect, where excessive doses reduce the efficacy. However, PROTACs typically have MW ranging from 800 to 1200 Da,^124^ resulting in low aqueous solubility (often in the low micromolar range) and limited membrane permeability (<1.0 × 10^–6^ cm/s), both of which are commonly classified as poor. Consequently, although improving biological activity is crucial, enhancing the bioavailability of PROTACs represents a significant challenge.

This section summarizes studies focused on the solubility and permeability of PROTACs as well as strategies for their improvement.

#### 4.3.1 Evaluation of Physicochemical Properties of PROTACs

Extensive research has been conducted on the relationships between the physicochemical properties that govern the aqueous solubility and membrane permeability of PROTACs and the druglikeness of PROTACs. The Caron group identified key determinants of the PROTAC solubility, demonstrating a correlation between solubility and lipophilicity. They measured the physicochemical properties of 21 commercially available PROTACs and plotted experimental solubility (Log*S*) against other properties.^125^ They found that Log*S* vs BRLog*D* and Log*S* vs Marvin Log*P* showed promising linear correlations, with *r* ^2^ values of 0.67 and 0.69, respectively.

However, plots of Log*S* against polarity parameters, such as Log*k*_W_^IAM^ and PSA, showed poor correlations, despite polarity being a known factor influencing the solubility of cyclic peptides (see Section 4.2.1). The logarithm of the experimental chromatographic retention factor (Log*k*_W_^IAM^), determined using an immobilized artificial membrane (IAM) column, serves as an indicator of molecular polarity but did not correlate strongly with solubility in this context.

Further support for the findings by Caron et al. comes from the work of Harling et al., who optimized RIPK2-targeting PROTACs by reducing their lipophilicity. For instance, replacing the E3 ligand moiety in **77a** with a less lipophilic ligand (**77b**, Table 58) significantly improved solubility.^126^ Additionally, substituting the POI ligand in **77b** with another less lipophilic ligand (**77c**) resulted in a further enhancement of the solubility.

**Table 58:**
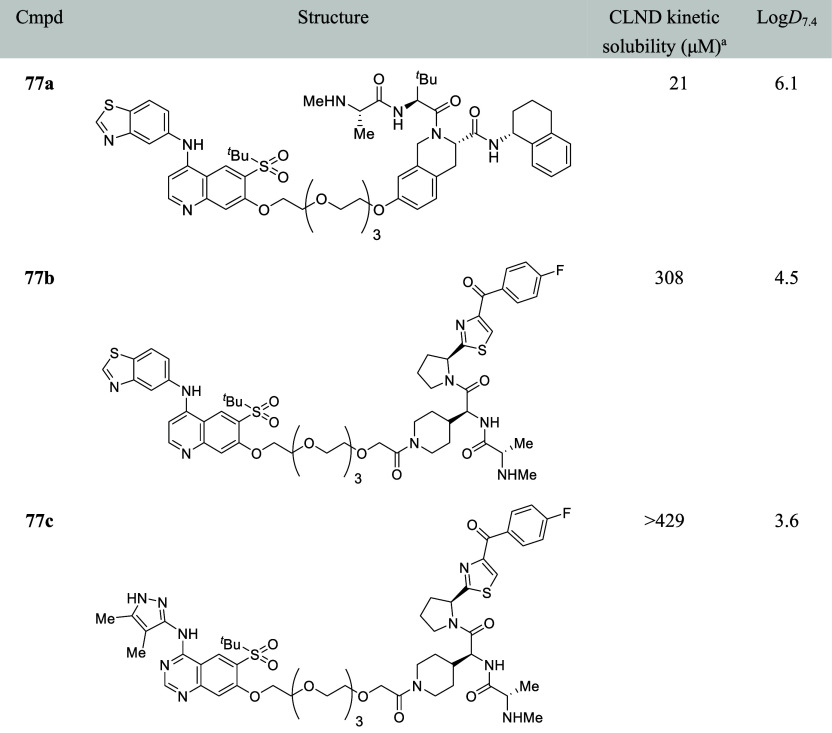
Structures of RIPK2 PROTACs and Their Binding Potency, Solubility, and Lipophilicity Parameters

a CLND, chemiluminescent nitrogen detection.

A collaborative group from Boehringer Ingelheim and the University of Dundee reported that introducing a methyl group (**78b**) into the linker of PROTAC **78a** resulted in an over 18-fold improvement in aqueous solubility at pH 4.5, despite an increase in CLogP. Additionally, this modification led to a more than 12-fold enhancement in Caco-2 permeability (Table 59).^127^ However, their analysis revealed no clear correlation between CLogP and solubility or permeability among the PROTAC analogs studied (Figure 13). This finding suggests that simple lipophilicity descriptors, such as CLogP, are insufficient to fully explain the relationship between lipophilicity and druglikeness in PROTACs, and additional factors must be considered.

**Table 59:**
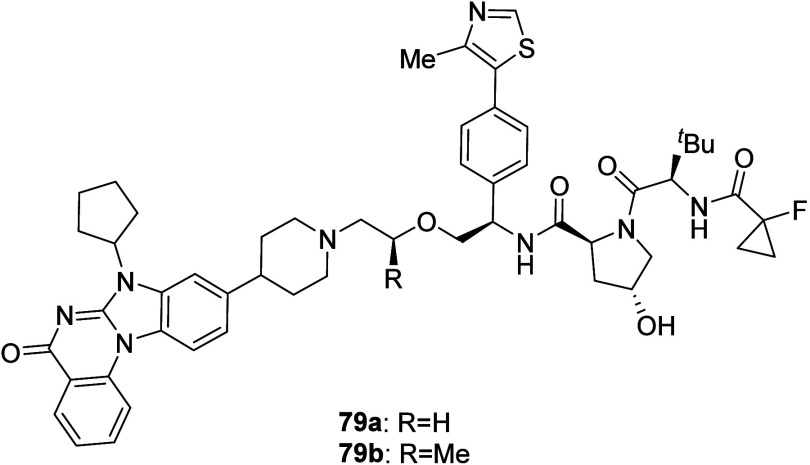
CLogP Itself Does Not Necessarily Determine the Solubility and Permeability of PROTACs

compd	kinetic aqueous solubility (pH 4.5) (μg mL^–1^)^a^	CLogP	Caco-2 *P*_app_ (10^–6^ cm s^–1^)
**78a**	<1	8.9	<0.5
**78b**	18	9.2	6

a Solubility was determined by dilution of a 10 mM DMSO solution into a buffer to a final concentration of 125 μg/ml. The mixture was incubated for 24 h, followed by filtration and analysis of the filtrate by LC-UV.

**Figure 13:**
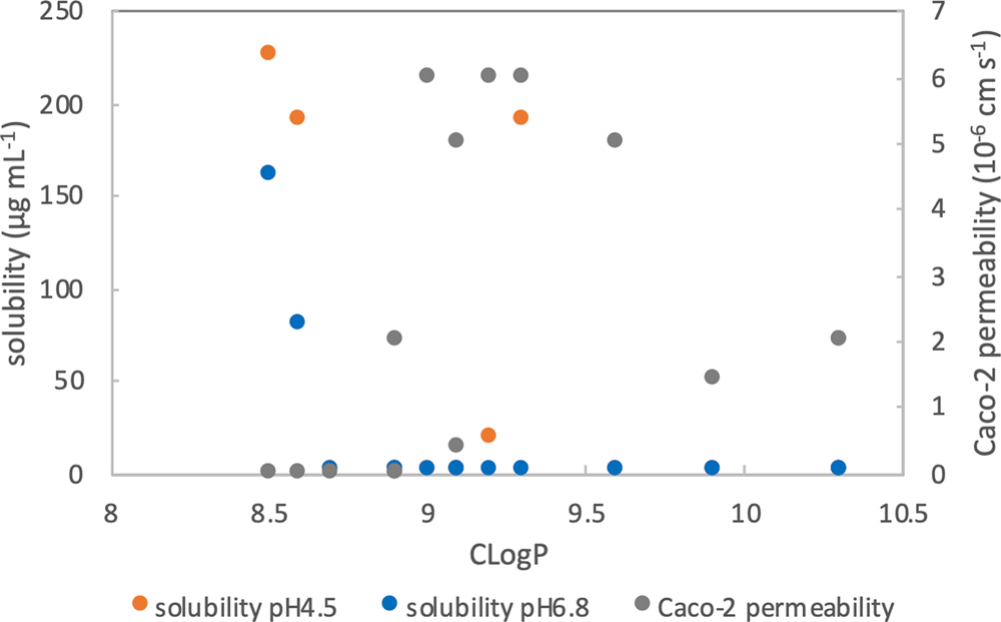
Plot of solubility versus permeability of the PROTACs. There appears to be no correlation between solubility and permeability.

Kihlberg and colleagues also demonstrated that, even among a few PROTACs with different linkers, CLogP did not correlate with solubility or membrane permeability (Table 60), further supporting the need for alternative descriptors.^102^ Their NMR and MD analyses revealed that the most lipophilic, yet most water-soluble, analogue **79c** adopts a more elongated and loosely folded conformation in CHCl_3_ compared to **79a** and **79b**. These findings suggest that the 3D structure of PROTACs significantly influences their druglikeness and that calculated lipophilicity parameters must account for these structural factors. Moreover, they identified a correlation between the radius of gyration (*R*_gyr_) in CHCl_3_, representing a lipophilic environment, and the solvent-accessible 3D polar surface area (SA-3D PSA). Based on these results, they concluded that molecular flexibility is a critical property, enabling PROTACs to adopt folded conformations with low SA-3D PSA, which is associated with high cell permeability.

**Table 60:**
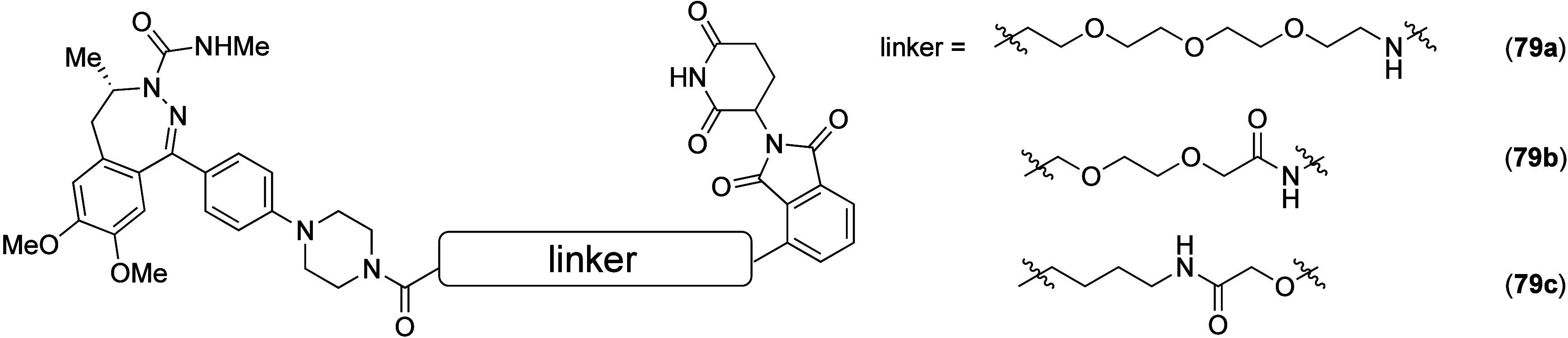
Linker SAR of PROTACs Suggests Lipophilicity Does Not Correlate with Druglikeness^a^

										SA-3D PSA (Å^2^)		*R*_*gyr*_ (Å)	
cmpd	MW	HBD	HBA	Solubility (mg L^–1^)	CLogP	Caco-2 (nm s^–1^)	PAMPA (−Log*P*_e_, cm s^–1^)	tPSA (Å^2^)	NRotB^b^	NAMFIS^c^	MD simulation	NAMFIS^c^	MD simulation
**79a**	897	3	12	56	1.50	30	6.56	210	21	209 (*S*), 167 (*R*)	218	5.40 (*S*), 5.16 (*R*)	7.84
**79b**	853	3	12	31	1.26	11	n.d.^d^	218	17	232.26 (*S*), 245.84 (*R*)	277	5.44 (*S*), 5.60 (*R*)	6.94
**7c**	851	3	11	63	2.56	6	>7.37	209	17		300		7.96

a Rather, the actual molecular size (*R*_*gyr*_) and polarity (SA-3D PSA) in the solution appear to be crucial.

b Number of rotational bond.

c NAMFIS (NMR analysis of molecular flexibility in solution), population weighted mean; *S* and *R* in brackets indicate the stereochemistry of thalidomide.

d Not determined.

Garon et al. evaluated the physicochemical properties of several PROTACs using chromatographic techniques and identified a strong correlation (*r* ^2^ = 0.85) between Caco-2 permeability (Log*P*_app_) and an experimental molecular polarity descriptor, ΔLog*k*_W_^IAM^.^128^ ΔLog*k*_W_^IAM^ represents the difference in Log*k*_W_^IAM^ values, where higher ΔLog*k*_W_^IAM^ indicates greater molecular polarity. Interestingly, no correlation was found between the calculated PSA values and ΔLog*k*_W_^IAM^, suggesting that experimental polarity (ΔLog*k*_W_^IAM^) is a better measure of PROTAC polarity. This discrepancy highlights the limitations of computed PSA values arising from conformational factors, as discussed earlier.

In a collaborative study by the Lokey and Ciulli groups, the permeability and other properties—such as MW, lipophilicity, and polarity—of 11 PROTACs were measured and compared to identify determinants of permeability.^129^ The results suggested that physicochemical properties other than MW significantly influence the PROTAC permeability. For example, the PROTACs with the highest and lowest MWs (**81** and **82c**) exhibited the lowest permeability (Table 61). Moreover, compounds with nearly identical properties displayed stark differences in their permeability. For instance, PROTACs **80b** and **82b** showed a 10-fold difference in permeability despite having almost the same properties. Similarly, a comparison of PROTACs **80a** and **82a**, which also have nearly identical properties, revealed that the permeability of **80a** was 120-fold higher than that of **82a**.

**Table 61:**
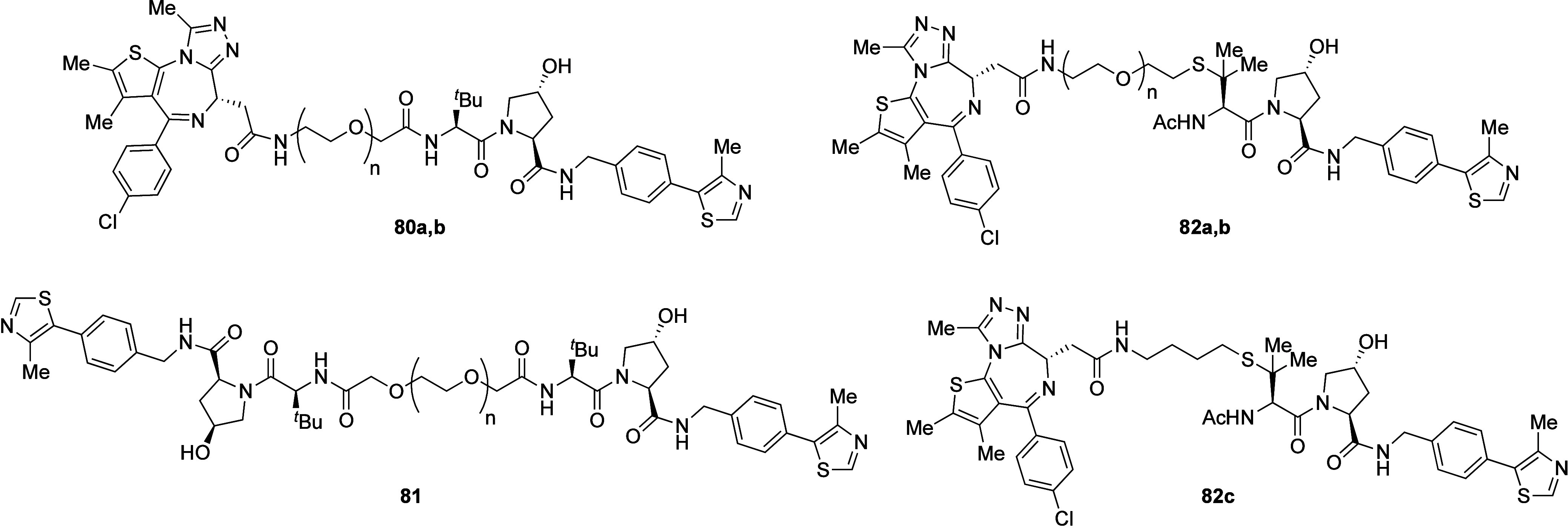
Structures of PROTACs and a Dimer of the VHL Ligand, Together with Physicochemical Parameters

compd	*n*	MW	HBD	HBA	Log*D*	ALogP	LPE	PAMPA (10^–6^ cm s^–1^)
**80a**	2	959	4	11	–1.1	3.7	0.5	0.6
**80b** (MZ1)	3	1003	4	12	–1.6	3.6	0.1	0.03
**81**	5	1179	6	16	–3.2	0.6	1.6	0.002
**82a**	1	961	4	11	–3.8	3.7	–2.3	0.005
**82b**	2	1005	4	12	–4.3	3.6	–2.7	0.003
**82c**		945	4	10	–3.8	4.5	–3.1	0.002

The authors utilized LPE to explain these results. For example, in the comparisons between compounds **80a** and **82a** and between **80b** and **82b**, despite the similar Alog*P* values, identical numbers of HBDs/HBAs, and nearly identical MW of each pair, the membrane permeability differed by a factor of 10. However, when the difference in the LPE (ΔLPE) was calculated, both comparisons yielded a value of 2.8. It is known that the addition of one solvent-exposed amide NH decreases the LPE by approximately 1.8. Based on this information, the authors hypothesized that the significant differences in membrane permeability were due to solvent exposure of the amide NH in the penicillamine-type compounds **82a** and **82b**, whereas the NH remained masked in *t*-Leu-containing compounds **80a** and **80b**.

Additionally, a comparison of the linkage between the von Hippel-Lindau (VHL, an E3 ligase) ligand and the linker revealed that ester compounds **80c** and **83b** exhibited better PAMPA than their amide counterparts **80b** and **83a**. Notably, the LPE values for the ester compounds were comparable to or higher than those of the amides (Table 62). Based on these findings, two design strategies were proposed for improving PROTAC permeability: (1) ether oxygen in PEG linkers may form intramolecular HB, shielding the amide NH in PROTAC molecules, and (2) replacing solvent-exposed amide bonds with ester bonds can enhance membrane permeability.

**Table 62:**
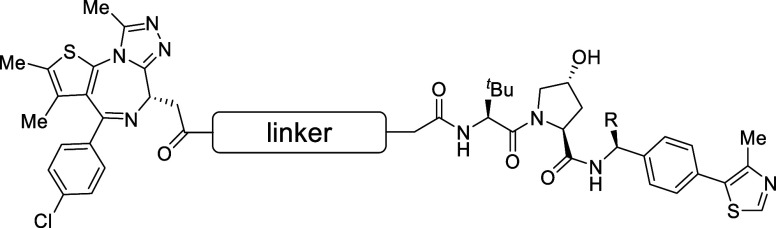

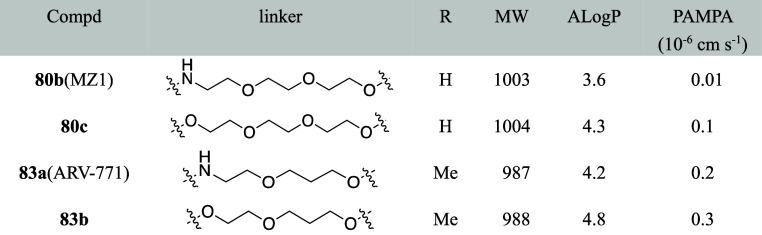
Impact of Amide-to-Ester Linkage Switching on Activity, Lipophilicity, and Membrane Permeability

Focusing on these strategies, the collaborative groups investigated the impact of amide-to-ester conversion on PROTAC properties, including permeability (for the case of cyclic peptides, see Section 4.2.3).^117^ They found that amide-to-ester substitutions in model bifunctional molecules with Alog*P* values between 1 and 4 improved PAMPA. However, this effect was not observed in compounds with Alog*P >* 4. There was also a positive correlation with MDCK cell permeability, although ester-containing compounds exhibited higher efflux ratios than their amide counterparts. Building on these results, they applied amide-to-ester conversion to known BET bromodomain-targeting PROTACs **80b** (MZ1) and **83a** (ARV-771). This modification led to improved PAMPA (Table 62) and enhanced the BRD4 degradation activity.

Pirali et al. reported that PROTAC **84b**, designed using the amide-to-ester strategy, did not exhibit improved solubility but was very stable during incubation with MLM (Table 63).^130^ These findings suggest that the amide-to-ester conversion strategy may be effective not only for cyclic peptides but also for PROTACs and potentially other bRo5 drugs.

**Table 63:**
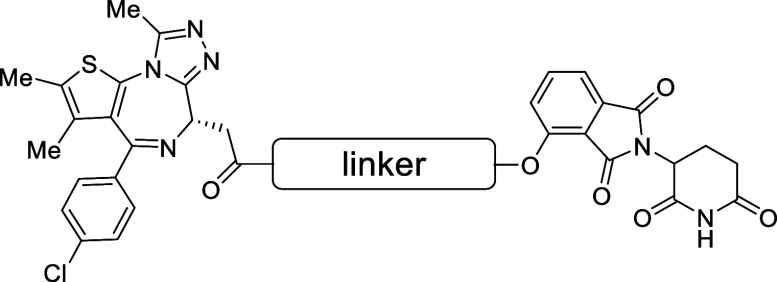

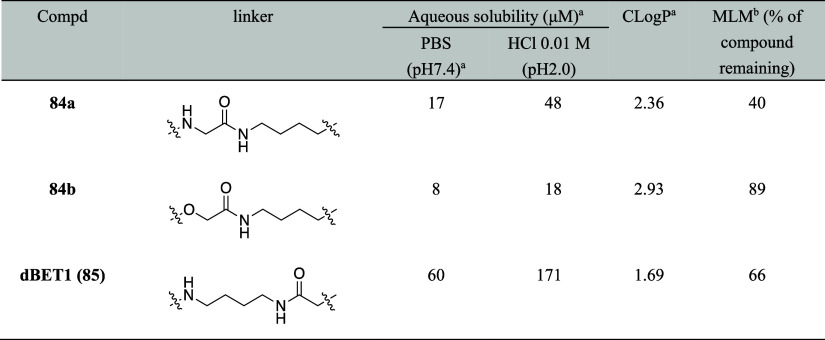
Impact of Amide-to-Ester Linkage Switching on the Solubility, Lipophilicity, and Metabolic Stability

a Calculated with ChemDraw ultra 20.0.

b Residual substrate after 1 h of incubation with MLM in the presence of NADPH.

#### 4.3.2 Chameleonicity/Flexibility in PROTACs

Kihlberg et al. focused on the conformational changes of PROTACs in different environments and proposed that the chameleonicity of PROTACs is a key behavior enabling their permeation through lipid bilayers.^131^ Their study demonstrated that PROTAC **86** (Figure 14) adopts a higher population of closed, low-3D PSA conformations in CHCl_3_ compared to DMSO–water. This finding suggests that PROTACs favor open conformations in polar environments, such as extracellular and intracellular compartments, in order to form HBs with water while adopting closed conformations in apolar environments, such as lipid bilayers, to shield hydrophilic groups. As in the case of cyclic peptides, this behavior allows PROTACs to adjust their polarity in response to the environment, facilitating membrane permeability. Since this chameleonic behavior depends on intramolecular HBs and molecular flexibility, the plasticity of linker structures is likely a critical factor influencing chameleonicity and cell permeability.

**Figure 14:**
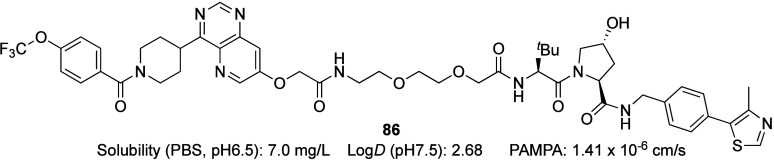
Structure and properties of PROTAC **86**.

The Kihlberg group further investigated the relationship between linker structure and cell permeability using three PROTACs with identical POI and E3 ligands but different linker structures, employing NMR structural analysis and MD simulations (Table 60 in Section 4.3.1). *In vitro* permeability analyses revealed that PROTAC **79a**, which features a longer PEG-based linker, exhibited the highest cellular permeability. The study yielded three conclusions. (1) For linkers of the same length, alkyl linkers are somewhat more flexible than PEG-based linkers. (2) PEG-based linkers are more likely to adopt folded, low-3D PSA conformations than alkyl linkers, due to the inherent gauche effect of PEG structures, in contrast to the anticonformation favored by alkyl linkers. (3) Among PEG-based linkers, longer linkers are more flexible and more likely to populate folded conformations than shorter ones. These findings are consistent with the superior permeability of PROTAC **79a** compared to other PROTACs in the study.

However, studies on the druglikeness of PROTACs with varying linker structures, as discussed in the following section, indicate that flexible linkers are not always optimal. Thus, chameleonicity should be considered as one of several parameters influencing the druglikeness of PROTACs, rather than a sole determinant.

#### 4.3.3 Linkerology in PROTACs Focusing on Druglikeness

In the PROTAC design, the modification of E3 ligands and POI ligands is often constrained by limitations in linkage positions and acceptable structural variations. Consequently, the linker moiety has become a primary target for customization to improve both physicochemical properties and biological activity.^132^ This has led to an increased focus on SAR studies of linkers, termed linkerology, which seek to elucidate the relationships between linker structures and PROTAC activities or properties. Cyclic linker structures, such as piperazine moieties, have gained particular attention for enhancing the druglikeness of PROTACs following the disclosure of clinical trial PROTAC structures that frequently include cyclic linkers. Several studies have demonstrated the impact of the linker design on the solubility and permeability of PROTACs. For example, the physicochemical properties of PROTACs composed of cereblon binders, the BRD4 inhibitor JQ1, and various linkers were comprehensively evaluated (Table 64).^133^ All PROTACs in this study exhibited thermodynamic aqueous solubility ranging from 32.3 to 78.5 μM, which is similar to or slightly lower than the solubility of their conjugating ligands, such as JQ1 (52.1 μM), thalidomide analogs (55.7–84.6 μM), and phenyl glutarimide (PG) analogs (76.1–148.5 μM), another class of cereblon binders. In a comparison of linkers, alkyl linkers (**87a**, **87b**) showed a slightly higher solubility than the triazole linker (**88c**). As regards permeability, all PROTACs exhibited low Caco-2 permeability (around or below 2 × 10^–6^ cm/s). For thalidomide-type PROTACs, oxygen-linked compounds (**88a**, **88c**) demonstrated 7.3- and 6.7-fold higher permeability, respectively, than nitrogen-linked compounds (**88b**, **88d**). This difference is likely due to the additional HBDs present in **88b** and **88d**. Among PG-type PROTACs, 4-substituted compounds (**87a**, **87b**) showed higher permeability than 3-substituted ones (**87c**, **87d**) and were comparable to those of the well-known thalidomide-based PROTAC dBET1 (**85**). Notably, two 5-substituted cereblon-based PROTACs, ARV-471 and ARV-110, are orally available, suggesting that conjugation at positions distant from the glutarimide core in cereblon binders may enhance permeability.

**Table 64:**
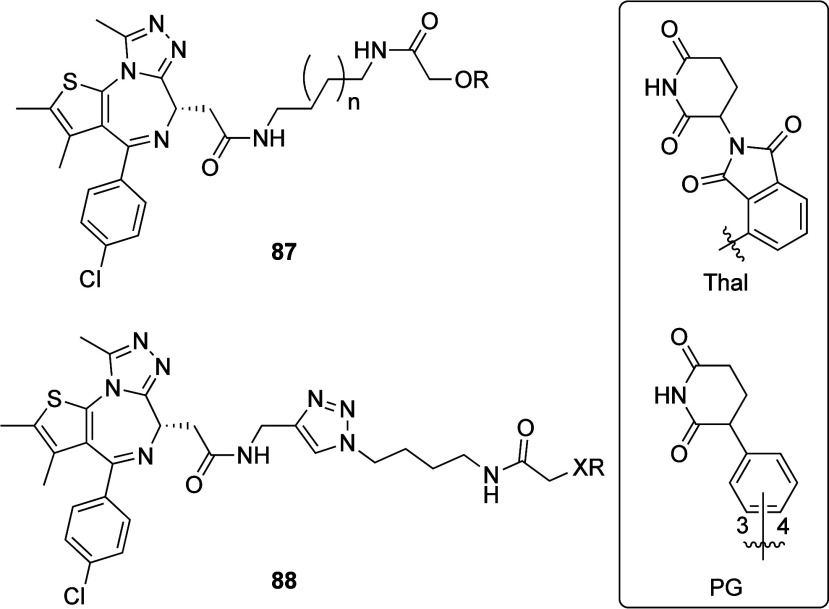
Linker Structure–Property Relationships of the PROTACs

cmpd	*n*	*R*	*X*	thermodynamic aqueous solubility (μM)	Caco-2 (10^–6^ cm s^–1^)
**85** (dBET1)	1	Thal	—	53.5	1.16
**87a**	1	4-PG	—	78.5	1.66
**87b**	2	4-PG	—	78.3	1.43
**87c**	1	3-PG	—	65.3	0.1
**87d**	2	3-PG	—	65.3	0.97
**88a**	—	Thal	O	50.8	1.72
**88b**	—	Thal	NH	32.3	0.22
**88c**	—	4-PG	O	60.4	2.14
**88d**	—	4-PG	NH	56.8	0.32

Piperazine is frequently used as a linker moiety in PROTACs with favorable druglikeness. In a study by Pirali et al., PROTAC **89** (Figure 15) showed remarkable solubility (5034 μM) in 0.01 M HCl (pH 2.0).^130^

**Figure 15:**
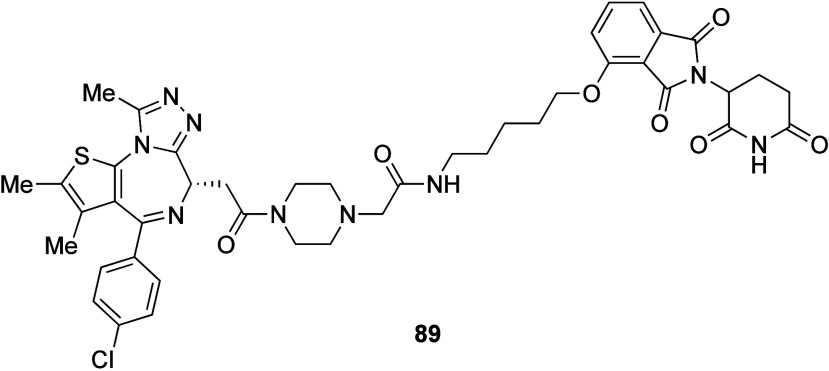
Structure of PROTAC entry **89**.

In a linker SAR study conducted by Harling et al., replacing PEG-based linkers with more rigid heteroaromatic/aromatic or piperazine/piperidine tandem linkers generally reduced solubility (Table 65).^130^ Their analysis revealed the following trends. (1) Increasing the number of atoms in the linker decreased solubility (**90b** vs **90c** vs **90d**). (2) Piperazine linkers exhibited better solubility than piperidine linkers, likely due to their lower lipophilicity (**90c** vs **90e**, **90d** vs **90f**). (3) Solubility was not significantly affected by the type of heteroaromatic ring (**90f** vs **90g** vs **90h** vs **90i**).

**Table 65:**
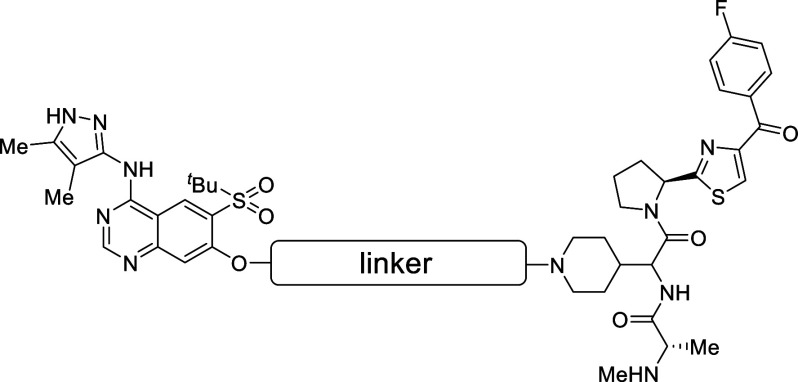

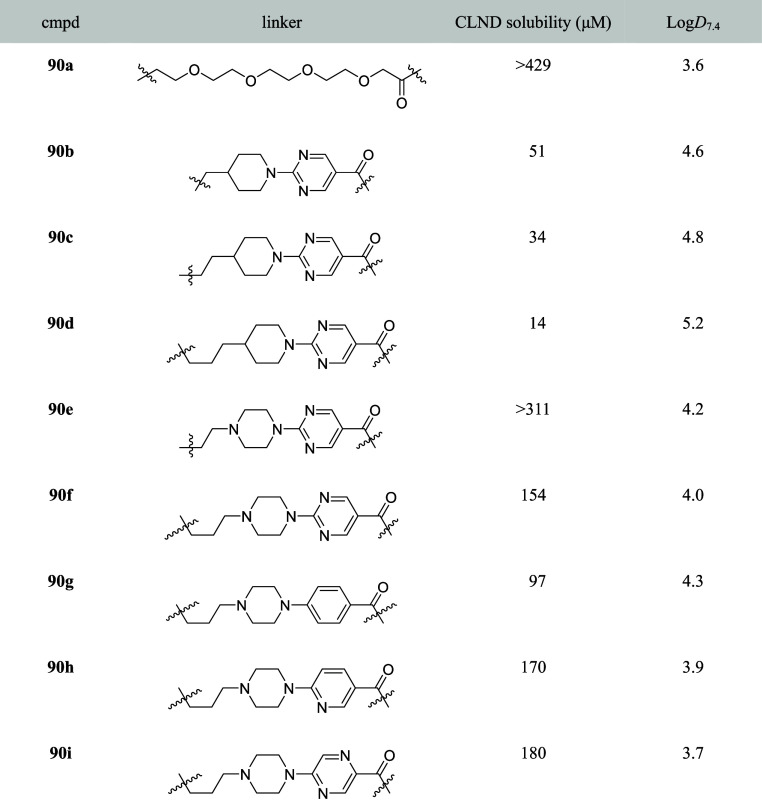
Linker Structure–Property Relationship Study Focusing on Rigid Cyclic/Aromatic Linker Structures

Ciulli et al. improved Caco-2 permeability of PROTACs by modifying the linker structure.^134^ PROTAC **91a**, which possesses an ethylene glycol linker, exhibited poor passive permeability (A–B rate of 1.1 × 10^–7^ cm/s) and a high transporter-mediated efflux (B–A rate of 20.7 × 10^–6^ cm/s), resulting in an efflux ratio of 190 (Table 66). In contrast, PROTAC **91b**, with a 4-ethylbenzyl linker, showed improved permeability (A–B rate of 8.4 × 10^–7^ cm/s, B–A rate of 7.6 × 10^–6^ cm/s, and efflux ratio of 9.1). Interestingly, ACBI1 (**91c**), featuring a 4-oxyethoxybenzyl linker, showed an even greater permeability improvement, exhibiting an A–B rate of 2.2 × 10^–6^ cm/s, a B–A rate of 3.8 × 10^–6^ cm/s, and an efflux ratio of 1.7. Notably, ACBI1 differs from **91b** only in the addition of a single-oxygen atom. Despite the reduced CLogP due to this modification, the A–B rate significantly improved, while the B–A rate was reduced by approximately half. Although the detailed mechanisms underlying these changes remain unclear, these findings suggest that factors other than lipophilicity can play a critical role in determining permeability.

**Table 66:**
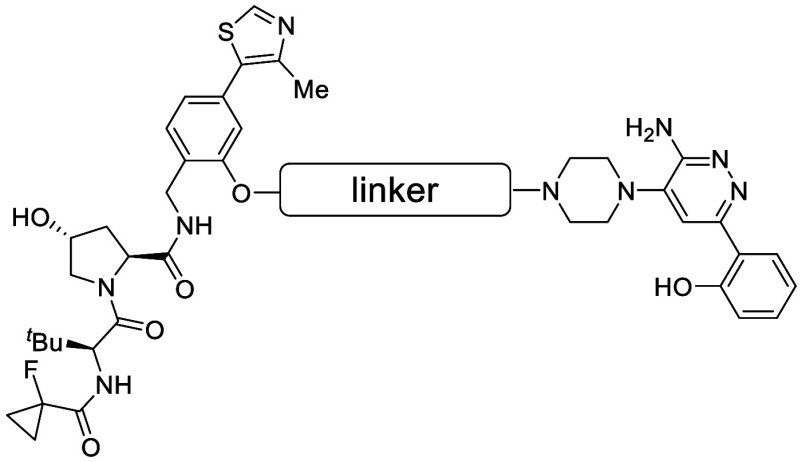

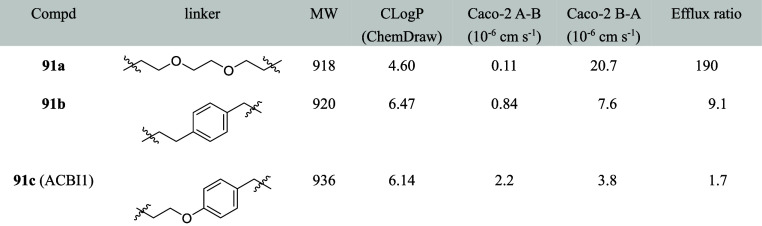
Impact of Linker Structure on Membrane Permeability and Efflux

Ciulli et al. reported the discovery of PROTAC XL01126 (**92**, Figure 16), an orally available (mouse bioavailability *F*: 15%) and BBB-permeable leucine-rich repeat kinase 2 degrader.^135^ This compound was identified through a comprehensive medicinal chemistry campaign. In that study, the authors investigated various linker structures, including flexible linkers (PEG, alkyl) and rigid linkers (aromatic, alkyne, and cyclic). Among the compounds examined, PROTAC XL01126, which incorporates a *trans*-1,4-cyclohexyl linker, exhibited the most potent activity as well as favorable pharmacokinetic profiles. This finding aligns with the observation that many orally available PROTACs feature sp^3^-cyclic linkers. While such linkers are less conducive to the conformational chameleonicity described earlier, this trend highlights the importance of NRotB in addition to chameleonicity in determining the druglikeness of PROTACs.

**Figure 16:**
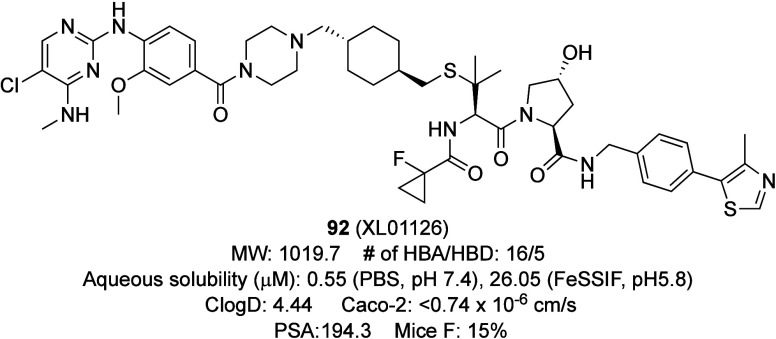
Structure and druglikeness of PROTAC XL01126.

## 5 Conclusions

Improvement of the aqueous solubility and permeability of lead compounds remains a critical challenge in drug discovery programs, especially for bRo5 compounds, including cyclic peptides and PROTACs, which are of increasing importance in modern medicinal chemistry. Medicinal chemists must navigate and reconcile complex and often contradictory parameters, such as lipophilicity versus solubility, solubility versus flatness, and solubility/permeability versus molecular weight.

Over the past two decades, significant advancements have been made to achieve Aufheben of such parameters of druglikeness. As for the apparently contradictory physicochemical properties of lipophilicity and aqueous solubility, there is increasing evidence that chemical modification to weaken intermolecular interactions can improve not only aqueous solubility but also permeability by increasing lipophilicity. Various molecular design strategies to enhance the aqueous solubility by weakening intermolecular interactions have been proposed. In particular, advances in synthetic methods for phenyl ring mimetics have promoted the achievement of Aufheben of phenyl spacers and druglikeness. In general, structural modifications undertaken to enhance aqueous solubility typically result in either increased activity (due to a better fit within the protein pocket) or decreased activity (if the fit is compromised), and the introduction of hydrophilic substituents often interferes with protein–drug interactions. In such cases, the introduction of hydrophobic group would provide an alternative approach for improving aqueous solubility (Table 1).

We have also presented concrete examples of strategies to increase the membrane permeability and aqueous solubility of bRo5 molecules. Because rationales and guidelines for adjusting PROTACs’ solubility and cell permeability are still limited, concise synthetic methodologies and an efficient assay of druglikeness have been developed.^136^ A further strategy for improving the cellular uptake of bRo5 molecules is the utilization of transmembrane proteins as enhancers.^137^ Overall, there are an increasing number of examples of bRo5 molecules that are both water-soluble and cell-permeable.

In recent years, medicinal chemistry has faced the challenge of addressing undruggable targets, in part because of the limited availability of drug targets and the expansion of modalities for intervention. While the complexity of this challenge continues to grow, medicinal chemistry has consistently demonstrated its ability to overcome contradictions and innovate. The pursuit of new opportunities for Aufheben—the resolution of opposing properties—will remain a driving force in shaping the future of the field.

## Abbreviations

BCHep: Bicyclo­[3.1.1]­heptanes
BCO: Bicyclo­[2.2.2]­octane
BCP: Bicyclo­[1.1.1]­pentane
BCS: Biopharmaceutics Classification System
BP: Bridged piperidine
CLND: Chemiluminescent nitrogen detection
CsA: Cyclosporine A
Do: Dose number
Fsp^3^: sp^3^-hybridized carbons
HBA: Hydrogen bond acceptor
HBD: Hydrogen bond donor
HLM: Human liver microsomes
IAM: Immobilized artificial membrane
LHSA: Largest hydrophobic surface area
Log*D*: Distribution coefficient
LPE: Lipophilic permeability efficiency
LYSA: Lyophilization solubility assay
MD: Molecular dynamics
MDCK: Madine–Darby canine kidney
MLM: Mouse liver microsomes
MW: Molecular weight
NAA: *N*-alkylated amino acid residue
NAMFIS: NMR analysis of molecular flexibility in solution
NRotB: Number of rotational bond
PAMPA: Parallel artificial membrane permeability assay
*P*_app_: Apparent permeability
PFI: Property forecast index
PG: Phenyl glutarimide
POI: Protein of interest
PPAR: Peroxisome proliferator-activated receptor
PPI: protein–protein interaction
PROTAC: Proteolysis targeting chimeras
PSA: Polar surface area
*R*_gyr_: Radius of gyration
Ro5: Rule of five
RRCK: Ralph Russ canine kidney
SA: Solvent-accessible
TAC: Targeting chimera
tPSA: Topological polar surface area
VSMC: Vascular smooth muscle cells
XRD: X-ray diffraction
Δ*G*_*sol*_: Gibbs-free energy of dissolution
Δ*H*_sol_: Enthalpy of dissolution
Δ*S*_sol_: Entropy of dissolution
